# Supplementary material for: t-SMILES: a fragment-based molecular representation framework for de novo ligand design
Source: Nat Commun. 2024 Jun 11;15:4993. doi: 10.1038/s41467-024-49388-6 (PMC11167009; doi:10.1038/s41467-024-49388-6)
Supplement: Supplementary file 1 — Supplementary Information [file 41467_2024_49388_MOESM1_ESM.pdf]

Supporting Information

t-SMILES: A Fragment-based Molecular Representation Framework for De Novo Ligand Design

Juan-Ni Wu[1], Tong Wang[1], Yue Chen[1], Li-Juan Tang[1], Hai-Long Wu[1]*, Ru-Qin Yu[1]*

State Key Laboratory of Chemo/Biosensing and Chemometrics, College of Chemistry and Chemical Engineering, Hunan University, Changsha 410082, People's Republic of China

Contents

A. Algorithms **4**

A.1 TSSA Algorithm and Example Molecules 4

A.1.1 TSSA Algorithm 4

A.1.2 Aspirin 8

A.1.3 Caffeine 9

A.1.4 Paracetamol 10

A.1.5 Molecule with Chirality 11

A.2 Algorithm 1: BFS 12

A.3 Algorithm 2: JTVAE Fragmentation Algorithm 12

A.4 Algorithm 3: BRICS, MMPA and Scaffold Fragmentation Algorithms in t-SMILES 12

A.5 Brief Overview of Related Fragmentation Algorithms 13

B. Experiments **16**

B.1 Generation Algorithms and Baseline Models 17

B.1.1 Setting and Baseline Models 18

B.1.2 Training and Repeatability 19

B.2 Datasets 22

B.3 Benchmarks 24

B.3.1 Standard Distribution-Learning Benchmarks 24

B.3.2 Physicochemical Properties 25

B.3.3 Goal-Directed Benchmarks 27

B.3.3.1 LSTM Models on ChEMBL for Goal Directed Benchmarks 28

B.3.3.2 Physicochemical Properties for LSTM Models on ChEMBL 29

B.3.3.3 Goal-Directed Benchmarks on ChEMBL 30

B.3.3.4 Goal-Directed Benchmarks for T16.SMPO 31

B.3.3.5 Goal-Directed Benchmarks for T16.SMPO using TSSA and TSID 33
B.3.3.6 Physicochemical Properties for T18.Valsartan SMARTS 35
B.3.3.7 Top 15 Generated Molecules for T18.Valsartan SMARTS 37
B.3.3.8 Top 15 Generated Molecules for T9.Median Molecules 1 Using TSMG 44
B.3.3.9 Top 10 Generated Molecules for T16.Sitagliptin MPO 45
C. More Experimental Results on Distinctive Properties 49
C.1 Distributions of Tokens on Zinc 49
C.2 Nesting Depth 50
C.2.1 Nesting Depth on ChEMBL 50
C.2.2 Nesting Depth on Zinc 51
C.2.3 Special Case on ChEMBL 52
C.3 Physicochemical Properties for Different Fragmentation Algorithms on ChEMBL 53
C.4 One Molecule and Its Reconstructed Molecules 54
D. More Experimental Results on Low-Resource Datasets 55
D.1 Figure of Inactive, Active, and Novel-Active Generated Molecules on JNK3 55
D.2 Random Reconstruction on JNK3 55
D.3 Distribution Learning on JNK3 57
D.4 Distribution Learning on JNK3 for TSDY and Hybrid Models of TSSA 58
D.5 Novelty and FCD Curves on JNK3 with TSSA 59
D.6 Physicochemical Properties on JNK3 60
D.6.1 Baseline Models on JNK3 60
D.6.2 t-SMILES Models on JNK3 61
D.7 Different Training Epochs for t-SMILES and SELFIES on JNK3 62
D.7.1 Training Data [Active Molecules] 62
D.7.2 TSSA_S[Rnd50] 62
D.7.3 TSSA_S[Rnd100] 63
D.7.4 TSSA_S[Rnd300] 63
D.7.5 TSSA_S[Rnd5000] 63
D.7.6 SELFIES[Rnd50] 64
D.7.7 SELFIES[Rnd100] 64
D.7.8 SELFIES[Rnd200] 64
D.7.9 SELFIES[Rnd1000] 65
D.8 Ablation Study on JNK3 66
D.9 Distribution Learning on AID1706: 329 Active Molecules 66
D.10 Physicochemical Properties on AID1706 67

D.11 Figure on AID1706 ... 68

E. More Experimental Results on ChEMBL, Zinc and QM9 ... 69

E.1 Random Reconstruction on ChEMBL ... 69

E.2 Random Reconstruction on Zinc ... 71

E.3 Random Reconstruction on QM9 ... 72

E.4 Physicochemical Properties on ChEMBL ... 73

E.4.1 Baseline Models on ChEMBL ... 74

E.4.2 Singleton TSSA Models on ChEMBL ... 75

E.4.3 Singleton TSDY and TSID Models on ChEMBL ... 76

E.4.4 Hybrid t-SMILES Models on ChEMBL ... 77

E.5 Physicochemical Properties on Zinc ... 78

E.5.1 Baseline Models on Zinc 1 ... 79

E.5.2 Baseline Models on Zinc 2 ... 80

E.5.3 Singleton t-SMILES Models on Zinc ... 81

E.5.4 Hybrid t-SMILES Models on Zinc ... 82

E.6 Experiments on QM9 ... 83

E.6.1 Distribution Learning on QM9 ... 83

E.6.2 Physicochemical Properties on QM9 ... 84

Supplementary Fig.63 Physicochemical properties of generated molecules on QM9. ... 84

E.6.3 Models with Higher Novelty Scores on QM9 ... 85

E.7 Experiments using LSTM and 5 Layers miniGPT ... 86

E.8 Experiments for Open-Ring on ChEMBL ... 86

E.8.1 Distribution Learning and Physicochemical Properties ... 86

E.8.2 Supplementary Discussion ... 87

F. Newly Generated Molecules ... 89

F.1.0 Molecules on Training Dataset ChEMBL (Randomly Selected) ... 89

F.1.1 Generated Molecules on ChEMBL (Randomly Selected, TSDY_HBMSV) ... 90

F.2.0 Molecules on Training Dataset Zinc (Randomly Selected) ... 91

F.2.1 Generated Molecules on Zinc (Randomly Selected) ... 92

F.3.0 Molecules on Training Dataset QM9 (Randomly Selected) ... 93

F.3.1 Generated Molecules on QM9 (Randomly Selected) ... 94

G. Atom Environment Substructure on ChEMBL ... 95

List of Abbreviations ... 101

Supplementary References ... 102

This is a supporting information (SI) file for **t-SMILES: A Fragment-based Molecular Representation Framework for De Novo Ligand Design.**

A. Algorithms

This section introduces the TSSA algorithm and provides examples of molecules with different codes. Afterwards, the BFS algorithm is demonstrated, followed by the JTVAE[1], BRICS[2], MMPA[3], and Scaffold[4] based molecule decomposition algorithms in t-SMILES procedure. Finally, we provide a brief overview of the fragmentation algorithms used in this study.

To simplify the description, in this study, a molecular graph is defined as $G = (V, E)$,where V is the set of atoms (vertices) and E is the set of bonds (edges).

A.1 TSSA Algorithm and Example Molecules

Supplementary Fig. 1 presents an overview of the TSSA procedure that uses MMPA as a fragmentation algorithm. The text also includes additional examples such as aspirin, caffeine, paracetamol and a chiral molecule. To represent molecules or fragments, it is recommended to use the Kekule style, although t-SMILES supports both styles. Therefore, these examples include both Kekule and non-Kekule styles for reference.

A.1.1 TSSA Algorithm

In Fig.1 and Supplementary Fig. 1, the three t-SMILES codes of Celecoxib are:

1) TSID_M (Fig.1):

   [1*]C&[1*]C1=CC=C([2*])C=C1&[2*]C1=CC([3*])=NN1[5*]&[3*]C([4*])(F)F&[4*]F^[5*]C1=CC=C([6*])C=C1&&[6*]S(N)(=O)=O&&&

2) TSDY_M (Fig.1, replace [n*] with *):

   *C&*C1=CC=C(*)C=C1&*C1=CC(*)=NN1*&*C(*)(F)F&*F^*C1=CC=C(*)C=C1&&*S(N)(=O)=O&&&

3) TSSA_M (Supplementary Fig. 1):

   CC&C1=CC=CC=C1&CC&C1=C[NH]N=C1&CN&C1=CC=CC=C1^CC^CS&C&N[SH](=O)=O&CF&&&&FCF&&

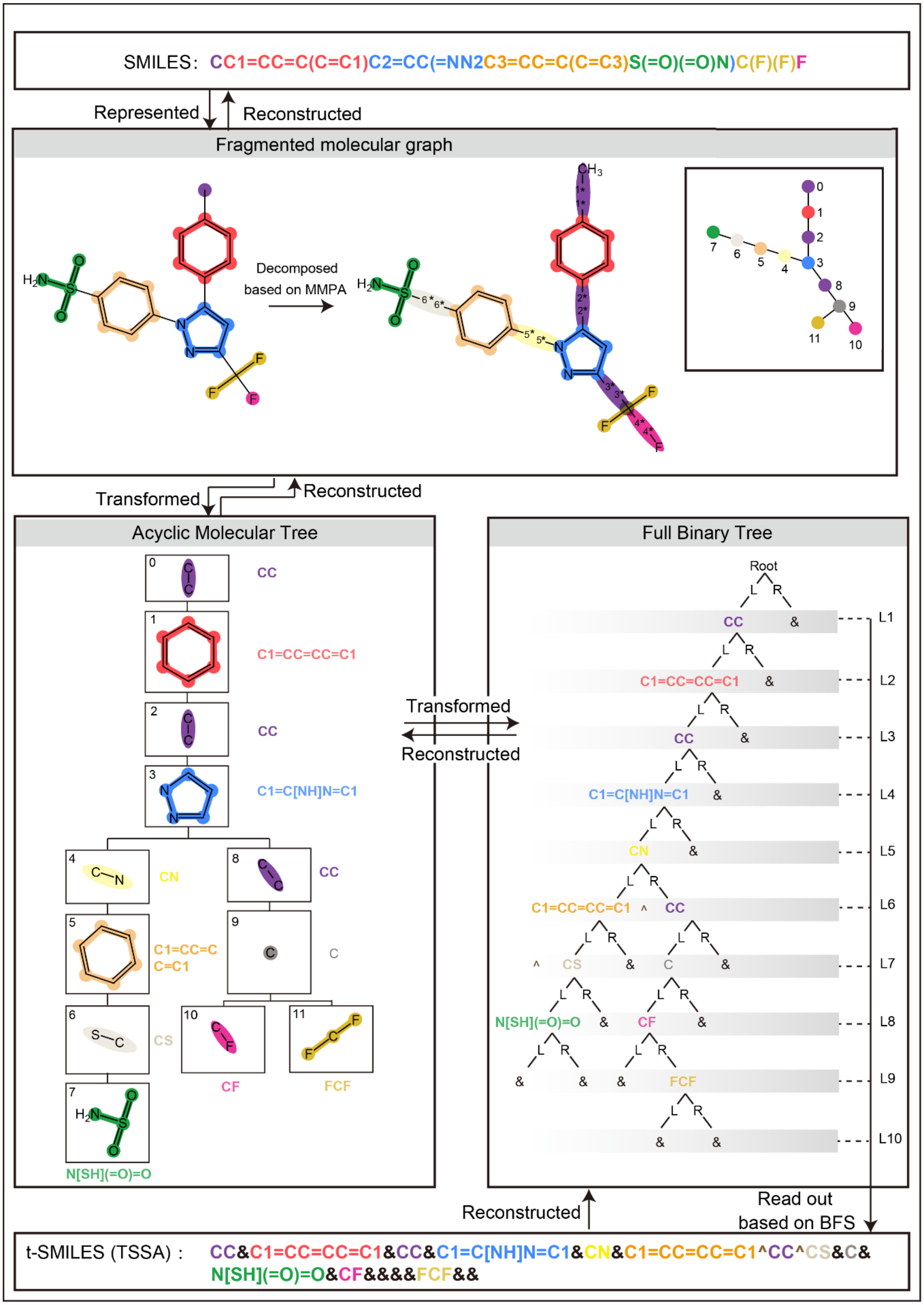

Supplementary Fig. 1 Overview of TSSA algorithm. MMPA is used as example to fragment molecules.

In FBT, molecular fragments are placed as tree nodes (left node or right node). If a tree node represents a real fragment, its SMILES is used. However, if the tree node does not represent a real fragment according to the conversion algorithm from AMT to FBT, the symbol '&' is used instead. The new symbol '^' functions similarly to the blank space in English sentences, separating two words. When generating a t-SMILES string using BFS, there are two scenarios in which the new symbol '^' is used.

1) In one level, if both the left and right nodes are SMILES, such as L6 in Supplementary Fig. 1 and L5 in Fig.1, use the symbol '^' to separate them.
2) Between two levels, if the right node of the previous level and the left node of the next level are both SMILES, such as L6 and L7 in Supplementary Fig. 1, use the symbol '^' to split them.

The key points of different code algorithms are as following:

The TSID code is the easiest one to understand, where the dummy atom and its id [1*], [2*]…[n*] are used to indicate joint points. When IDs are removed from the TSID code, the TSDY code is created, where dummy atom * without ID is used to indicate joint point in different pieces.

The fragmentation procedure used in TSSA is rooted in JTVAE[1]. JTVAE is somewhat complex and differs from BRICS[2], MMPA[3], and Scaffold[4] from a chemical point of view. To put it simply, JTVAE first cuts rings as fragments and then decomposes single atoms that are not in a ring. Single atoms that are bonded to a neighbor plus the bond between them to create a fragment. Therefore, TSSA code uses a different way to generate pieces, where a real atom is shared by two pieces. This is illustrated in Supplementary Fig. 1, which presents three cases:

Case1: In Node 0, a single atom of C is linked to its neighbor by another C, resulting in the generation of C-C as one fragment.

Case 2: The C-N bond between Node 3 and 5 is cut down, resulting in three fragments: Node 3,4 and 5 in TSSA code. Node 4 acts as a bridge fragment linking the original ones. As a result, node 4 shares a N atom with Node 3, and a C atom with Node 5.

Case 3: Node 3, Node 11 and single atom F in Node 10, are linked to a shared atom C in Node 11. The C-C bond between Node 3 and Node 11, and C-F bond between Node11 and the single atom F in Node 10 are cut down in this case. To identify this complex relation where one atom is shared

by more than two fragments, a node with a single atom, Node 9, is generated, and a bridge fragment Node 8 is generated to link Node 9 and Node 3. The logic of Node 10 is the same as in Case 1.

All fragmentation algorithms used in this study, including JTVAE[1] have all been previously published. Interested readers are recommended to refer to the original papers and to RDKit for code. For the information on how to generate AMT and convert it to FBT, we refer the interested reader to S. Radhakrishnan *et al*.[5] for a detailed algorithm and principles of graph theory. SI.A.2 demonstrates the BFS algorithm.

Different codes of Celecoxib are shown in Supplementary Table 1. RDKit(2020.03.2.0) is used to do the fragmentation.

Supplementary Table 1 Different codes of Celecoxib.

Coding Algorithm	Celecoxib	Length
SMILES	CC1=CC=C(C=C1)C2=CC(=NN2C3=CC=C(C=C3)S(=O)(=O)N)C(F)(F)F	56
DSmiles	CC=CC=CC=C6))C=CC=NN5C=CC=CC=C6))S=O)=O)N))))))))CF)F)F	55
SELFIES	[C][C][=C][C][=C][Branch1][Branch1][C][=C][Ring1][=Branch1][C][=C][C][=Branch2][Ring1][#Branch1][=N][N][Ring1][Branch1][C][=C][C][=C][Branch1][Branch1][C][=C][Ring1][=Branch1][S][=Branch1][C][=O][=Branch1][C][=O][N][C][Branch1][C][F][Branch1][C][F][F]	252
TS_Vanilla	CC1=CC=C(C=C1)C2=CC(=NN2C3=CC=C(C=C3)S(=O)(=O)N)C(F)(F)F&&&	59
TSSA-B	CC1=CC=C(C2=CC(C(F)(F)F)=NN2C2=CC=C(S(N)(=O)=O)C=C2)C=C1&&&	59
TSDY-B	CC1=CC=C(C2=CC(C(F)(F)F)=NN2C2=CC=C(S(N)(=O)=O)C=C2)C=C1&&&	59
TSID-B	CC1=CC=C(C2=CC(C(F)(F)F)=NN2C2=CC=C(S(N)(=O)=O)C=C2)C=C1&&&	59
TSSA-M	CC&C1=CC=CC=C1&CC&C1=C[NH]N=C1&CN&C1=CC=CC=C1^CC^CS&C&N[SH](=O)=O&CF&&&&FCF&&	77
TSDY-M	*C&*C1=CC=C(*)C=C1&*C1=CC(*)=NN1*&*C(*)(F)F&*F^*C1=CC=C(*)C=C1&&*S(N)(=O)=O&&&	78
TSID_M	[1*]C&[1*]C1=CC=C([2*])C=C1&[2*]C1=CC([3*])=NN1[5*]&[3*]C([4*])(F)F&[4*]F^[5*]C1=CC=C([6*])C=C1&&[6*]S(N)(=O)=O&&&	114
TSSA-S	CC&C1=CC=C(C2=CC=NN2C2=CC=CC=C2)C=C1&CC&FC(F)F^CS&&N[SH](=O)=O&&&	65
TSDY-S	*C&*C1=CC=C(C2=CC(*)=NN2C2=CC=C(*)C=C2)C=C1&*C(F)(F)F&&*S(N)(=O)=O&&	68
TSID-S	[1*]C&[1*]C1=CC=C(C2=CC([2*])=NN2C2=CC=C([3*])C=C2)C=C1&[2*]C(F)(F)F&&[3*]S(N)(=O)=O&&	86
TSSA-J	CC&C1=CC=CC=C1&CC&C1=C[NH]N=C1&CC&C^CN^CF&C1=CC=CC=C1&&CF^CS&&CF^S&&&NS&&O=S&O=S&&	82

A.1.2 Aspirin

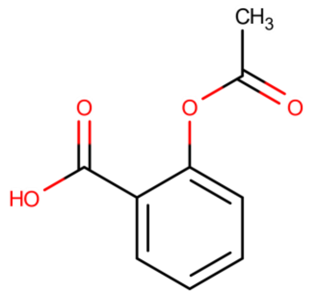

Supplementary Fig.2 Structural formula of molecule Aspirin

Supplementary Table 2 Different codes of Aspirin.

Coding Algorithm	Aspirin	Length
SMILES	CC(=O)OC1=CC=CC=C1C(=O)O	24
DeepSmiles	CC=O)OC=CC=CC=C6C=O)O	21
SELFIES	[C][C][=Branch1][C][=O][O][C][=C][C][=C][C][=C][Ring1][=Branch1][C][=Branch1][C][=O][O]	87
TS_Vanilla	CC(=O)OC1=CC=CC=C1C(=O)O&&&	27
TSSA-B	CC=O&CO&O=C(O)C1=CC=CC=C1O&&&	29
TSDY-B	*C(C)=O&*Oc1ccccc1C(=O)O&&&	27
TSID-B	[1*]C(C)=O&[1*]Oc1ccccc1C(=O)O&&&	33
TSSA-M	CC&O=CO&CO&C1=CC=CC=C1&CC&O=CO&&&	33
TSDY-M	*C&*C(=O)O*&*C1=CC=CC=C1*&*C(=O)O&&&	36
TSID-M	[1*]C&[1*]C(=O)O[2*]&[2*]C1=CC=CC=C1[3*]&[3*]C(=O)O&&&	54
TSSA-S	CC(=O)O&CO&C1=CC=CC=C1&CC&O=CO&&&	33
TSDY-S	*OC(C)=O&*C1=CC=CC=C1*&*C(=O)O&&&	33
TSID-S	[1*]OC(C)=O&[1*]C1=CC=CC=C1[2*]&[2*]C(=O)O&&&	45
TSSA-J	CC&C&C=O&&CO^CO&C1=CC=CC=C1&CC&C&C=O&&CO&&	42

A.1.3 Caffeine

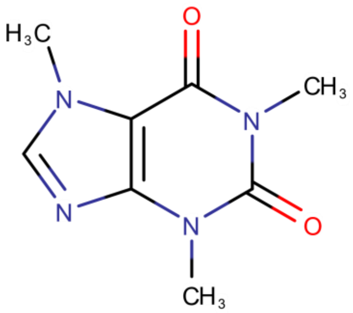

Supplementary Fig.3 Structural formula of molecule Caffeine

Supplementary Table 3 Different codes of Caffeine.

Coding Algorithm	Caffeine	Length
SMILES	CN1C=NC2=C1C(=O)N(C(=O)N2C)C	28
DeepSmiles	CNC=NC=C5C=O)NC=O)N6C)))C	25
SELFIES	[C][N][C][=N][C][=C][Ring1][Branch1][C][=Branch1][C][=O][N][Branch1][=Branch2][C][=Branch1][C][=O][N][Ring1][Branch2][C][C]	123
TS_Vanilla	CN1C=NC2=C1C(=O)N(C(=O)N2C)C&&&	31
TSSA-B	CN1C(=O)C2=C(N=CN2C)N(C)C1=O&&&	31
TSDY-B	CN1C(=O)C2=C(N=CN2C)N(C)C1=O&&&	31
TSID-B	CN1C(=O)C2=C(N=CN2C)N(C)C1=O&&&	31
TSSA-M	CN&O=C1[NH]C(=O)C2=C(N=C[NH]2)[NH]1&CN&&CN&&	44
TSDY-M	*C&*n1c(=O)c2c(ncn2*)n(*)c1=O&*C&&*C&&	38
TSID-M	[1*]C&[1*]n1c(=O)c2c(ncn2[2*])n([3*])c1=O&[2*]C&&[3*]C&&	56
TSSA-S	CN&O=C1[NH]C(=O)C2=C(N=C[NH]2)[NH]1&CN&&CN&&	44
TSDY-S	*C&*n1c(=O)c2c(ncn2*)n(*)c1=O&*C&&*C&&	38
TSID-S	[1*]C&[1*]n1c(=O)c2c(ncn2[2*])n([3*])c1=O&[2*]C&&[3*]C&&	56
TSSA-J	CN&C1=C[NH]C=N1&C1=CNCNC1&C=O&&C=O&CN&CN&&	42

A.1.4 Paracetamol

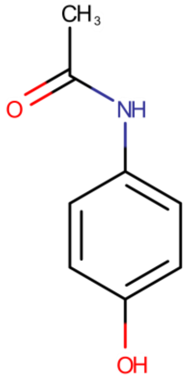

Supplementary Fig.4 Structural formula of molecule Paracetamol

Supplementary Table 4 Different codes of Paracetamol.

Coding Algorithm	Paracetamol	Length
SMILES	CC(=O)NC1=CC=C(C=C1)O	21
DeepSmiles	CC=O)NC=CC=CC=C6))O	19
SELFIES	[C][C][=Branch1][C][=O][N][C][=C][C][=C][Branch1][Branch1][C][=C][Ring1][=Branch1][O]	85
TS_Vanilla	CC(=O)NC1=CC=C(O)C=C1&&&	24
TSSA-B	CC=O&CN&NC1=CC=C(O)C=C1&&&	26
TSDY-B	*C(C)=O&*Nc1ccc(O)cc1&&&	24
TSID-B	[1*]C(C)=O&[1*]Nc1ccc(O)cc1&&&	30
TSSA-M	CC&NC=O&CN&C1=CC=CC=C1&CO&&&	28
TSDY-M	*C&*C(=O)N*&*C1=CC=C(*)C=C1&*O&&&	33
TSID-M	[1*]C&[1*]C(=O)N[2*]&[2*]C1=CC=C([3*])C=C1&[3*]O&&&	51
TSSA-S	CC(N)=O&CN&C1=CC=CC=C1&CO&&&	28
TSDY-S	*NC(C)=O&*C1=CC=C(*)C=C1&*O&&&	30
TSID-S	[1*]NC(C)=O&[1*]C1=CC=C([2*])C=C1&[2*]O&&&	42
TSSA-J	CC&C&C=O&&CN^CN&C1=CC=CC=C1&CO&&&	33

A.1.5 Molecule with Chirality

It is straightforward to list possible stereoisomers for a molecule using RDKit, but t-SMILES supports both styles, so this example includes fragments with or without chirality for reference.

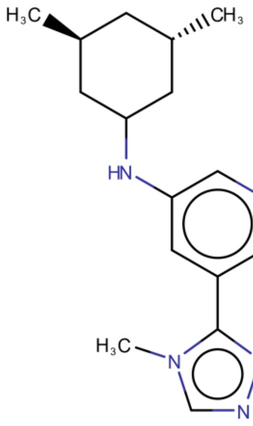

Supplementary Fig 5 Structural formula of molecule with Chirality

Supplementary Table 5 Different codes of Molecule with Chirality.

Coding Algorithm	Molecule with Chirality	Length
SMILES	C[C@@H]1CC(Nc2cncc(-c3nncn3C)c2)C[C@@H](C)C1	44
DeepSmiles	C[C@@H]CCNccncc-cnncn5C))))))c6)))))))C[C@@H]C)C6	49
SELFIES	[C][C@@H1][C][C][Branch2][Ring1][Ring2][N][C][=C][N][=C][C][Branch1][=Branch2][C][=N][N][=C][N][Ring1][Branch1][C][=C][Ring1][N][C][C@@H1][Branch1][C][C][C][Ring2][Ring1][Ring2]	177
TS_Vanilla	C[C@@H]1CC(NC2=CN=CC(C3=NN=CN3C)=C2)C[C@@H](C)C1&&&	51
TSSA-B	CC1CCCC(C)C1&CN&CN1C=NN=C1C1=CC(N)=CN=C1&&&	43
TSDY-B	*C1C[C@@H](C)C[C@H](C)C1&*NC1=CN=CC(C2=NN=CN2C)=C1&&&	53
TSID-B	[1*]C1C[C@@H](C)C[C@H](C)C1&[1*]NC1=CN=CC(C2=NN=CN2C)=C1&&&	59
TSSA-M	CC&CC1CCCCC1&CN&CN&C1=CC=NC=C1&CC&C1=NN=C[NH]1&CN&&&	52
TSDY-M	*C&*[C@@H]1CC(*)C[C@@H](C)C1&*N*&*C1=CN=CC(*)=C1&*C1=NN=CN1*&*C&&&	66
TSID_M	[1*]C&[1*][C@@H]1CC([2*])C[C@@H](C)C1&[2*]N[3*]&[3*]C1=CN=CC([4*])=C1&[4*]C1=NN=CN1[5*]&[5*]C&&&	96
TSSA-S	CC&C1=NN=C(C2=CC(NC3CCCCC3)=CN=C2)[NH]1&CN&&CC&&	48
TSDY-S	*C&*[C@@H]1CC(NC2=CN=CC(C3=NN=CN3*)=C2)C[C@@H](*)C1&*C&&*C&&	60
TSID-S	[1*]C&[1*][C@@H]1CC(NC2=CN=CC(C3=NN=CN3[2*])=C2)C[C@@H]([3*])C1&[2*]C&&[3*]C&&	78
TSSA-J	CC&C1CCCCC1&CN&CN^CC^C1=CC=NC=C1&&&CC&C1=NN=C[NH]1&CN&&&	56

A.2 Algorithm 1: BFS

Breadth first traversal for the tree is level order traversal of a tree. We first traverse a node. Then, we traverse all the neighbor nodes in the present depth prior to moving on to the nodes in the next level of the tree and thus named level order traversal.

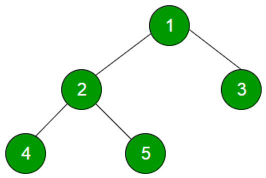

Supplementary Fig.6 BFS Algorithm.

Level order traversal of the above tree is: 1 2 3 4 5

A.3 Algorithm 2: JTVAE Fragmentation Algorithm

One molecular decomposition algorithm in our study could find its root in feature tree[6] published for molecular similarity algorithm by Rarey *et al*. in 1998 and JTVAE[1] proposed later by Jin *et al.* Given a molecular graph G, we firstly find all simple rings as single cluster whose edges do not belong to any other rings.

1) Two simple rings are merged as one cluster if they share more than two overlapping atoms, as they constitute a specific structure called bridged compounds[7].
2) For the acyclic part of the molecule, each atom with more than one bonds forms a separate node in the acyclic molecular tree.
3) The remaining terminal atoms form a single node with the atom they are connected to.
4) Edges are added between nodes containing atoms that are connected in the original molecular graph.
5) Finally, we select one of its spanning trees as the acyclic molecular tree of G.

A.4 Algorithm 3: BRICS, MMPA and Scaffold Fragmentation Algorithms in t-SMILES

We cut-off molecular graph according to the principles of BRICS[2], MMPA[3], and Scaffold[4], which are implemented in open-source molecular toolkit RDKit, and then calculate its spanning tree as AMT.

1) Given a molecular graph G, we firstly break bonds based on BRICS, MMPA and Scaffold algorithm
2) Get molecular fragments using TSSA, TSDY, or TSID logic.
3) Each fragment with atoms and bonds forms a single node.
4) Edges are added between nodes containing atoms that are connected in the original molecular graph.
5) Finally, we select one of its spanning trees as the acyclic molecular tree of G.

A.5 Brief Overview of Related Fragmentation Algorithms

In his 1988 Nobel Lecture[8], Lehn refined his definition of supramolecular chemistry. He has since drawn parallels between language and chemistry, famously stating that atoms are letters, molecules are words, and supramolecular entities are sentences and chapters[9]. Cadeddu et al.[10] later analyzed the rank distribution of fragments in organic molecules and found it to be similar to that of words in the English language. Due to the similarities between natural languages and molecules, techniques derived from natural language processing (NLP) have been successfully applied to solve chemical problems, such as drug discovery tasks.

When borrowing advanced NLP methodologies to address chemical problems, the first question that arises is: What are 'chemical words'?

In this study, molecular fragments are compared to “chemical words”. Similar to word segmentation algorithms in NLP, fragmenting molecules is a significant challenge task. Fortunately, there are some published algorithms, such as JTVAE[1], BRICS[2], MMPA[3] and Scaffolds[4], available that can generate chemical fragments. The in-depth study of this topic is beyond the scope of this paper. Below is a brief overview of these algorithms used in this study.

The study of fragmentation methodologies and their applications continues to reveal new opportunities for efficient molecular design and development. A review[11] paper summarized a total of 15 published algorithms, such as eMolFrag[12] etc. Interested readers are encouraged to refer to summary and the original paper.

BRICS(Breaking of Retrosynthetically Interesting Chemical Substructures) BRICS[2] algorithm disconnect a molecule to fragments on 16 bond types based on chemical reaction rules.

The RDKit API provides two options for invoking it: BRICS.FindBRICSBonds() and BRICS.BRICSDecompose().

The BRICS[2] method is based on the RECAP[13] approach, which cleaves molecules at 11 chemical bond types that correspond to common chemical reactions[13]. The cleaving process is exhaustive, meaning no intermediate structures are generated. Simple functional groups, such as hydrogen, methyl, ethyl, propyl, and butyl, are not removed. Additionally, only acyclic bonds are considered, leaving ring structures intact. The authors suggest that identifying ring motifs in this manner is easier and can be related to a chemist's perception of ring structures. Additionally, rings are often left un-cleaved because they are a valuable topological feature that contributes to the scaffold of a molecule. When a molecule is cleaved, the fragments are assigned connection points to preserve information about compatible reactions.

Although BRICS and RECAP are similar, BRICS aims to improve the current approach for molecule fragmentation by utilizing a more comprehensive set of 16 rules and additional pre- and post-filters. In addition to the RECAP approach, the selection of bonds to be cut also considered the adjacent substructure to a particular bond type. This consideration of the bond's environment can prevent the formation of undesired chemical motifs, such as small terminal fragments that include hydrogen and halogen atoms, or functional groups like hydroxy, nitro, carboxylate, methoxy, methyl, ethyl, and isopropyl groups.

In this study, considering that the training data are mainly small molecules, and the molecular fragments segmented by BRICS are large or molecules could not be broken down, if necessary, we could further cut off the branch structures connected to ring structures on the basis of BRICS(BRICS_Adv), and then cut off the bridge bonds between two rings.

MMPA(Matched Molecular Pair Analysis) As described by Hussain and Rea[3], a Molecular Matched Pair (MMP) is a pair of compounds (compound A and compound B) that only differ by a single localized structural change. The RDKit API invokes the MMPA algorithm as rdMMPA.FragmentMol().

Matched molecular pairs (MMPs) analysis was first suggested by Leach and colleagues[14]. The compounds belonging to an MMP can be converted to one another by the molecular transformation of substructure A to substructure B, where substructures A and B are the substructures that have changed from compound A to compound B. MMPA has been utilized to predict the impact of

substituting one group for another on various compound properties[15], and has become a major tool for analyzing large chemistry dataset for promising chemical transformations[14,16].

Scaffold In general terms, a scaffold is a molecular core to which functional groups are attached[4], as defined by the RDKit API's MurckoScaffold.GetScaffoldForMol() algorithm.

Given a chemical compound, its Scaffold generally represents the core structures of the molecular framework. In real-world molecular design, a common strategy is first identifying initial candidates and then modifying their side chains while maintaining their Scaffolds. This strategy is particularly effective in designing protein inhibitors because the structural arrangement between a scaffold and protein residues is a main source of protein-ligand binding. Despite its importance, such scaffold-based molecular design has drawn surprisingly less attention in developing molecular generative models[17].

B. Experiments

This section begins with a summary of t-SMILES, followed by a brief introduction to the experiments used in this study. Next, we provide an overview of the datasets and three types of evaluation metrics.

From systemically experiments and nuanced discussions, it is evident that TSSA, TSDY, and TSID have different distinct advantages at different points. They complement each other well but cannot be completely replaced by one another. Supplementary Table 6 presents a preliminary summary of three codes of t-SMILES: TSSA, TSDY, and TSID.

Supplementary Table 6 Summary of t-SMILES.

Code	Joint Point	Frag Alg.	Experiments	Relative Pros and Cons of TSSA, TSDY and TSID
TSSA	Shared Atom	JTVAE,<br>BRICS,<br>MMPA,<br>Scaffold,<br>Hybrid	ChEMBL<br>Zinc<br>QM9<br>Low-Resource<br>Goal-Oriented<br>GPT, LSTM<br>Transfer Learning<br>Data Augmented	1. Highest reconstruction novelty score;<br>2. Generative model without training;<br>3. In-depth investigation is encouraged to verify whether TSSA code algorithm could benefit from its distinctive structure, as some pieces are bonds that are broken.
TSDY	Dummy Atom, without ID	BRICS,<br>MMPA,<br>Scaffold,<br>Hybrid	ChEMBL<br>Zinc<br>QM9<br>Low-Resource<br>Goal-Oriented<br>GPT, LSTM	1. Medium reconstruction novelty score;<br>2. Generative model without training;<br>3. High computational performance on reconstruction: less candidates, faster;<br>4. Better fitting training data on physicochemical properties;<br>5. Better performance on larger molecules;
TSID	Dummy Atom with ID	BRICS,<br>MMPA,<br>Scaffold,<br>Open-Ring,<br>Hybrid	ChEMBL<br>Zinc<br>QM9<br>Goal-Oriented<br>Open-Ring<br>GPT, LSTM	1. Almost zero reconstruction novelty score;<br>2. Need to be trained to build generative model;<br>3. High computational performance on reconstruction: less candidates, faster;<br>4. Better fitting training data on physicochemical properties;<br>5. Better performance on larger molecules.<br>6. Longest;
TS_Vanilla			ChEMBL<br>Zinc<br>QM9<br>Low-Resource<br>Goal-Oriented<br>GPT, LSTM	1. Classical SMILS in t-SMILES format.

For general purposes, if simply categorize tasks into two types: goal-oriented and distribution reproduction, different algorithms could be simply proposed for each type of task as:

1. For goal-oriented tasks, the preferred option is TSSA_S to avoid any 'striking similarity' to the training dataset and achieve 'better novelty with reasonable similarity'.
2. For distributional reproduction tasks, it is preferred to use TSID with a hybrid fragmentation scheme to explore chemical spaces as much as possible and achieve high similarity to the training dataset.
3. Different TSDY models yield balanced scores, so it would be better to choose based on chemical purpose. If to be simple, TSDY_M is proposed as the optimal choice for balanced goal-oriented and distribution reproduction tasks.

Furthermore, molecular fragmentation schemes are based on specific chemical principles. From this perspective, it is not easy to make a definitive judgment about which option is superior to another. In addition, in real-world molecular design experiments, the goal is often to address a specific problem, such as designing a molecule with a particular scaffold. Similarly, chemists may wish to perform a thorough investigation of open ring problems. In these scenarios, different fragmentation schemes should be selected to accomplish the given task.

Chemists often face challenging problems, and their potential choices go far beyond the scope of systematic exploration. The t-SMILES framework allows for the design of models using multi-code combination strategies or multi-model systems with different fragmentation schemes. As demonstrated in 'Chemical Space' and 'Goal-Directed Learning', t-SMILES-based models can comprehensively explore the complexity and diversity of chemical space. In addition to the aforementioned proposal, users can also design their own t-SMILES models based on the empirical results of this study to achieve specific experimental goals.

B.1 Generation Algorithms and Baseline Models

Language modelling uses probability and statistical techniques to calculate the possibility of word sequences in sentences and then do estimation[18]. Originally, recurrent neural network (RNN) is designed to address this kind of sequence problems. To address the limitation of RNN that lead to gradient disappearance and explosion for long sequences, cyclic models such as long short-term memory (LSTM)[19] and Gated Recurrent Unit (GRU)[20] are proposed. However, it has been shown that the power of LSTMs is insufficient when the information has ultra-long dependencies. In 2017, the attention-based Transformers[21] architecture broke through the limitations of LSTM and quickly achieved SOTA scores on multiple metrics in NLP and computer vision. After that, the improved

models such as GPT1-3[22,23,24] and BERT[25] are introduced to build pre-trained model on large datasets. Although these large-size models have achieved unprecedented performances, they come at high computational costs. Consequently, some of the recent NLP architectures have utilized concepts of transfer learning, pruning, quantization, and knowledge distillation to achieve moderate model sizes while keeping nearly similar performances as achieved by their predecessors. The recent developments in the NLP field have a great potential to be adapted to molecular de novo generation research. Bagal *et al.*[26] has proposed a Transformer-decoder model with 10 layers to molecular generation task and proved it works well.

B.1.1 Setting and Baseline Models

In this study, we mainly adopt transformer-decoder based autoregressive generation models to evaluate our proposed t-SMILES. As the training data is more complex than the baseline model MolGPT[26] and the batch size of 384 is too large for our available computing resources, for the sake of data comparability, we opted to retrain it using the AdamW optimizer, with a learning rate of 0.001 and batch size of 128 for t-SMILES and classical SMILES, DeepSMILES, SELFIES, transfer learning, and data augmentation based models. Another miniGPT model is also trained for evaluation, which is a 5 layers mini version of GPT2 with 256 hidden layers and 8 attention headers.

We retrain JTVAE[1] on Zinc using the publicly available codebase provided by the paper's authors and then generate 20K molecules.

We calculate metrics based on the molecules provided by Fragment-base-DGM[27] on Zinc.

We generate 20K molecules using the pre-trained model provided by hgraph2graph[28] (hG2G) on ChEMBL and calculate metrics for evaluation.

We retrain CReM[29] for 20 Goal-Directed Learning tasks on ChEMBL.

We train LSTM modes using SMILES, DeepSMILES, SELFIES and t-SMILES on ChEMBL for Goal-Directed Learning tasks.

We do not train the rest of the baseline models by ourselves. For QM9 and ChEMBL, we take the results from Bagal *et al.*[26], Cao and Kipf[30], O. Mahmood *et al.*[31].

B.1.2 Training and Repeatability

Generally speaking, training data, input or description, model type (LSTM or GPT etc.) and hyperparameters are some key factors that affect the performance. If possible, it would be better to train and optimize each type of model independently and select the best hyperparameters to perform the analysis. To be fair and comparable, there are some simple principles and statistics in our experiments.

We fix the model's architecture and most of the hyperparameters, and mainly update the training epochs to get different evaluation metrics.

Also, since SELFIES tokenizes sequence like [#Branch1], [#C-1], [Ring2], etc., not character by character, we do not yet tokenize SMILES, DeepSMILES, and t-SMILES character by character, but groups of characters like [C+], [NH+], etc.

Furthermore, it is important to recognize the potential impact of experimental errors on performance due to training, randomness, etc.

Therefore, we train each model from scratch on labeled and low-resource data, JNK3, with different epochs, such as R50, R100, R200, R300, R500, R1000, R2000, R5000, and R50000. Since each model is trained from zero, this series could somehow cover random errors. It's important to note that the data shows a significant negative correlation between novelty and FCD values. This is a key point for further experiments on other training data.

The systemic experiments then move from the complex and large molecules of ChEMBL to the small and simple molecules of Zinc and QM9.

On ChEMBL, because baseline model MolGPT[26] has been well trained, more than five SMILES-based models are first trained on our environment with different hyperparameters to obtain a relatively best model, compared to this key baseline model.

And then DeepSMILES, SELFIES, TS-Vanilla models are trained from scratch with different training epochs and check if the novelty-FCD scores are negatively correlated. If it's true, then we assume that the system errors are in a limited and acceptable range. If there are some special values, we retrain this model from zero and select a best result.

For other t-SMILES models, as we train some groups, such as (TSDY_M, TSDY_HMV, TSDY_HBMSV), (TSID_B, TSID_HBV, TSDY_HBMSV), so it is possible to somehow cover

part of random error. And the statistics shows that all tested t-SMILES models get higher novelty scores than SMILES model. In addition, see SI.Table.B.1.2 for the Mean and StdDev values of the TS-Vanilla, TSDY, and TSID models. It indicates that the errors of the FCD scores are in the acceptable range.

TSSA models are not included in Supplementary Table 7 because TSSA models have lower performance than TSDY and TSID, with appropriately lower scores.

Molecules on Zinc are much smaller and simpler than ChEMBL, so they receive correspondingly lower StdDev scores. The particularly high StdDev score of 0.091 on QM9 is because we intentionally train some special models with higher novelty scores.

Supplementary Table 7 The Mean and StdDev of TS-Vanilla, TSDY, and TDID models.

		**Novelty**	**FCD**
ChEMBL<br>14 Modles	Mean	0.945	0.887
	StdDev	0.017	0.018
Zinc<br>10 Modles	Mean	0.981	0.926
	StdDev	0.008	0.007
QM9<br>7 Modles	Mean	0.252	0.977
	StdDev	0.091	0.005

The t-SMILES model TSID_S_[R10] achieves the highest FCD score of 0.891 among t-SMILES models. We evaluate its repeatability by running the model three times. A summary of metrics can be found in Supplementary Table 8.

Supplementary Table 8 Results for TSID_S models on ChEMBL using GPT.

**Model**	**Valid**	**Unique**	**Novelty**	**KLD**	**FCD**	**Nov./Uni.**
TSID_S_[R10]_0	1.000	0.999	0.933	0.991	0.909	0.935
TSID_S_[R10]_1	1.000	0.998	0.935	0.993	0.909	0.936
TSID_S_[R10]_2	1.000	0.999	0.936	0.993	0.907	0.937
Mean	1.000	0.999	**0.935**	0.992	0.908	0.936
StdDev	0.000	0.000	0.001	0.001	0.001	0.001

In this experiment, the SMILES model scores (0.907, 0.906) for Novelty and FCD respectively in Table3. According to Supplementary Table 8, the system error is acceptable, as shown by the StdDev values. This indicates that the t-SMILES model is stable in achieving higher Novelty and FCD scores. Additionally, the novelty score outperforms the SMILES model significantly.

In this study, two GPUs: NVIDIA GeForce RTX 3090 and NVIDIA Quadro RTX4000 are used to train deep learning models. The encoding and decoding of t-SMILES string can be performed

on a CPU without the need for a GPU. To improve performance, future studies could explore the use of parallel techniques or other programming languages, such as C++, which are faster than Python.

Due to the similarity between t-SMILES and SMILES strings, training time is not a key indicator to evaluate their differences. For instance on GPU: NVIDIA Quadro RTX4000, CPU: Intel(R) Xeon(R) W-2265, a classical SMILES-based model takes almost 24 hours to train, while the TSID_S model takes almost 19 hours with the same training parameters: epochs = 10, batch_size = 128, and tokens such as B, Br, C, Cl, F, I, N, O, P, S, [Cl+2], [Cl+3], etc.

The cost of training depends on various factors such as hyperparameters and tokenization method. It is important to note that the reference run time is not an indication that the t-SMILES model requires less time than the SMILES model, but rather serves as a reference point.

When generating 1000 molecules on an GPU: NVIDIA Quadro RTX4000 with a batch size of 128, the TSID_S model takes approximately 40 seconds, resulting in roughly 25 molecules per second. While, the SMILES model takes around 20 seconds and produces approximately 50 molecules per second. The difference is mainly because the TSID_S and SMILES models have different token dictionaries.

One key difference between t-SMILES and SMILES is that the t-SMILES model must reconstruct the string into molecules, which distinguishes it from the SMILES-based model. The cost of reconstructing 1000 molecules is shown in Supplementary Table 9.

Supplementary Table 9 Cost to Reconstruct Molecules. The term 'candidate' refers to the number of sub-fragments selected for the next step during reconstruction.

Code	TSSA_J	TSSA_B	TSSA_M	TSSA_S	TSDY_B	TSDY_M	TSDY_S	TSID_B	TSID_M	TSID_S
s/1000Mols Candidate=1	74	22	48	172	18	47	24	17	43	22
s/1000Mols Candidate=3	133	31	150	356	27	120	43	17	43	21
Molecule/s Candidate=1	14	45	21	6	56	21	42	59	23	45
Molecule/s Candidate=3	8	32	7	3	37	8	23	59	23	48

B.2 Datasets

We use the publicly available active compounds against two targets, JNK3[32] and AID1706[33], to evaluate low-resource problems. We also evaluate t-SMILES on three commonly used public data sets, including the subset of ChEMBL-21[34], Zinc[35], and QM9[36].

The JNK3 dataset contains 923 active molecules, while the AID1706 dataset contains 329 active molecules. These data can be retrieved from the GitHub repositories:

https://github.com/jkwang93/ChemistGA

https://github.com/yangkevin2/coronavirus_data.

For the sake of universality, we select a subset of ChEMBL-21 with SMILES character length less than 120. Apart from this very simple rule, no other preprocessing is done. Molecules in ChEMBL subset are composed of 11 atoms including B, Br, C, Cl, F, H, I, N, O, P and S.

The Zinc subset used in our experiments is the same as the subset in JTVAE which contains approximately 250K drug-like molecules extracted from the Zinc15 database covering a wide range of chemical space. The molecules in this Zinc subset are composed of 10 atoms including Br，C，Cl，F，H，I，N，O，P and S.

QM9 dataset contains up to 9 heavy (non-hydrogen) atoms. Molecules in the subset consist of 5 atoms, including C, F, N, H and O. In total, this results in about 134k druglike organic molecules.

The complexity of ChEMBL and Zinc used in this study exceeds that of the GuacaMol[37] and MOSES[38] benchmarks because small molecules can be fragments of larger molecules.

Supplementary Table 10 Some basic statistics of standard data set: QM9, Zinc and Chembl used in this study

Dataset	Number of Samples	N_Characters		N_Atoms		N_Rings	
		Max	Mean	Max	Mean	Max	Mean
**Zinc**	**249454**	120	44.4	38	23.2	9	2.8
**QM9**	**133017**	37	16	9	8.8	8	1.7
**ChEMBL**	**1570407**	120	48	111	28	30	3.4

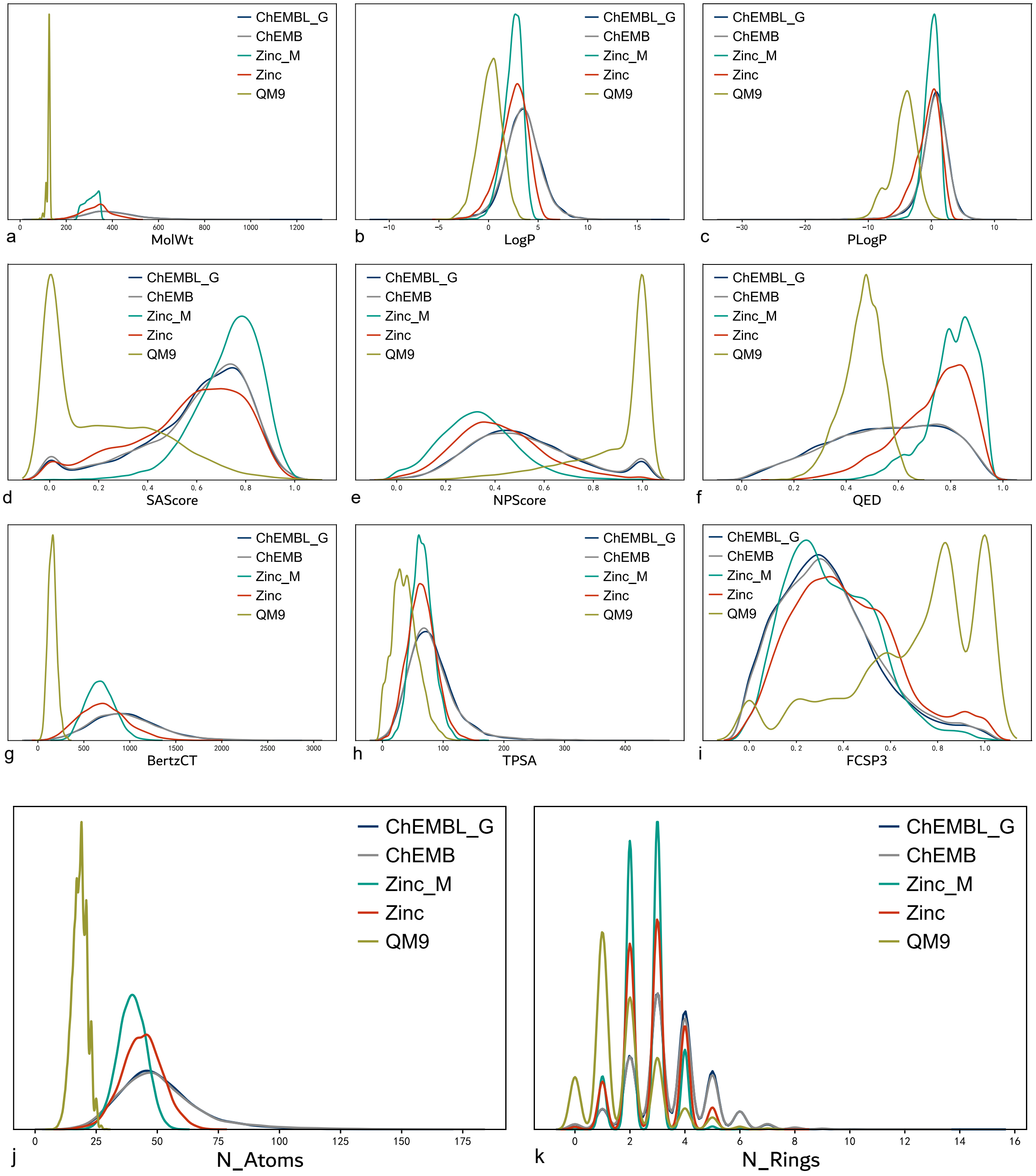

Supplementary Fig.7 Physiochemical properties of randomly selected 10K molecules from ChEMB, Zinc and QM9. The unit of the y-axis is 'Density'. The figures contain labels ['#0', '#1', '#2', '#3', '#4'] corresponding to the following datasets: ['ChEMBL_Guacamol', 'ChEMB', 'Zinc_MOSES', 'Zinc', 'QM9'].

Figures show that the properties of QM9 are relatively more fundamental and easier to be fitted. Given the complexity, the SAScore might be a better metric to evaluate the performance of models,

because no matter how nice the calculated score reached would be in vain if the generated molecular cannot be synthesized.

B.3 Benchmarks

Three types of benchmarks are used in our experiments to evaluate t-SMILES and baselines from different perspectives: distribution learning, goal-directed leaning, and physicochemical properties.

B.3.1 Standard Distribution-Learning Benchmarks

Distribution Learning assessed the ability of the generator to match the reference chemical space distribution in newly generated compounds. Despite a large number of metrics have been proposed for the evaluation of generative models of molecules, there is little consensus on which ones should be used. It is often biased to use simple indexes to evaluate the performance of different models[39]. From the perspective of optimization, it could be considered that the task can be solved once the molecules with a high score of a certain index are generated, but these generated molecules may not be useful. Therefore, we use five benchmarks proposed by GuacaMol: validity (↑), uniqueness (↑), novelty (↑), Kullback–Leibler divergence (KLD) (↑)[40] and Fréchet ChemNet Distance(FCD)[41] score (↑) to evaluate the general performance of the model. GuacaMol calculates these scores using 10K randomly sampled molecules.

Validity measures the ratio of valid molecules which could be correctly parsed by RDKit[42];

Uniqueness is the percentage of valid molecules that are unique;

Novelty is the percentage of valid and unique molecules that are not included in the training data set. This score is lower than GuacaMol's, so in some tables Nov./Uni. is calculated as a reference if there are baseline modes that we have not retrained;

$$novelty\ in\ this\ study = \frac{valid\ and\ unique\ molecules\ not\ in\ training\ set}{10K\ molecules(including\ duplicated)}$$

$$novelty\ in\ GuacaMol = \frac{valid\ and\ unique\ molecules\ not\ in\ training\ set}{10K\ unique\ molecules}$$

$$novelty\ in\ this\ study < \ novelty\ in\ GuacaMol$$

KLD score compares the distribution of a variety physicochemical descriptors of the training set and generated molecules.

FCD score measures the proximity of the distribution of generated molecules to the distribution of the dataset molecules according to the Fréchet Distance in the hidden representation space of ChemNet[43], which is trained to predict the chemical properties of small molecules.

The values of these five parameters are between 0 and 1. The larger the value, the 'better' the model. While KLD and FCD are both measure of the similarity between generated molecules and molecules from the training data, they are highly correlated with each other and inversely correlated with novelty.

In general, we randomly select 10K molecules from the generated molecules, compute five indicators based on the open source implementation of GuacaMol, and compute the distribution of physical and chemical properties. To be noted that, molecules are randomly selected from both the training dataset and the generated group. So, the results of these metrics may differ slightly every time. Small system errors are a result of random sampling. Please refer Supplementary Table 28 for the Mean and StdDev of Wasserstein distance metrics for three samplings on ChEMBL.

B.3.2 Physicochemical Properties

In addition, we use MolWt, logP, Penalized logP(plogP)[44], SA score(SAS)[45], BertzCT[46], QED[47], TPSA, NP Score(NPS)[48], FractionCSP3, N-Atoms, and N-Rings to evaluate whether the generative models could effectively learn the physical and chemical properties of the molecules in the training set, thereby comprehensively evaluating the performance of the generative model from the perspective of distributed learning knowledge.

We plot KDE's for molecular properties from all models using the same bandwidth parameter. As a **rough** reference to quantitatively evaluate the ability of each model to learn its training distribution, we compute the Wasserstein distance between the property values of the generated molecules and the training molecules. It is noteworthy that the Wasserstein distance is intended for rough reference only. The curve of the KDE should be the primary focus when analyzing the performance of distributed learning on properties.

Wasserstein distances, also known as the earth mover's distance, are computed using SciPy[49] as scipy.stats.wasserstein_distance(), it can be viewed as the minimum amount of distribution weight

that must be moved, multiplied by the distance—in order to transform samples from one distribution into samples from the another.

Molecules are processed and relevant properties are computed using RDKit (Version 2020.03.2.0).

1. **MolWt**: The molecular weight of the molecule.
2. **logP**: The logarithm of the partition coefficient. If one of the solvents is water and the other is a nonpolar solvent, then logP is a measure of hydrophobicity.
3. **Penalized logP(plogP)**[44] is the logarithm of the partition ratio of solute between octanol and water subtracted by synthetic accessibility score and long cycles. It has a range of $(-\infty,\infty)$
4. **Synthetic Accessibility score (SAS)**[45]: Measurement of the difficulty of synthesizing a compound. It is a score between 0 (difficult) and 1(easy).
5. **BertzCT**[46]: A topological index meant to quantify "complexity" of molecules. It consists of a sum of two terms, one representing the complexity of the bonding, the other representing the complexity of the distribution of heteroatoms.
6. **Quantitative Estimate of Drug-likeness (QED)**[47]: This quantifies drug-likeness by considering the main molecular properties. It ranges from 0 (all properties unfavorable) to 1 (all properties favorable).
7. **Topological Polar Surface Area (TPSA)**: The sum of surface area over all polar atoms. It measures the drug's ability to permeate cell membranes. Molecules with a TPSA greater than 140 $Å^2$ tend to be poor in permeating cell membranes.
8. **Natural Product-likeness score (NPS)**[48]: Score of similarity degree of structural space covered by molecules and natural products. It has a range of (0,1)
9. **FractionCSP3**: The fraction of C atoms that are SP3 hybridized.
10. **N-Atoms**: The number of atoms in the molecule.
11. **N-Rings**: The number of rings in the molecule.

B.3.3 Goal-Directed Benchmarks

Goal-directed learning aims to rediscover known drugs, generate compounds similar to reference ones, conduct multi-objective optimization of known drugs' properties, or perform scaffold hopping. This demonstrates the broad applicability of the t-SMILES approach. We evaluate SMILES, DeepSMILES, SELFIES, t-SMILES and the SOTA baseline model CReM[29] using all 20 goal-directed tasks from the GuacaMol[37] benchmark.

For the goal-directed benchmarks 20 (default setting in GuacaMol) or more iterations of hill-climbing were performed; at each step, the model generated 4096 (default setting is 8192 in GuacaMol) molecules, and the top-scoring 1024 were used to fine-tune the model parameters. In order to be fair when comparing metrics, LSTM models with three layers of hidden size of 512 (default setting is 1024 in GuacaMol) are trained as generative models in this section. For the distribution learning benchmarks, no fine-tuning was done; the model simply generated the required number of molecules. Twenty sub-tasks and benchmarks are listed in Supplementary Table 11.

Supplementary Table 11 Goal-directed benchmarks of baseline model GuacaMol[37] and CReM[29].

ID	Benchmark Name	Best of DataSet[37]	SMILES LSTM[37]	Graph GA[37]	Graph MCTS[37]	CReM[29]
1	Celecoxib rediscovery	0.505	1.000	1.000	0.355	1.000
2	Troglitazone rediscovery	0.419	1.000	1.000	0.311	1.000
3	Thiothixene rediscovery	0.456	1.000	1.000	0.311	1.000
4	Aripiprazole similarity	0.595	1.000	1.000	0.380	1.000
5	Albuterol similarity	0.719	1.000	1.000	0.749	1.000
6	Mestranol similarity	0.629	1.000	1.000	0.402	1.000
7	C11H24	0.684	0.993	0.971	0.410	0.966
8	C9H10N2O2PF2Cl	0.747	0.879	0.982	0.631	0.940
**9**	**Median molecules 1**	**0.334**	**0.438**	**0.406**	**0.225**	**0.371**
10	Median molecules 2	0.351	0.422	0.432	0.170	0.434
11	Osimertinib MPO	0.839	0.907	0.953	0.784	0.995
12	Fexofenadine MPO	0.817	0.959	0.998	0.695	1.000
13	Ranolazine MPO	0.792	0.855	0.920	0.616	0.969
14	Perindopril MPO	0.575	0.808	0.792	0.385	0.815
15	Amlodipine MPO	0.696	0.894	0.894	0.533	0.902
**16**	**Sitagliptin MPO**	**0.509**	**0.545**	**0.891**	**0.458**	**0.763**
17	Zaleplon MPO	0.547	0.669	0.754	0.488	0.770
**18**	**Valsartan SMARTS**	**0.259**	**0.978**	**0.990**	**0.040**	**0.994**
19	Deco hop	0.933	0.996	1.000	0.590	1.000
20	Scaffold hop	0.738	0.998	1.000	0.478	1.000

B.3.3.1 LSTM Models on ChEMBL for Goal Directed Benchmarks

To compare with the GuacaMol benchmark, we retrain all of these models using the same dataset, model architecture (LSTM with 3 layers and a hidden size of 512), and almost identical hyperparameters, apart from the variation in the number of training epochs.

Supplementary Table 12 Distributional results on ChEMBL using LSTM as generative model.

Model	Valid	Unique	Novelty	KLD	FCD	Nov./Uni.
SMILES_LSTM[R10]	0.954	0.954	0.919	0.975	**0.866**	0.964
DSMILES_LSTM[R10]	0.848	0.845	0.814	0.968	0.832	0.963
SELFIES_LSTM[R10]	1.000	1.000	0.979	0.901	0.707	0.979
SELFIES_LSTM[R15]	1.000	1.000	0.979	0.962	0.725	0.979
TS_Vanilla_LSTM[R10]	1.000	0.998	0.934	0.976	**0.866**	0.936
TSDY_HBV_LSTM_[R10]	0.999	0.998	0.960	0.967	0.849	0.962
TSDY_HMV_LSTM_[R15]	1.000	0.999	**0.972**	0.961	0.828	0.972
TSDY_HSV_LSTM_[R15]	1.000	1.000	0.966	0.955	0.841	0.967
TSSA_B_LSTM_[R10]	1.000	0.965	0.940	0.940	0.620	0.974
TSSA_M_LSTM_[R10]	1.000	0.999	0.966	0.963	0.782	0.967
TSSA_S_LSTM_[R10]	1.000	0.995	**0.980**	0.950	0.690	0.985
TSID_M_LSTM [R10]	1.000	0.998	0.972	0.925	0.812	0.974

First, the design logic behind SELFIES and t-SMILES allows theoretical generation of nearly 100% chemically valid molecules. But it is obvious that the SELFIES-based model yields the lowest FCD scores of all the baseline models using nearly similar hyperparameters. DSMILES gets both lower FCD and novelty scores than SMILES. In addition, the t-SMILES family receives significantly higher novelty scores than SMILES and DSMILES.

Regarding the difference scores between classical SMILES and TS_Vanilla, there are some theoretical reasons to consider. Firstly, TS_Vanilla is the t-SMILES style code of classical SMILES, but it needs to be decoded to obtain classical SMILES, so its valid score is 1.0. Additionally, there are some underlying logics to correct invalid strings as valid ones. Due to the length of this paper, further discussion on this topic is beyond the scope of this text. Furthermore, it is recommended to use the Kekule style to describe molecules or fragments as it is more organized and provides clearer information. In this experiment, TS_Vanilla uses Kekule style code, while classical SMILES does not follow the Kekule style.

Finally, it is important to acknowledge the potential impact of experimental error on the performance of the generative model, which is based on probability. The scores are similar but slightly different.

B.3.3.2 Physicochemical Properties for LSTM Models on ChEMBL

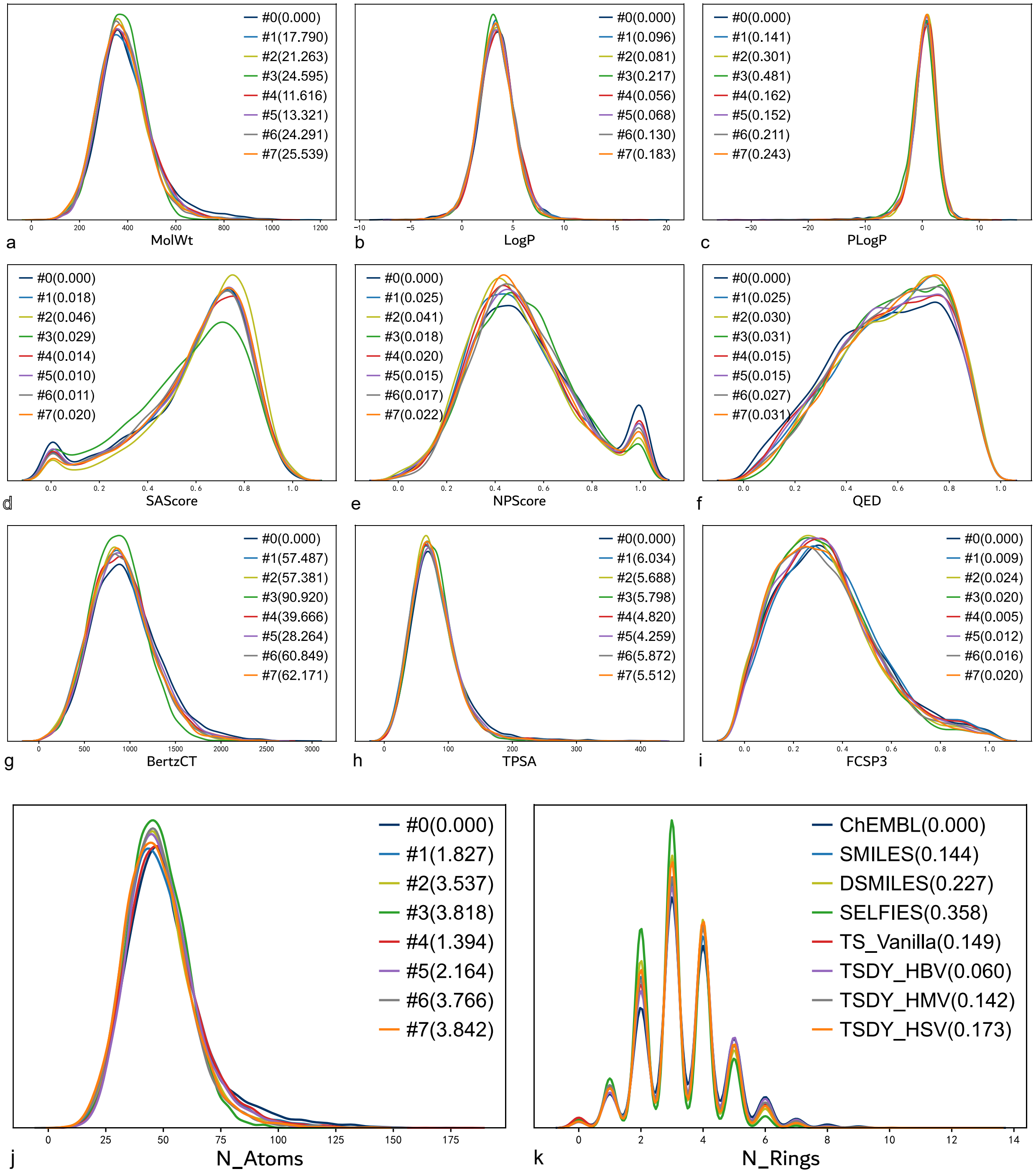

Supplementary Fig.8 Physicochemical Properties for LSTM Models on ChEMBL. The unit of the y-axis is 'Density'. The figures contain labels ['#0', '#1', '#2', '#3', '#4', '#5', '#6', '#7'] corresponding to the following datasets: ['ChEMBL', 'SMILES', 'DSMILES', 'SELFIES', 'TS_Vanilla', 'TSDY_HBV', 'TSDY_HMV', 'TSDY_HSV'].

B.3.3.3 Goal-Directed Benchmarks on ChEMBL

Supplementary Table 13 Results Goal-Directed Benchmarks on ChEMBL. SMILES[37] is taken from GuacaMol[37]. We mark the top two highest scores in red and two lowest scores in blue. SM means SMILES, DS means DSMILES, SF means SELFIES.

ID	Benchmark Name	SMILES[37]	CReM	SM	DS	SF	TS_V	TSDY_B	TSDY_M	TSDY_S
1	Celecoxib rediscovery	1.000	1.000	1.000	1.000	1.000	1.000	1.000	1.000	1.000
2	Troglitazone rediscovery	1.000	1.000	1.000	1.000	1.000	1.000	1.000	1.000	1.000
3	Thiothixene rediscovery	1.000	1.000	1.000	1.000	1.000	1.000	1.000	1.000	1.000
4	Aripiprazole similarity	1.000	1.000	1.000	1.000	1.000	1.000	1.000	1.000	1.000
5	Albuterol similarity	1.000	1.000	1.000	1.000	1.000	1.000	**0.980**	1.000	1.000
6	Mestranol similarity	1.000	1.000	1.000	1.000	1.000	1.000	0.999	1.000	1.000
7	C11H24	0.993	0.966	0.968	0.974	0.975	**0.957**	**0.899**	**0.983**	**0.978**
8	C9H10N2O2PF2Cl	0.879	**0.940**	0.891	0.873	**0.858**	0.862	**0.750**	**0.956**	0.873
**9**	**Median molecules 1**	0.438	**0.360**	**0.428**	**0.371**	0.380	0.393	**0.428**	0.398	0.398
10	Median molecules 2	0.422	0.402	**0.408**	0.398	**0.355**	**0.403**	**0.361**	0.399	0.399
11	Osimertinib MPO	0.907	**0.942**	**0.918**	0.900	**0.891**	0.897	**0.845**	0.916	0.914
12	Fexofenadine MPO	0.959	**0.999**	0.974	0.957	0.963	0.964	**0.936**	**0.953**	**0.982**
13	Ranolazine MPO	0.855	**0.962**	0.850	**0.833**	0.837	0.839	**0.817**	0.834	**0.872**
14	Perindopril MPO	0.808	0.744	**0.810**	0.756	**0.719**	**0.738**	0.798	**0.805**	0.759
15	Amlodipine MPO	0.894	**0.895**	0.886	**0.869**	0.887	0.883	**0.897**	**0.781**	0.875
**16**	**Sitagliptin MPO**	0.545	**0.708**	0.552	**0.537**	0.565	**0.567**	**0.510**	0.547	0.562
17	Zaleplon MPO	0.669	**0.723**	0.597	**0.589**	0.606	0.598	**0.546**	**0.667**	0.605
**18**	**Valsartan SMARTS**	0.978	**0.987**	0.960	0.952	0.962	0.943	**0.905**	**0.864**	**0.974**
19	Deco Hop	0.996	**0.966**	**0.996**	0.982	0.989	**0.996**	0.984	**0.971**	**1.000**
20	Scaffold Hop	0.998	**0.848**	0.997	0.997	0.987	0.993	**1.000**	**0.962**	0.987

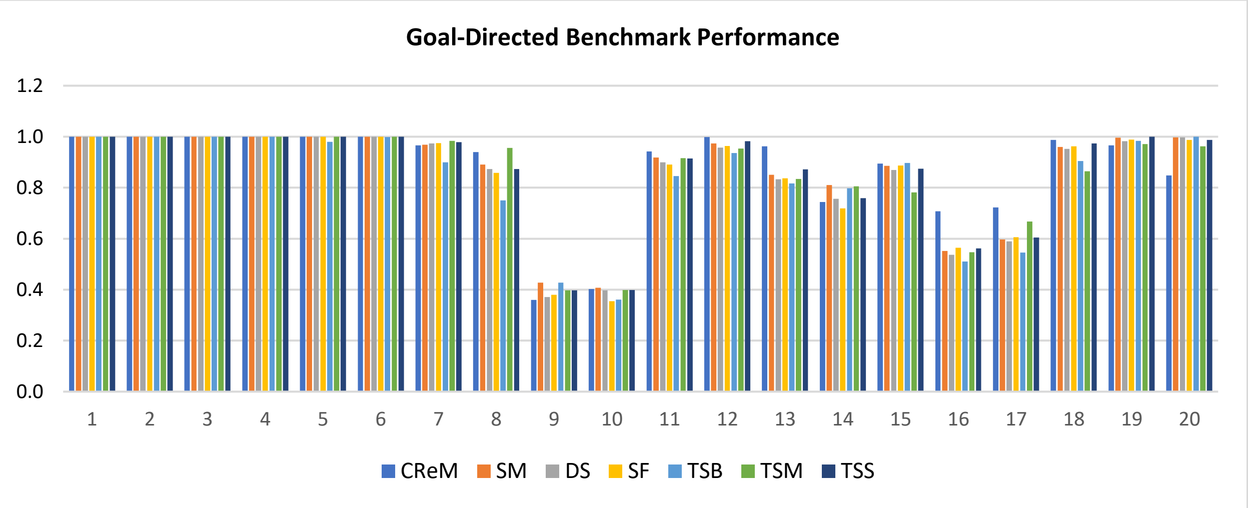

Supplementary Fig.9 Performance of the Goal-Directed Benchmarks.

Further in-depth investigation shows that the models are not adequately trained when using the default settings for some subtasks. Consequently, this result serves as a starting point for further research with more training epochs and the goal-directed reconstruction algorithm in B.3.3.4.

B.3.3.4 Goal-Directed Benchmarks for T16.SMPO

In Supplementary Fig.10 and Supplementary Fig.11, for t-SMILES family, random(R) and goal-directed(G) reconstruction are evaluated. 'TS' in TSBR, TSBG, TSSR, TSSG, TSMR and TSMG means TSDY models. 'SM' in SMG means SMILES model, 'DS' in DSG means DSMILES model, 'SF' in SFG means SELFIES model. 'G' in SMG, DSG and SFG means more training rounds with 100. 'R50' means 50 training rounds. 10 or 15 random candidates are selected to calculate scores and the top-scoring one is chosen for output.

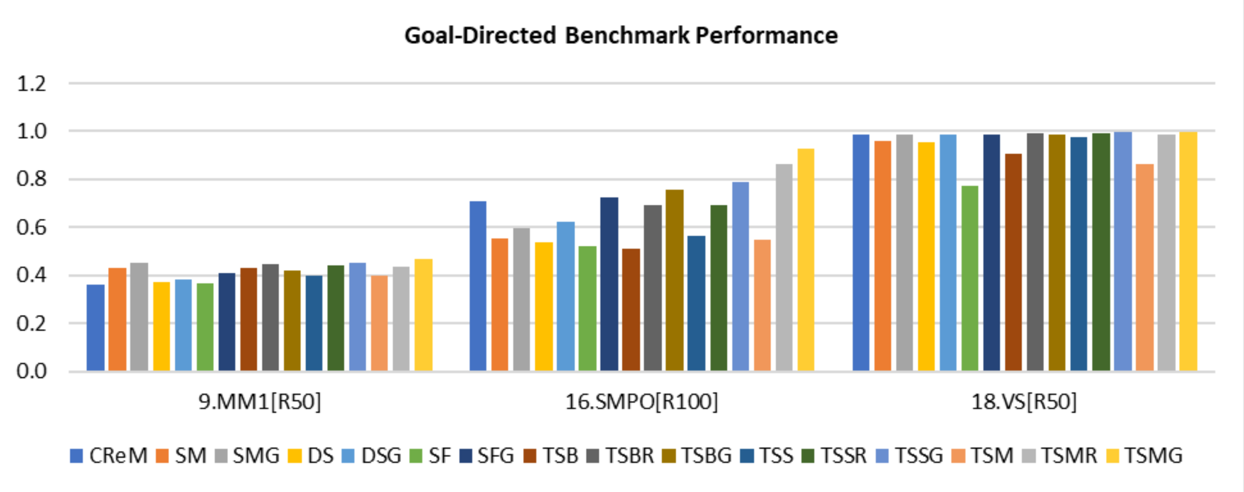

Supplementary Fig.10 Performance of the Goal-Directed Benchmarks for T16.SMPO.

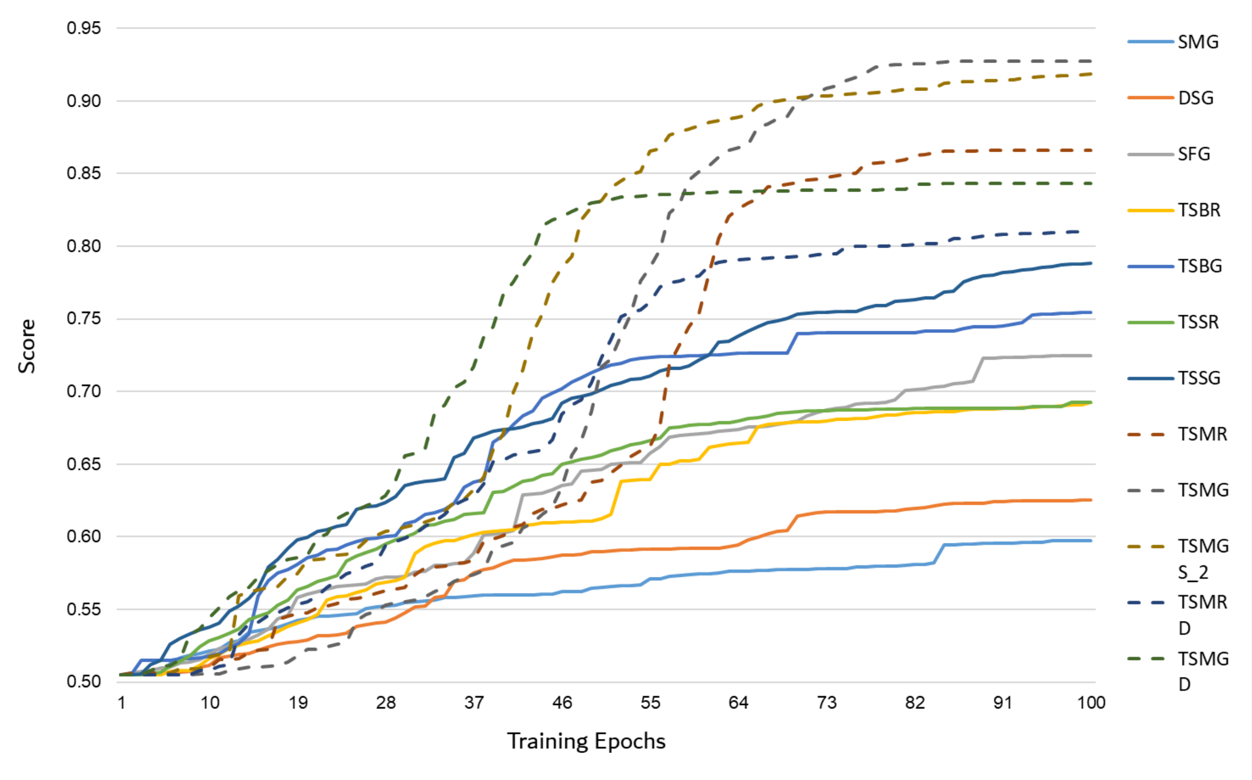

Supplementary Fig.11 Performance of the Goal-Directed Benchmarks for T16.SMPO with different training epochs. The TSMG yields significantly high results, and therefore, we conduct multiple executions to confirm it thoroughly. S: GPU 1: NVIDIA GeForce RTX 3090, D: GPU 2: NVIDIA Quadro RTX4000.

In drug discovery, as in many domains, the development process is iterative rather than analytical. In the experiments of the low-resource dataset JNK3, we demonstrate a single round iterative process, where the model that uses reconstructed active molecules as training data achieved the highest novel-active score of 0.829, compared to 0.526 of the SMILES model. The iterative advantage of t-SMILES in this goal-directed evaluation provides further evidence of its underlying benefits, particularly in achieving a higher novelty score.

This experiment is expected to yield highly significant results as it is designed to test the limits of different codes. The findings suggest that t-SMILES could have further applicability in reaching the limit of the target function, and ultimately help chemists create more rational target functions. For the related generated molecules, please refer to section SI.B.3.3.8.

When reconstructing molecules, we randomly select ten or fifteen (depending on GPU and memory) candidates to calculate their scores. The candidate with the highest score is chosen as the output. It is advisable to average the scores over multiple runs for a general report.

In addition, this study provides more insights on low-resource tasks:

First, whether the objective function has a high score solution is determined by the function itself.

Second, whether the highest score can be figured out is determined by the choice of molecular descriptions, models, and optimization methods.

Based on Supplementary Fig.10, Supplementary Fig.11 and experiments on JNK3 and AID1706, t-SMILES models exhibit the potential to achieve higher scores through appropriate optimization processes.

Drug discovery is inherently a multi-objective optimization (MOO) problem, where optimizing for one property may have negative impacts on others. From SI.B.3.3.5, SI.B.3.3.6 and SI.B.3.3.8, it can be seen that there is significant variation in physicochemical properties. This means that in real-world molecular modeling tasks, medicinal chemists typically face challenging MOO problems, with far more potential choices than can be systematically explored. Therefore, it is more advantageous to use hybrid t-SMILES models to obtain favorable results because they can explore larger chemical spaces.

B.3.3.5 Goal-Directed Benchmarks for T16.SMPO using TSSA and TSID

In Supplementary Table 14 and Supplementary Fig.12, For t-SMILES family, random(R) and goal-directed(G) reconstruction are evaluated. 'G' in SMG, DSG and SFG means 100 training rounds, TS in TSBR, TSBG, TSSR, TSSG, TSMR and TSMG means TSDY. 'R50' means 50 training rounds. 10 or 15 random candidates are selected to calculate scores and the top-scoring one is chosen for output. GPU 1: NVIDIA GeForce RTX 3090 and GPU 2: NVIDIA Quadro RTX4000 are using for this experiment.

Supplementary Table 14 Results of the Goal-Directed Benchmarks for T16.SMPO with different training epochs.

Round	SMG	DSG	SFG	TSBR	TSBG	TSSR	TSSG	TSMR	TSMG	TSSA_BG	TSSA_MG	TSSA_SG	TSSA_MR	TSSA_MR	TSID_M
1	0.505	0.505	0.505	0.505	0.505	0.505	0.505	0.505	0.505	0.505	0.505	0.505	0.505	0.505	0.505
2	0.505	0.505	0.507	0.505	0.505	0.505	0.505	0.506	0.505	0.505	0.505	0.509	0.505	0.505	0.505
3	0.505	0.505	0.507	0.505	**0.515**	0.505	0.506	0.506	0.505	0.505	0.505	**0.514**	0.505	0.508	0.505
4	0.506	0.506	0.508	0.505	**0.515**	0.506	0.512	0.506	0.505	0.505	0.505	**0.516**	0.505	0.508	0.505
5	0.506	0.506	0.509	0.505	**0.515**	0.507	**0.515**	0.506	0.505	0.508	0.507	**0.523**	0.507	0.509	0.505
10	0.521	0.512	0.519	0.516	0.518	0.528	**0.538**	0.510	0.505	0.518	0.516	**0.575**	0.515	0.538	0.505
**20**	0.544	0.529	0.560	0.543	**0.586**	**0.566**	**0.600**	**0.548**	0.523	0.540	**0.547**	**0.656**	**0.547**	**0.618**	0.505
**30**	0.555	0.552	0.576	**0.589**	**0.611**	**0.602**	**0.637**	**0.574**	**0.556**	**0.564**	**0.590**	**0.687**	**0.562**	**0.643**	0.505
45	0.561	0.586	0.632	0.610	**0.699**	0.644	0.681	0.620	0.621	0.605	**0.774**	0.770	0.600	0.676	0.505
50	0.565	0.590	0.646	0.612	0.716	0.656	0.701	0.639	**0.717**	0.608	**0.812**	0.778	0.620	0.685	0.505
90	0.596	0.624	0.723	0.688	0.745	0.689	0.780	0.866	**0.928**	0.659	**0.816**	0.780	0.713	0.756	0.506
100	0.598	0.625	0.725	0.692	0.755	0.693	0.788	0.866	**0.928**	0.669	**0.830**	0.780	0.717	0.761	0.506

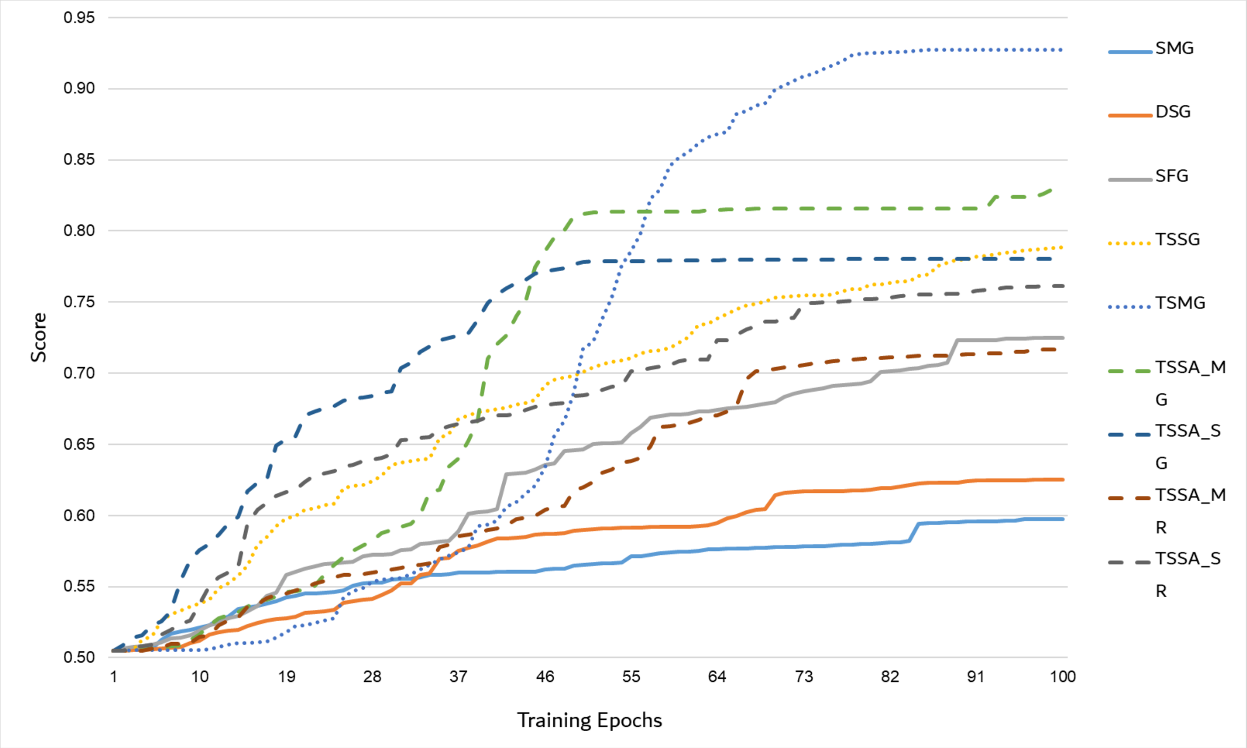

Supplementary Fig.12 Performance of the Goal-Directed Benchmarks for T16.SMPO with different training epochs.

Tables and Figures show that when the training round reaches 20, the default setting of GuacaMol, TSSA_M and TSSA_S also achieve higher scores than SMILES model. In particular, TSSA_S achieves a score of 0.656, compared to the TSDY_S model's score of 0.600.

If training with more 10 rounds to 30, all TSDY and TSSA models can outperform SMILES based models.

These results indicate at least three points:

1) TSSA models, similar to TSDY models, can outperform models based on SMILES, DSMILES, SELFIES, and the baseline model CReM.
2) It is possible to optimize both TSSA and TSDY models to explore the limits of the target problem, although it requires skillful optimization techniques. In our experiments, we calculated scores for only 10 randomly selected candidates(it is a very simple way), not using an exhaustive search algorithm[50].
3) TSID-based models have limited ability compared to TSSA and TSDY based models for goal-oriented tasks.

B.3.3.6 Physicochemical Properties for T18.Valsartan SMARTS

Supplementary Table 15 T18.Valsartan SMARTS take sitagliptin and valsartan to calculate the final score.

Target Molecule	MolWt	LogP	BertzCT	TPSA
Sitagliptin	407.318	2.016	896.38	77.04
Valsartan	225.291	2.942	476.035	20.31

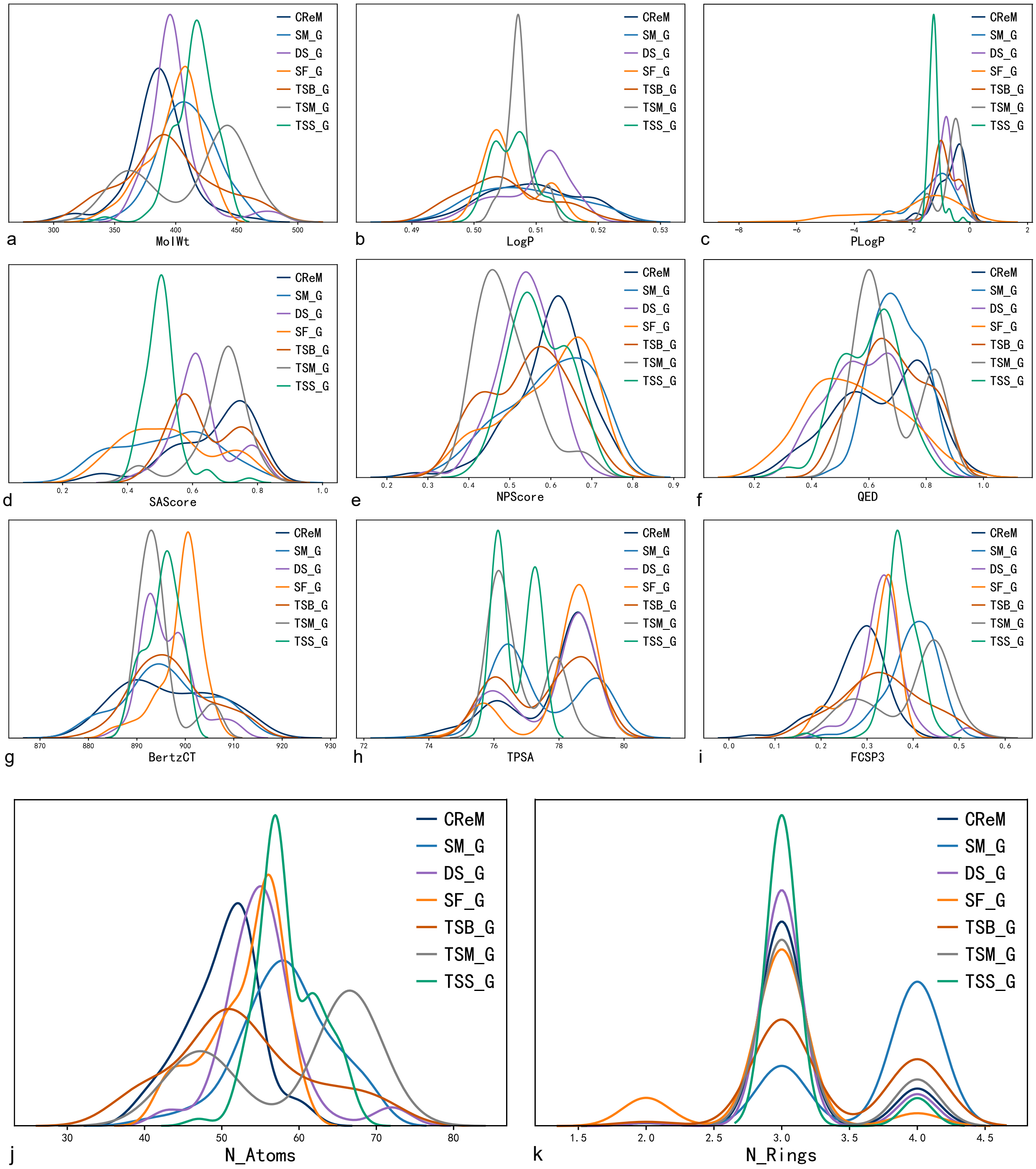

Supplementary Fig.13 Physicochemical Properties of Baseline models and TSSA modes with goal-directed reconstruction algorithms for T18 Valsartan SMARTS. The unit of the y-axis is 'Density'.

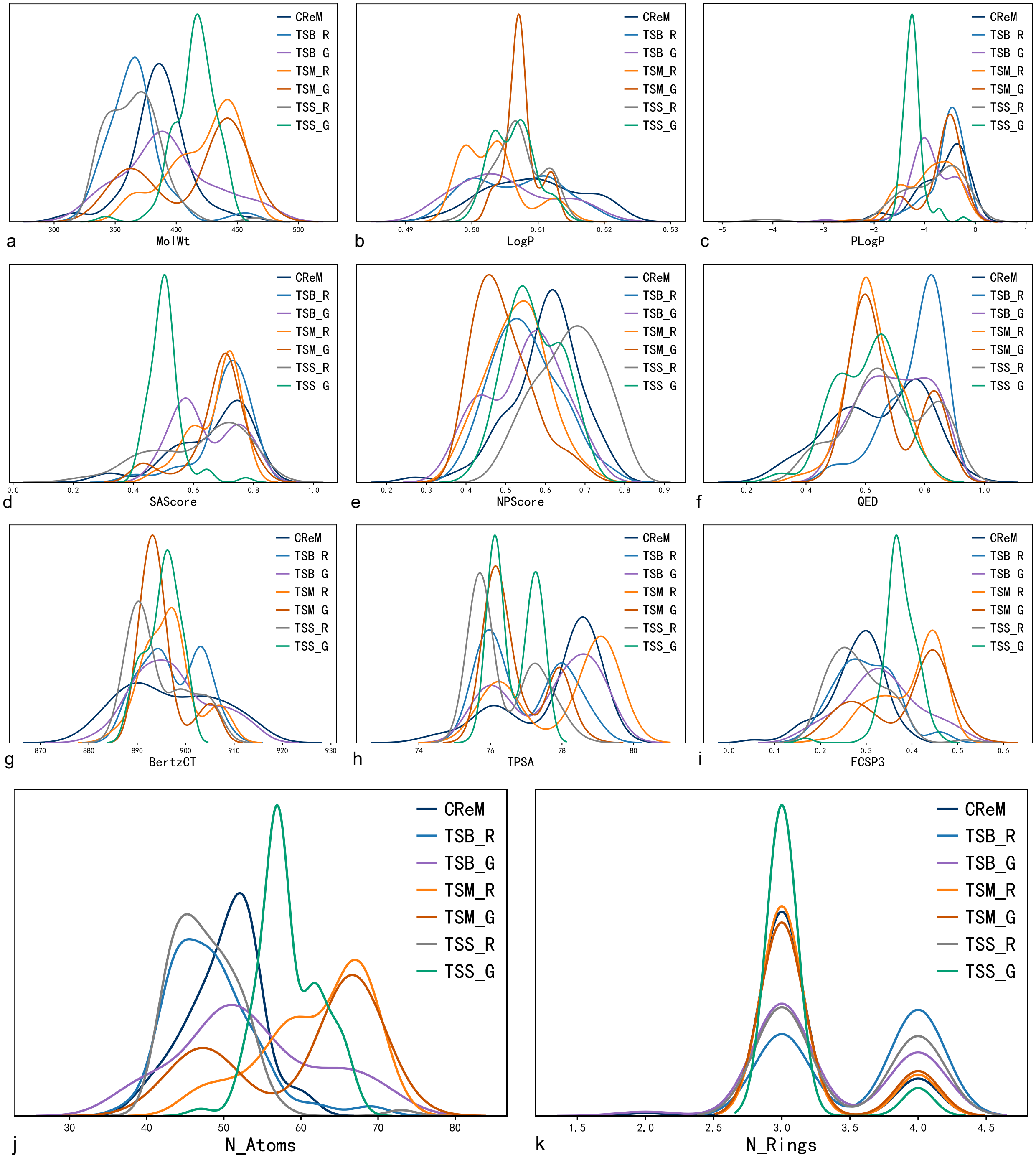

Supplementary Fig.14 Physicochemical Properties of CReM and TSSA modes using random and goal-directed reconstruction algorithms for T18 Valsartan SMARTS. The unit of the y-axis is 'Density'.

B.3.3.7 Top 15 Generated Molecules for T18.Valsartan SMARTS

TSDY_B

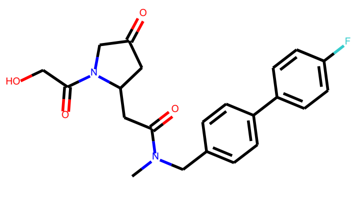

LP:2.0, BC:896.0, TA:77.9

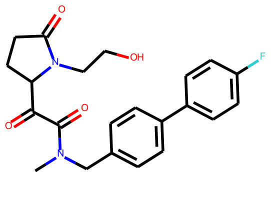

LP:2.0, BC:896.0, TA:77.9

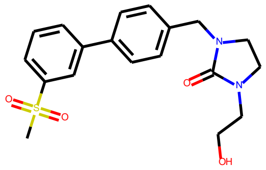

LP:2.0, BC:894.0, TA:77.9

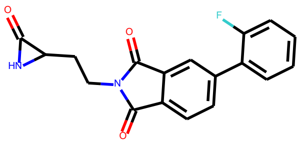

LP:2.0, BC:893.6, TA:76.4

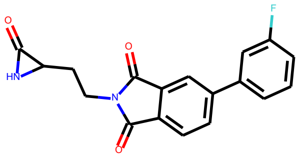

LP:2.0, BC:893.6, TA:76.4

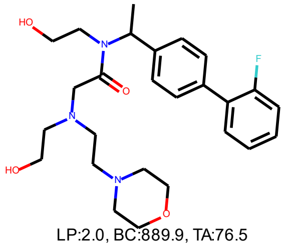

LP:2.0, BC:889.9, TA:76.5

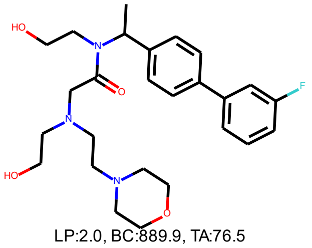

LP:2.0, BC:889.9, TA:76.5

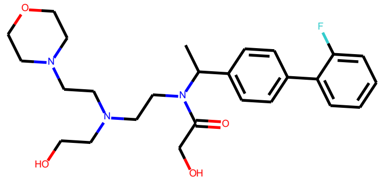

LP:2.0, BC:889.9, TA:76.5

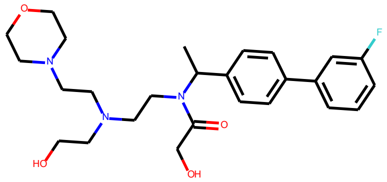

LP:2.0, BC:889.9, TA:76.5

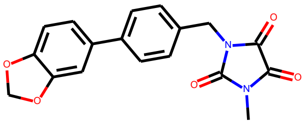

LP:2.0, BC:890.8, TA:76.2

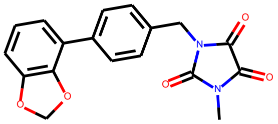

LP:2.0, BC:890.8, TA:76.2

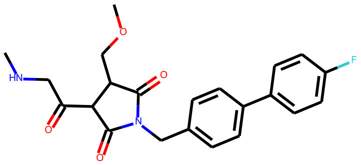

LP:2.0, BC:896.0, TA:75.7

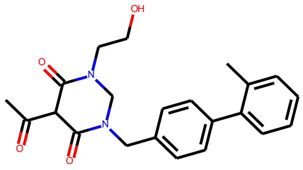

LP:2.0, BC:891.7, TA:77.9

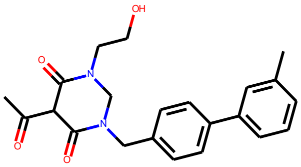

LP:2.0, BC:891.7, TA:77.9

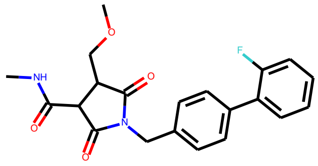

LP:2.0, BC:897.5, TA:75.7

Supplementary Fig.15 Top 15 generated molecules for T18.Valsartan SMARTS of **TSDY_B**

TSDY_M

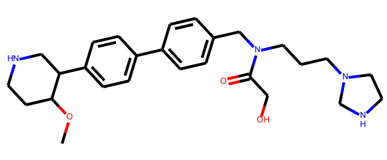

LP:2.0, BC:897.1, TA:77.1

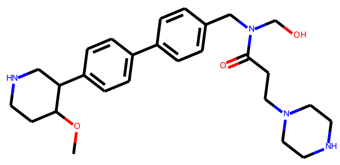

LP:2.0, BC:897.1, TA:77.1

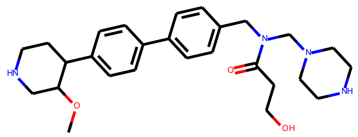

LP:2.0, BC:897.1, TA:77.1

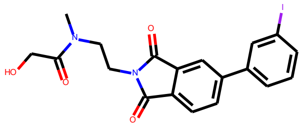

LP:2.0, BC:896.9, TA:77.9

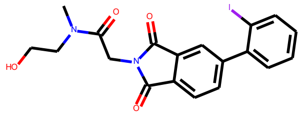

LP:2.0, BC:896.9, TA:77.9

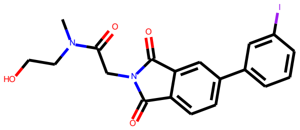

LP:2.0, BC:896.9, TA:77.9

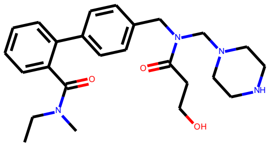

LP:2.0, BC:894.3, TA:76.1

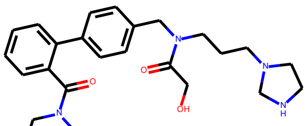

LP:2.0, BC:894.3, TA:76.1

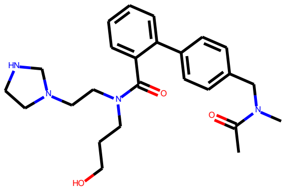

LP:2.0, BC:894.3, TA:76.1

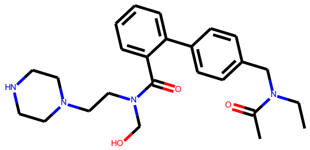

LP:2.0, BC:894.3, TA:76.1

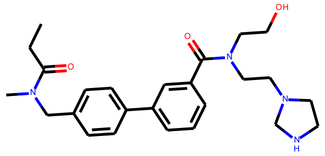

LP:2.0, BC:894.3, TA:76.1

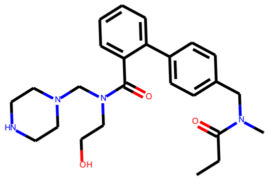

LP:2.0, BC:894.3, TA:76.1

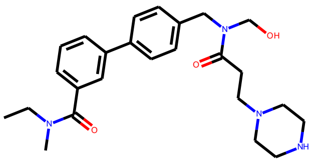

LP:2.0, BC:894.3, TA:76.1

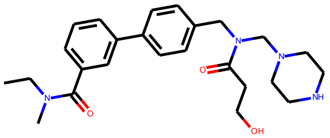

LP:2.0, BC:894.3, TA:76.1

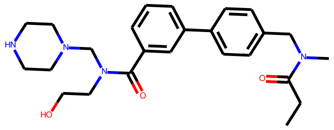

LP:2.0, BC:894.3, TA:76.1

Supplementary Fig.16 Top 15 generated molecules for T18.Valsartan SMARTS of **TSDY_M**

TSDY_S

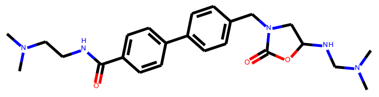

LP:2.0, BC:896.9, TA:77.2

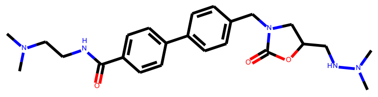

LP:2.0, BC:896.9, TA:77.2

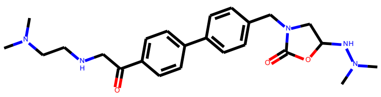

LP:2.0, BC:896.9, TA:77.2

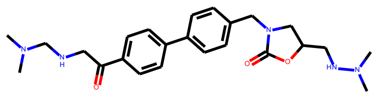

LP:2.0, BC:896.9, TA:77.2

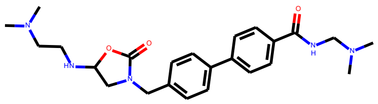

LP:2.0, BC:896.9, TA:77.2

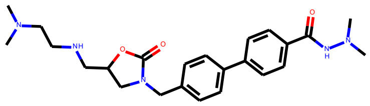

LP:2.0, BC:896.9, TA:77.2

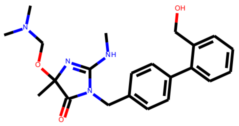

LP:2.0, BC:895.2, TA:77.4

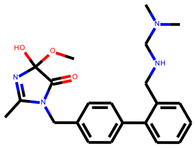

LP:2.0, BC:895.2, TA:77.4

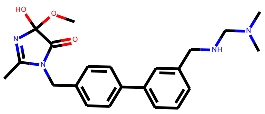

LP:2.0, BC:895.2, TA:77.4

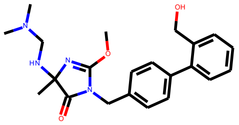

LP:2.0, BC:895.2, TA:77.4

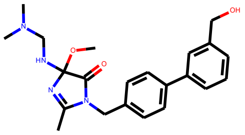

LP:2.0, BC:895.2, TA:77.4

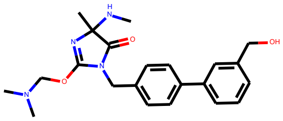

LP:2.0, BC:895.2, TA:77.4

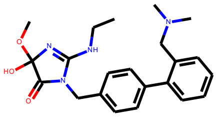

LP:2.0, BC:895.2, TA:77.4

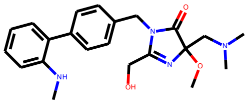

LP:2.0, BC:895.2, TA:77.4

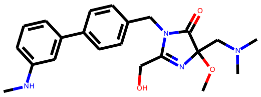

LP:2.0, BC:895.2, TA:77.4

Supplementary Fig.17 Top 15 generated molecules for T18.Valsartan SMARTS of **TSDY_S**

CReM

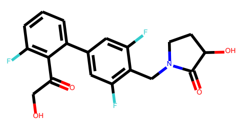

LP:2.0, BC:892.8, TA:77.8

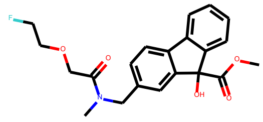

LP:2.0, BC:900.1, TA:76.1

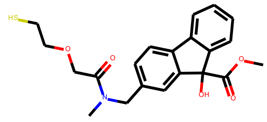

LP:2.0, BC:900.1, TA:76.1

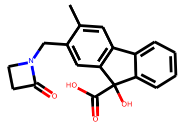

LP:2.0, BC:888.9, TA:77.8

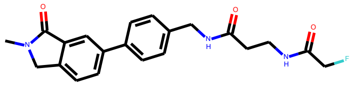

LP:2.0, BC:896.3, TA:78.5

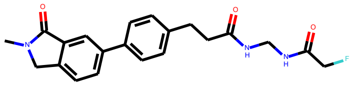

LP:2.0, BC:896.3, TA:78.5

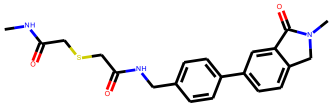

LP:2.0, BC:896.3, TA:78.5

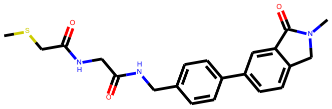

LP:2.0, BC:896.3, TA:78.5

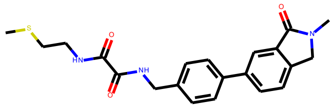

LP:2.0, BC:896.3, TA:78.5

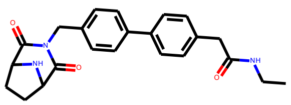

LP:2.0, BC:899.4, TA:78.5

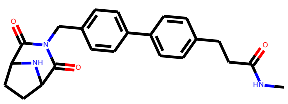

LP:2.0, BC:899.4, TA:78.5

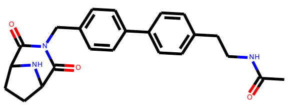

LP:2.0, BC:899.4, TA:78.5

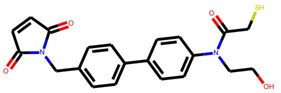

LP:2.0, BC:889.0, TA:77.9

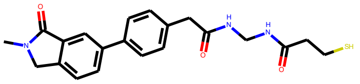

LP:2.0, BC:896.3, TA:78.5

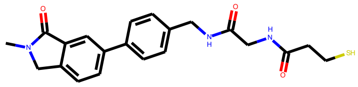

LP:2.0, BC:896.3, TA:78.5

Supplementary Fig.18 Top 15 generated molecules for T18.Valsartan SMARTS of **CREM**

DSMILES

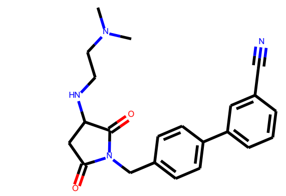

LP:2.0, BC:899.5, TA:76.4

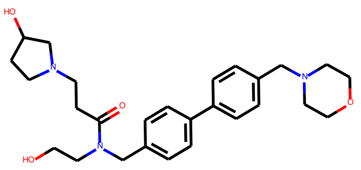

LP:2.0, BC:897.1, TA:76.5

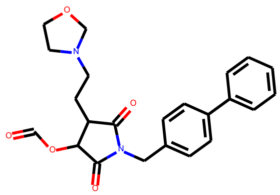

LP:2.1, BC:893.6, TA:76.2

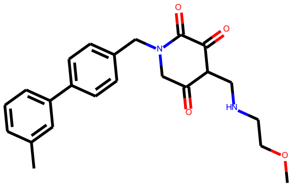

LP:2.0, BC:892.0, TA:75.7

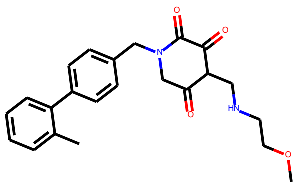

LP:2.0, BC:892.0, TA:75.7

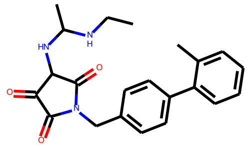

LP:2.0, BC:892.2, TA:78.5

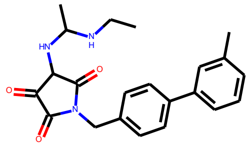

LP:2.0, BC:892.2, TA:78.5

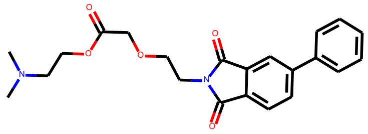

LP:2.1, BC:895.3, TA:76.2

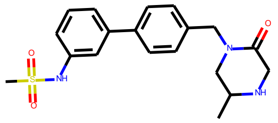

LP:2.0, BC:894.5, TA:78.5

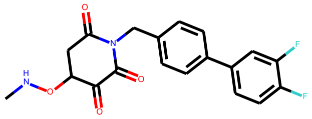

LP:2.0, BC:899.1, TA:75.7

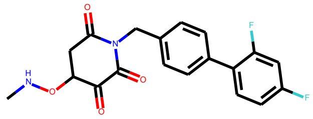

LP:2.0, BC:899.1, TA:75.7

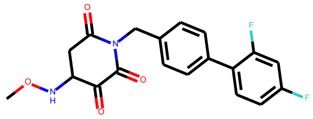

LP:2.0, BC:899.1, TA:75.7

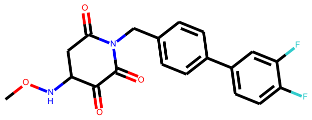

LP:2.0, BC:899.1, TA:75.7

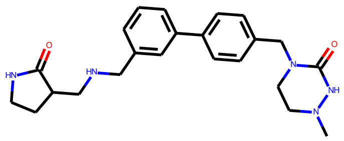

LP:2.0, BC:898.7, TA:76.7

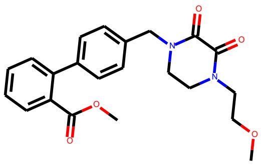

LP:2.0, BC:894.3, TA:76.2

Supplementary Fig.19 Top 15 generated molecules for T18.Valsartan SMARTS of **DSMILES**

SMILES

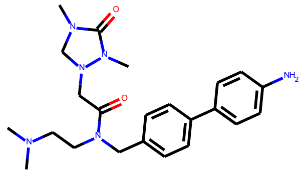

LP:2.0, BC:897.3, TA:76.4

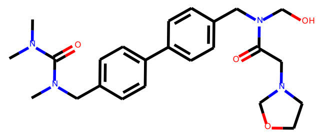

LP:2.0, BC:893.2, TA:76.6

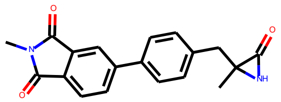

LP:2.0, BC:901.3, TA:76.4

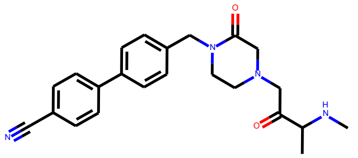

LP:2.0, BC:900.0, TA:76.4

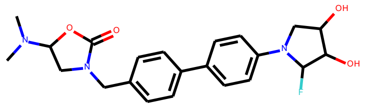

LP:2.0, BC:890.3, TA:76.5

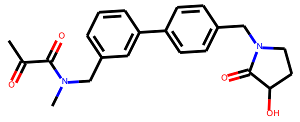

LP:2.0, BC:891.7, TA:77.9

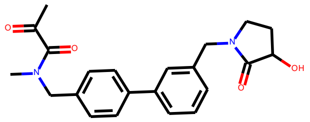

LP:2.0, BC:891.7, TA:77.9

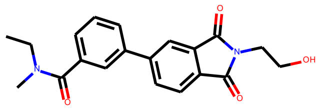

LP:2.0, BC:891.1, TA:77.9

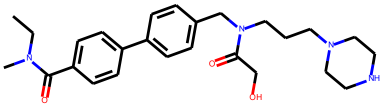

LP:2.1, BC:892.8, TA:76.1

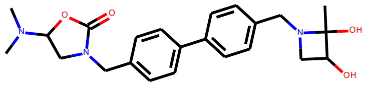

LP:2.1, BC:895.7, TA:76.5

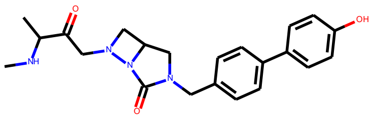

LP:2.1, BC:897.3, TA:76.1

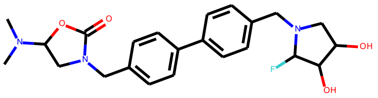

LP:2.0, BC:906.0, TA:76.5

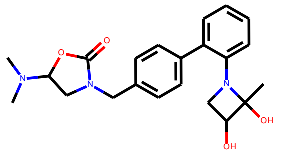

LP:2.1, BC:897.2, TA:76.5

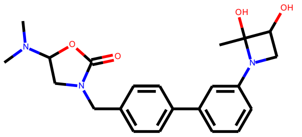

LP:2.1, BC:897.2, TA:76.5

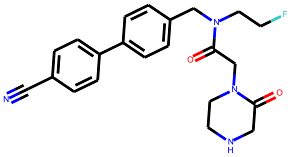

LP:2.0, BC:890.5, TA:76.4

Supplementary Fig.20 Top 15 generated molecules for T18.Valsartan SMARTS of **SMILES**

SELFIES

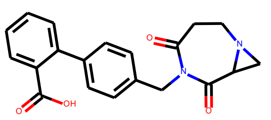
LP:2.0, BC:890.9, TA:77.7

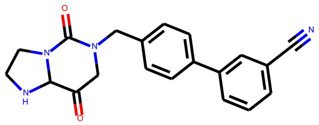
LP:2.0, BC:900.8, TA:76.4

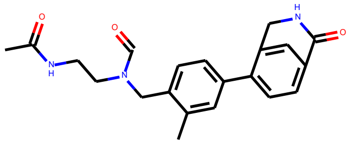
LP:2.0, BC:892.2, TA:78.5

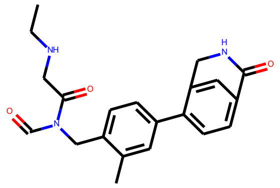
LP:2.0, BC:892.2, TA:78.5

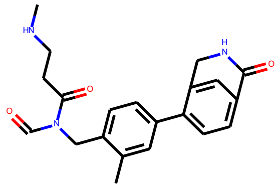
LP:2.0, BC:892.2, TA:78.5

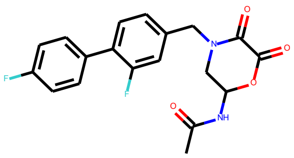
LP:2.0, BC:899.1, TA:75.7

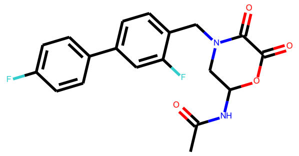
LP:2.0, BC:899.1, TA:75.7

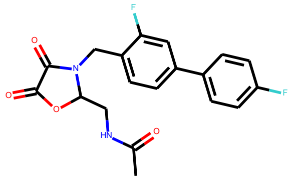
LP:2.0, BC:899.1, TA:75.7

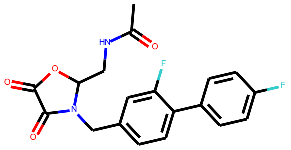
LP:2.0, BC:899.1, TA:75.7

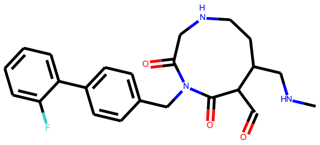
LP:2.0, BC:900.8, TA:78.5

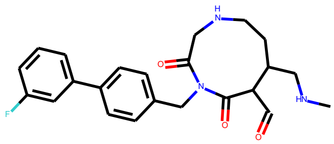
LP:2.0, BC:900.8, TA:78.5

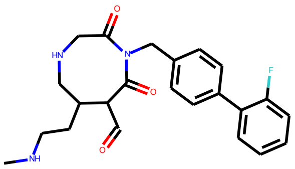
LP:2.0, BC:900.8, TA:78.5

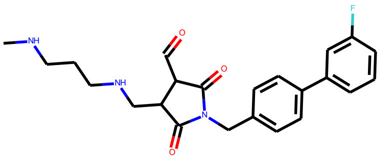
LP:2.0, BC:900.8, TA:78.5

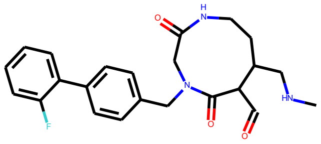
LP:2.0, BC:900.8, TA:78.5

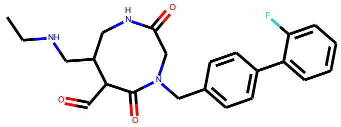
LP:2.0, BC:900.8, TA:78.5

Supplementary Fig.21 Top 15 generated molecules for T18.Valsartan SMARTS of **SELFIES**

B.3.3.8 Top 15 Generated Molecules for T9.Median Molecules 1 Using TSMG

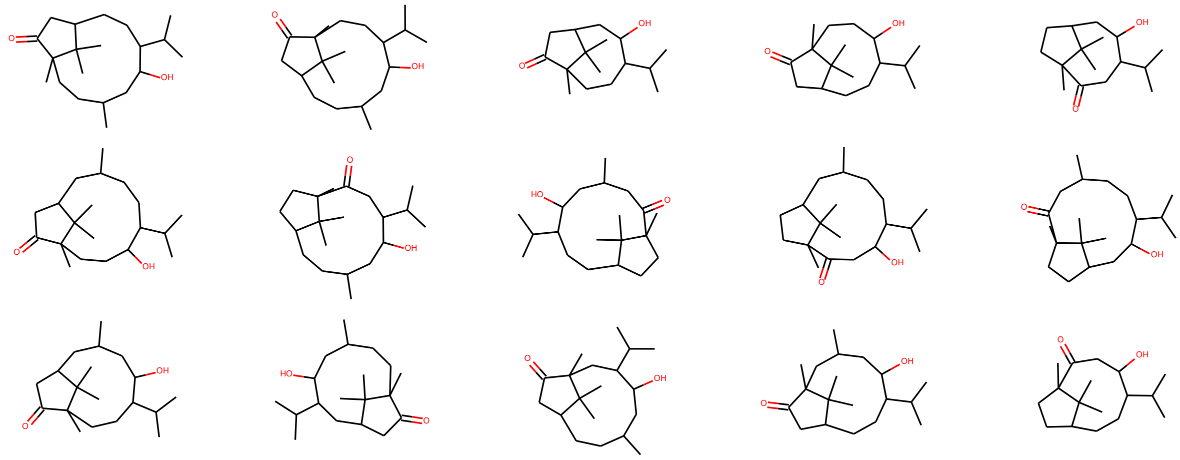

Supplementary Fig.22 Top 15 generated molecules for T9.Median Molecules 1 Using TSMG(TSDY_M with goal directed reconstruction algorithm)

B.3.3.9 Top 10 Generated Molecules for T16.Sitagliptin MPO

Target: Find a molecule dissimilar to sitagliptin, but with the same properties: logP and TPSA.

In real-world application, additional rule, such as Lipinski's Rule-of-Five[51] would be applied to exclude those compounds that do not satisfy drug-likeness criteria.

TSDY_M[0.930]

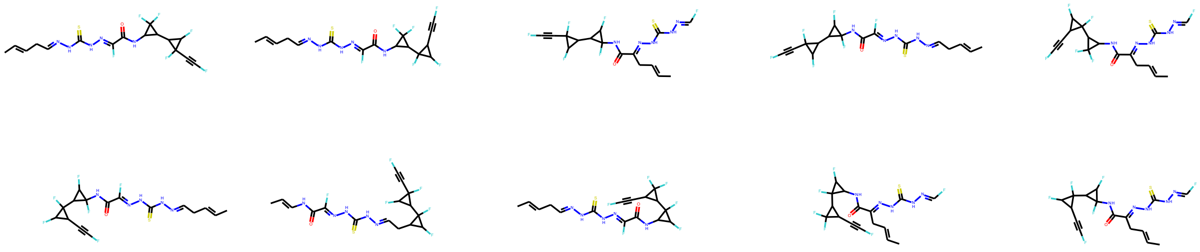

Supplementary Fig.23 Top 10 generated molecules for T16.Sitagliptin MPO of TSDY_M[0.930].

TSDY_M[0.799]

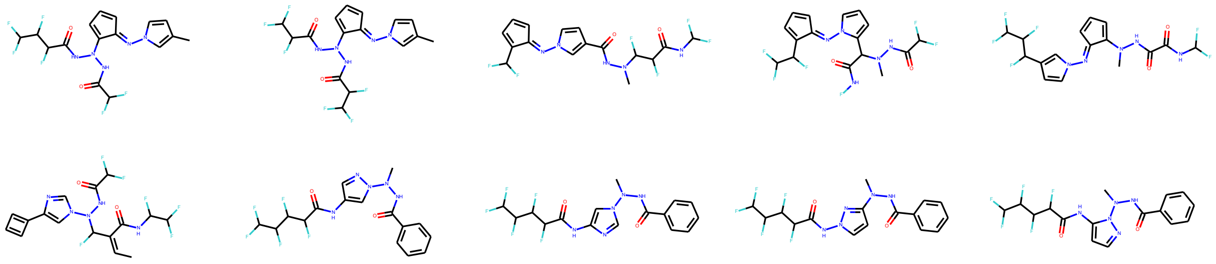

Supplementary Fig.24 Top 10 generated molecules for T16.Sitagliptin MPO of TSDY_M[0.799].

TSDY_B[0.755]

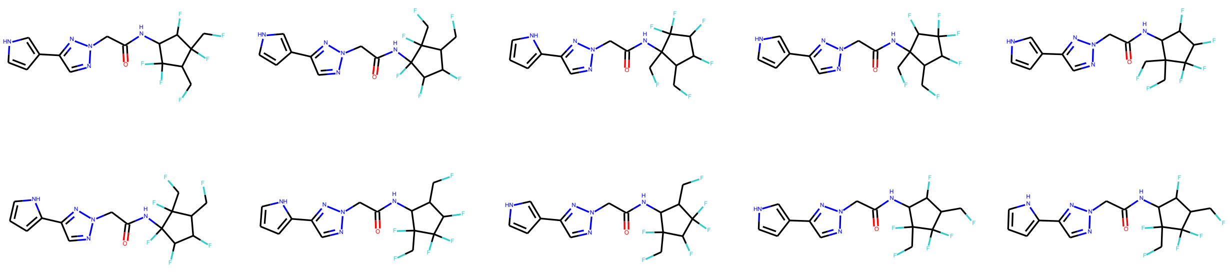

Supplementary Fig.25 Top 10 generated molecules for T16.Sitagliptin MPO **of TSDY_B[0.755].**

TSDY_S[0.788]

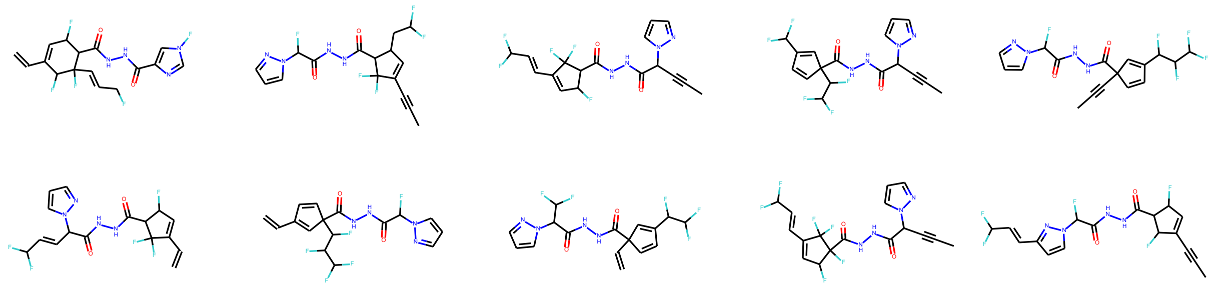

Supplementary Fig.26 Top 10 generated molecules for T16.Sitagliptin MPO of TSDY_S[0.788].

TSSA_B[0.669]

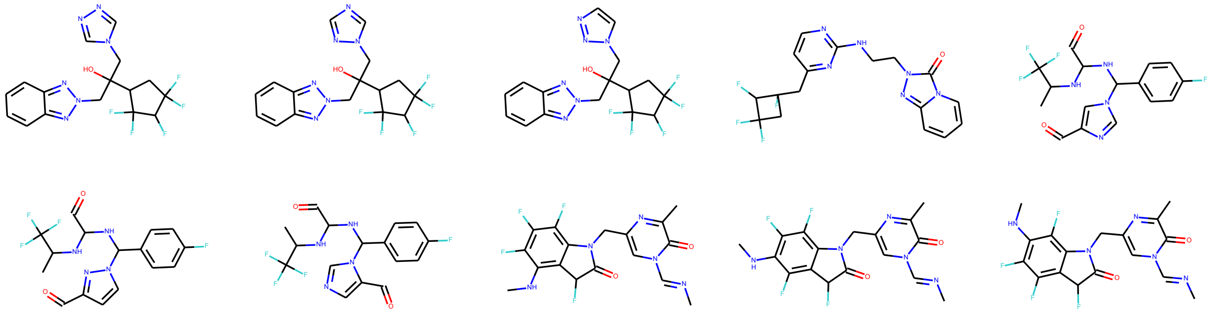

Supplementary Fig.27 Top 10 generated molecules for T16.Sitagliptin MPO of TSSA_B[0.669].

TSSA_M[0.717]

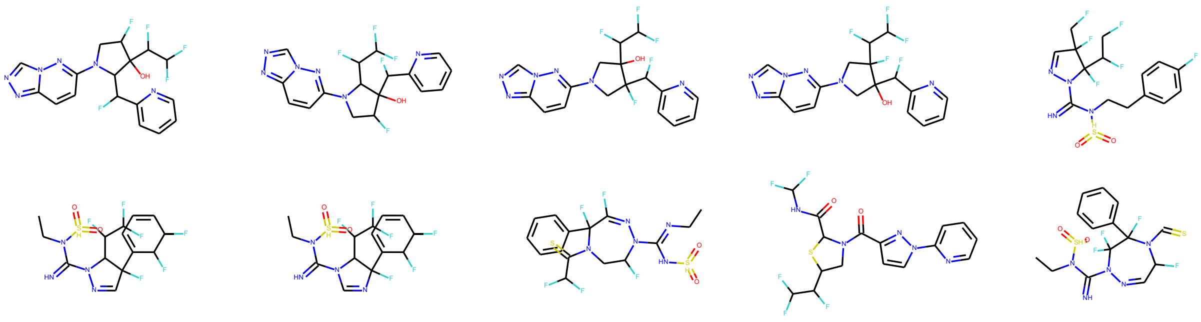

Supplementary Fig.28 Top 10 generated molecules for T16.Sitagliptin MPO of TSSA_M[0.717].

TSSA_S[0.761]

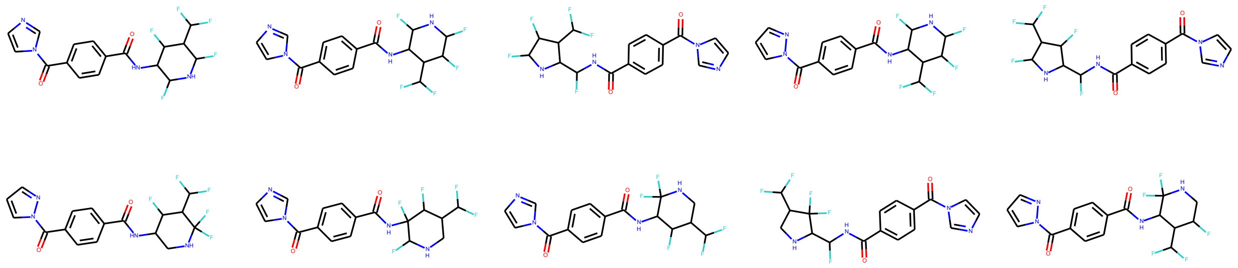

Supplementary Fig.29 Top 10 generated molecules for T16.Sitagliptin MPO of TSSA_S[0.761].

SELFIES[0.725]

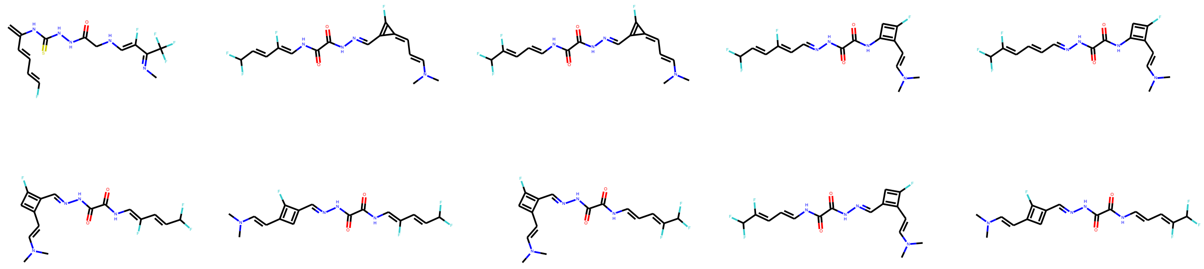

Supplementary Fig.30 Top 10 generated molecules for T16.Sitagliptin MPO of SELFIES[0.725].

SMILES[0.630]

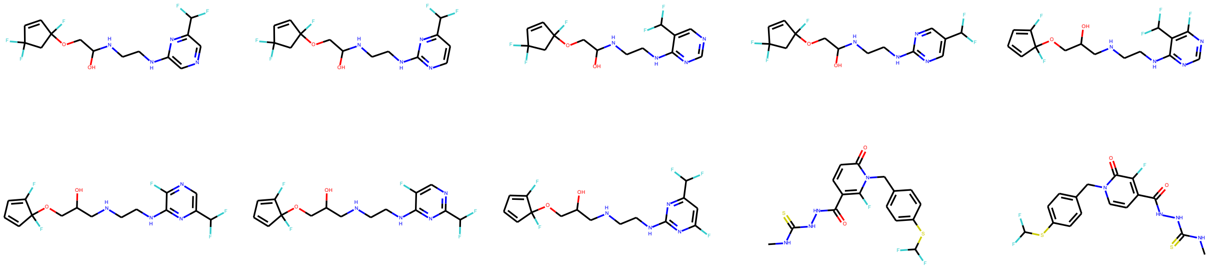

Supplementary Fig.31 Top 10 generated molecules for T16.Sitagliptin MPO of SMILES[0.630].

DSMILES[0.625]

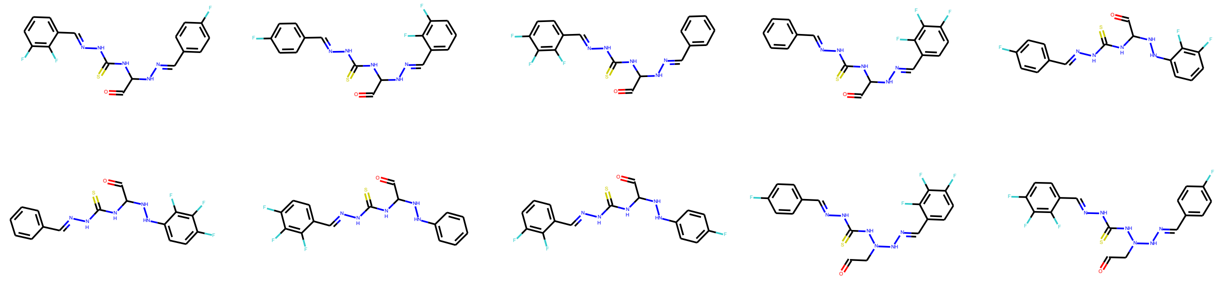

Supplementary Fig.32 Top 10 generated molecules for T16.Sitagliptin MPO of DSMILES[0.625].

C. More Experimental Results on Distinctive Properties

C.1 Distributions of Tokens on Zinc

Supplementary Table 16 Distributions of Tokens on Zinc

SMILES		DeepSMILES		Selfies	
c	0.23	)	0.31	[C]	0.38
C	0.19	c	0.22	[=C]	0.12
(	0.10079	C	0.18	[Ring1]	0.09
)	0.10079	O	0.05	[Branch1]	0.07
1	0.08	6	0.04	[=Branch1]	0.06
O	0.05	=	0.04	[N]	0.05
2	0.05	N	0.04	[=O]	0.03
=	0.04	n	0.02	[O]	0.03

Token	TSSA_J	TSSA_B	TSSA_M	TSSA_S
C	0.41	0.38	0.41	0.40
&^	0.28	0.15	0.24	0.13
=	0.09	0.12	0.11	0.13
1	0.08	0.08	0.08	0.07
N	0.06	0.07	0.06	0.05
O	0.04	0.05	0.04	0.05
(	0.00008	0.04346	0.00807	0.04273
)	0.00008	0.04346	0.00807	0.04273

TSDY_Brics		TSDY_MMPA		TSDY_Scaffold	
C	0.17	C	0.24	C	0.31
c	0.16	*	0.19	=	0.12
&^	0.11	&^	0.16	&^	0.10
*	0.10	=	0.09	*	0.08
1	0.08	1	0.07	(	0.07
(	0.08	(	0.06	)	0.07
)	0.08	)	0.06	1	0.05

C.2 Nesting Depth

C.2.1 Nesting Depth on ChEMBL

Supplementary Table 17 Nesting Depth on ChEMBL

Nesting Depth	SMILES	TSSA_J	TSSA_B	TSSA_M	TSSA_S	Nesting Depth	SMILES %	TSSA_S %
0	10152	1505710	65217	813353	56565	0-1-2	68.006	91.172
1	327240	39516	794554	689292	825258			
2	714157	696	544184	40459	527936			
3	389631	315	122581	2953	121767	3-4-5	31.886	8.819
4	90440	24	17621	178	13240			
5	12971		1887	26	1360			
6	1464	2	193	2	116	6-11	0.108	0.009
7	178		22		16			
8	21		4		1			
9	6				1			
10	2				2			
11	1				1			

Nesting Depth	TSDY_B	TSDY_M	TSDY_S	Nesting Depth	TSDY_B %	TSDY_M %	TSDY_S %
0	12541	14356	10235	0-1-2	89.015	99.270	77.385
1	742624	1316790	438256				
2	621247	203793	748058				
3	145790	10762	297920	3-4-5	10.971	0.730	22.573
4	21512	395	46366				
5	2346	127	4740				
6	192	3	33	6-11	0.013	0.00019	0.042
7	10		607				
8	1		3				
9			5				
10			2				
11			1				

C.2.2 Nesting Depth on Zinc

Supplementary Table 18 Nesting Depth on Zinc

Nesting Depth	SMILES	TSSA_J	TSSA_B	TSSA_M	TSSA_S	Nesting Depth	SMILES %	TSSA_B %	TSSA_S %
0	1117	248136	22143	152452	5358	0-1-2	79.8	97.3	96.7
1	62760	1319	163119	93730	169681				
2	135211	1	57482	3148	66077				
3	45789		6264	123	8041	3-4-5	20.2	2.7	3.3
4	4443		429	3	296				
5	136		19		3				

Nesting Depth	TSDY_B	TSDY_M	TSDY_S	Nesting Depth	TSDY_B %	TSDY_M %	TSDY_S %
0	1415	1516	1105	0-1-2	96.480	99.785	85.045
1	164698	229769	71281				
2	74563	17635	139764				
3	8268	530	34843	3-4-5	3.520	0.215	14.955
4	493	6	2423				
5	19		40				

C.2.3 Special Case on ChEMBL

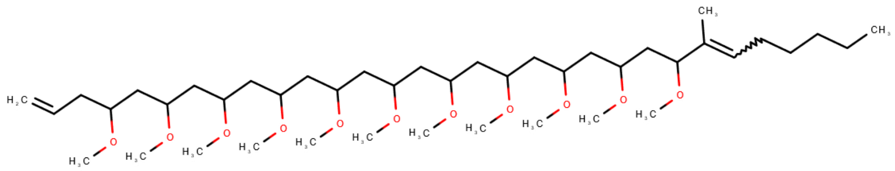

Supplementary Fig.33 The structural formula of special molecule in ChEMBL with a nesting depth of 11.

Supplementary Table 19 Different codes of molecule in ChEMBL with a nesting depth of 11.

Algorithm	Code	Length	Nesting Depth
SMILES	C=CCC(CC(CC(CC(CC(CC(CC(CC(CC(CC(CC(OC)C(C)=CCCCCC)OC)OC)OC)OC)OC)OC)OC)OC)OC)OC	80	11
DeepSmiles	C=CCCCCCCCCCCCCCCCCCCCCCCCCCOC))CC)=CCCCCC)))))))))OC))))OC))))OC))))OC))))OC))))OC))))OC))))OC))))OC))))OC	104	0
SELFIES	[C][=C][C][C][Branch2][=Branch1][#C][C][C][Branch2][=Branch1][Branch2][C][C][Branch2][Branch1][P][C][C][Branch2][Branch1][#Branch2][C][C][Branch2][Branch1][Ring1][C][C][Branch2][Ring2][N][C][C][Branch2][Ring2][Branch1][C][C][Branch2][Ring1][=C][C][C][Branch2][Ring1][#Branch1][C][C][Branch1][P][C][C][Branch1][Ring1][O][C][C][Branch1][C][C][=C][C][C][C][C][C][O][C][O][C][O][C][O][C][O][C][O][C][O][C][O][C][O][C][O][C]	419	0
TS_Vanilla	C=CCC(CC(CC(CC(CC(CC(CC(CC(CC(CC(CC(OC)C(C)=CCCCCC)OC)OC)OC)OC)OC)OC)OC)OC)OC)OC&&&	83	11
TSSA-B	C=CCCCCCCCCCCCCCCCCCCCCCCCCC&CO&CO^CO&&CO^CO&&CO^CO&&CO^CO&&CO^CO&&CO^CO&&CO^CO&&CO^CO&&CO^CO&&CO^CO&&CO^C=C&&CCCCCC&&&	118	0
TSDY-B	*C(CC(*)CC(*)CC(*)CC(*)CC(*)CC(*)CC(*)CC(*)CC(*)CC(*)CC=C)C(=*)C&*OC&&*=CCCCCC&*OC&*OC&*OC&*OC&*OC&*OC&*OC&*OC&*OC&*OC&&	120	2
TSID-B	[1*]C(CC([2*])CC([3*])CC([4*])CC([5*])CC([6*])CC([7*])CC([8*])CC([9*])CC([10*])CC([11*])CC=C)C(=[12*])C&[1*]OC&&[12*]=CCCCCC&[2*]OC&[3*]OC&[4*]OC&[5*]OC&[6*]OC&[7*]OC&[8*]OC&[9*]OC&[10*]OC&[11*]OC&&	198	2
TSSA-M	C=C&CC&CC&C&CC&CC^CO^C&CO&CC&&&CC^CO^C&CO&CC&&&CC^CO^C&CO&CO&&&CO^CC&&CC&C&CCC(CCO)OC&CC^CO^CC^CO^CO&C&&&&&CC&CC^CO^C&CO&CC&&&CC^CO^C&CO&CC&&&CC^CO^C&CO&CO&&&CO^CC&&C&CC&&C=C^CC&CC&CC&CC&CC&&&	192	1
TSDY-M	*C=C&*C*&*C(*)*&*C*&*C(*)*^*O*^*C*&*C&*C(*)*^*O*&&*O*&*C&*C^*C*&&&&*C(*)*&*C*&*C(*)CC(CC(*)O*)OC^*OC^*C*&&&*C(*)*^*C^*O*&&*OC^*C^*C*&&&&*C(*)*&*O*&*C^*C*&&*C(*)*&*C*&*C(*)*^*O*^*O*&*C&*C^*C(*)=C*&&&&*C&&*C*^*C*&*C*&*C*&*C&&&	224	1
TSID_M	[1*]C=C&[1*]C[2*]&[2*]C([3*])[4*]&[3*]C[5*]&[5*]C([6*])[7*]^[4*]O[42*]^[6*]C[8*]&[42*]C&[8*]C([9*])[10*]^[7*]O[41*]&&[10*]O[40*]&[41*]C&[40*]C^[9*]C[11*]&&&&[11*]C([12*])[13*]&[12*]C[14*]&[14*]C([15*])CC(CC([16*])O[39*])OC^[13*]OC^[16*]C[17*]&&&[17*]C([18*])[19*]^[39*]C^[19*]O[38*]&&[15*]OC^[38*]C^[18*]C[20*]&&&&[20*]C([21*])[22*]&[22*]O[37*]&[37*]C^[21*]C[23*]&&[23*]C([24*])[25*]&[24*]C[26*]&[26*]C([27*])[28*]^[25*]O[36*]^[27*]O[29*]&[36*]C&[29*]C^[28*]C([30*])=C[31*]&&&&[30*]C&&[31*]C[32*]^[32*]C[33*]&[33*]C[34*]&[34*]C[35*]&[35*]C&&&	542	1
TSSA-S	C=CCC(CC(CC(CC(CC(CC(CC(CC(CC(CC(CC(OC)C(C)=CCCCCC)OC)OC)OC)OC)OC)OC)OC)OC)OC)OC&&&	83	11
TSDY-S	C=CCC(CC(CC(CC(CC(CC(CC(CC(CC(CC(CC(OC)C(C)=CCCCCC)OC)OC)OC)OC)OC)OC)OC)OC)OC)OC&&&	83	11
TSID-S	C=CCC(CC(CC(CC(CC(CC(CC(CC(CC(CC(CC(OC)C(C)=CCCCCC)OC)OC)OC)OC)OC)OC)OC)OC)OC)OC&&&	83	11
TSSA-J	C=C&CC&CC&C&CO&CO^CC&&CC&C&CO&CO^CC&&CC&C&CO&CO^CC&&CC&C&CC&CC^CO^C&CO&CO&&&CO^CC&&CC&C&CO&CO^CC&&CC&C&CO&CO^CC&&CC&C&CC&CC^CO^C&CO&CO&&&CO^CC&&CC&C&CO&CO^CC&&CC&C&CC&C^CO^CC&CO&&C=C&&CC&CC&CC&CC&CC&&&	201	0

This molecule could not be broken down by Scaffold algorithm, but could be fragmented by other algorithms, so the nesting depth of the TS*_S is still 11, which is the same as the classical SMILES. But the nesting depth of other codes are 0,1 or 2.

C.3 Physicochemical Properties for Different Fragmentation Algorithms on ChEMBL

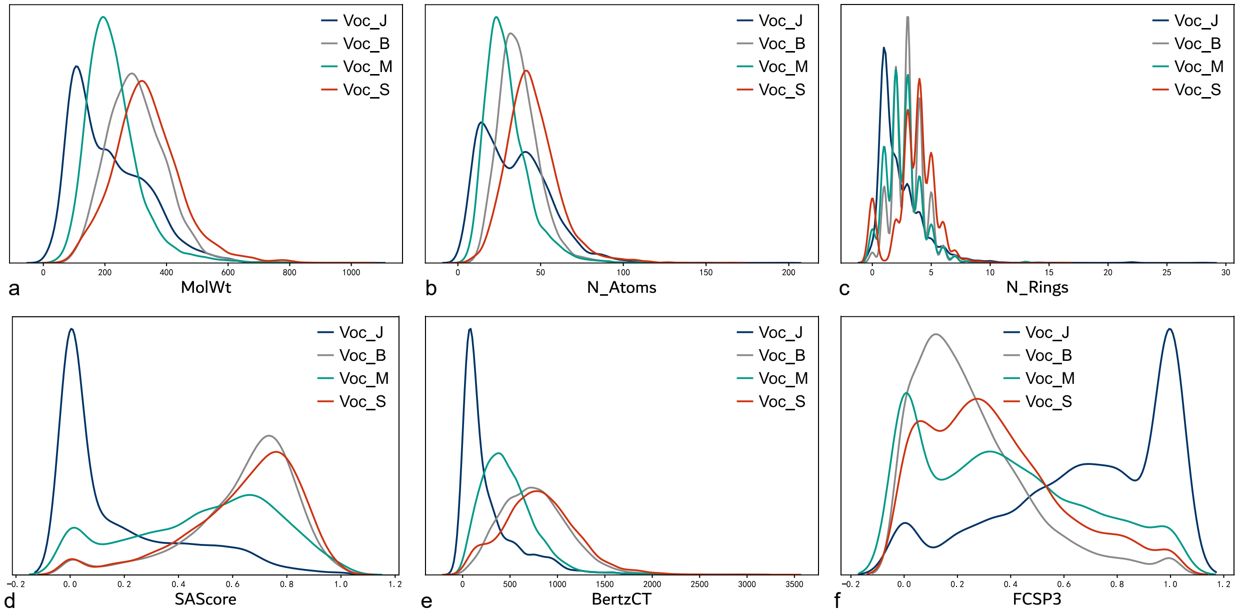

Supplementary Fig.34 Physicochemical properties for different fragmentation algorithms on ChEMBL. The unit of the y-axis is 'Density'.The figures contain labels ['Voc_J', 'Voc_B', 'Voc_M', 'Voc_S'] corresponding to the fragment's vocabulary generated by the fragmentation algorithms: JTVAE, BRICS, MMPA, Scaffold.

C.4 One Molecule and Its Reconstructed Molecules

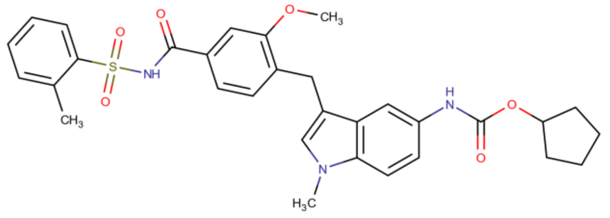

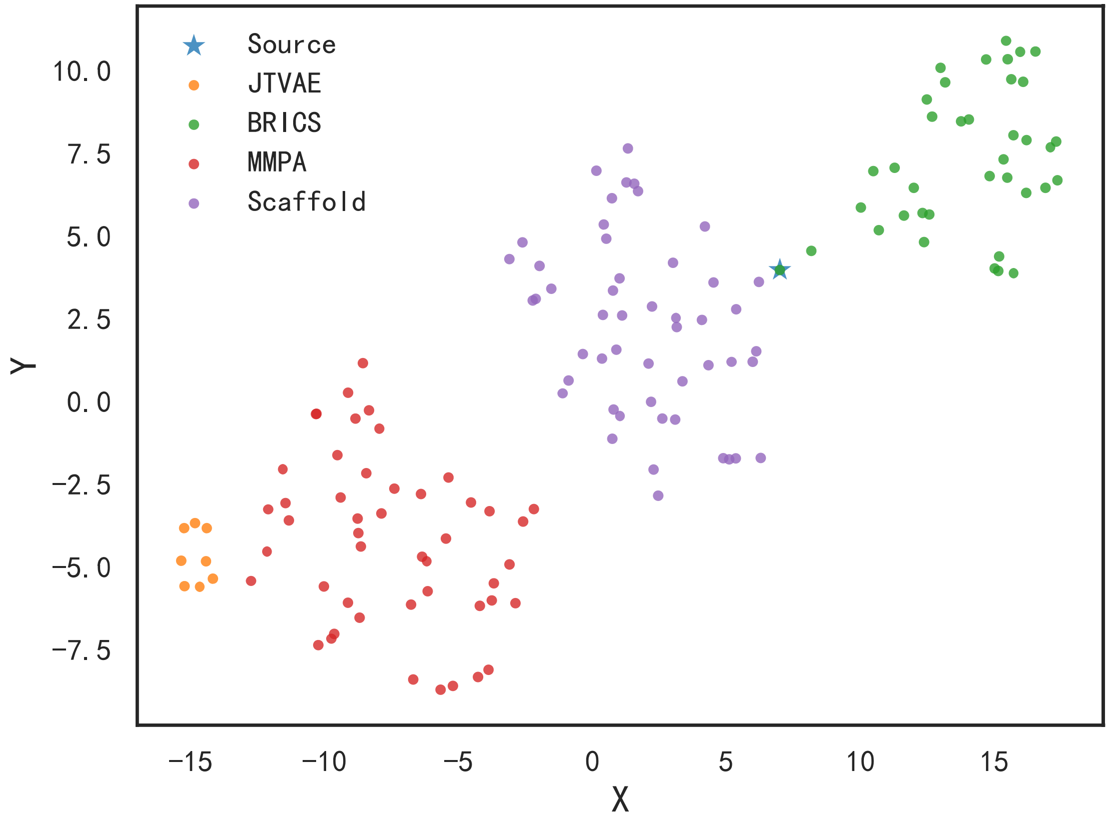

Supplementary Fig.35 t-SNE projection of one source-molecule and molecules reconstructed from JTVAE, BRICS, MMPA, Scaffold based fragmentation t-SMILES. The star and the green dot are at one point because we use a random algorithm in the reconstruction, so it is possible to reconstruct a molecule that is the same as the seed one. Topological fingerprint is used in this illustration.

D. More Experimental Results on Low-Resource Datasets

D.1 Figure of Inactive, Active, and Novel-Active Generated Molecules on JNK3

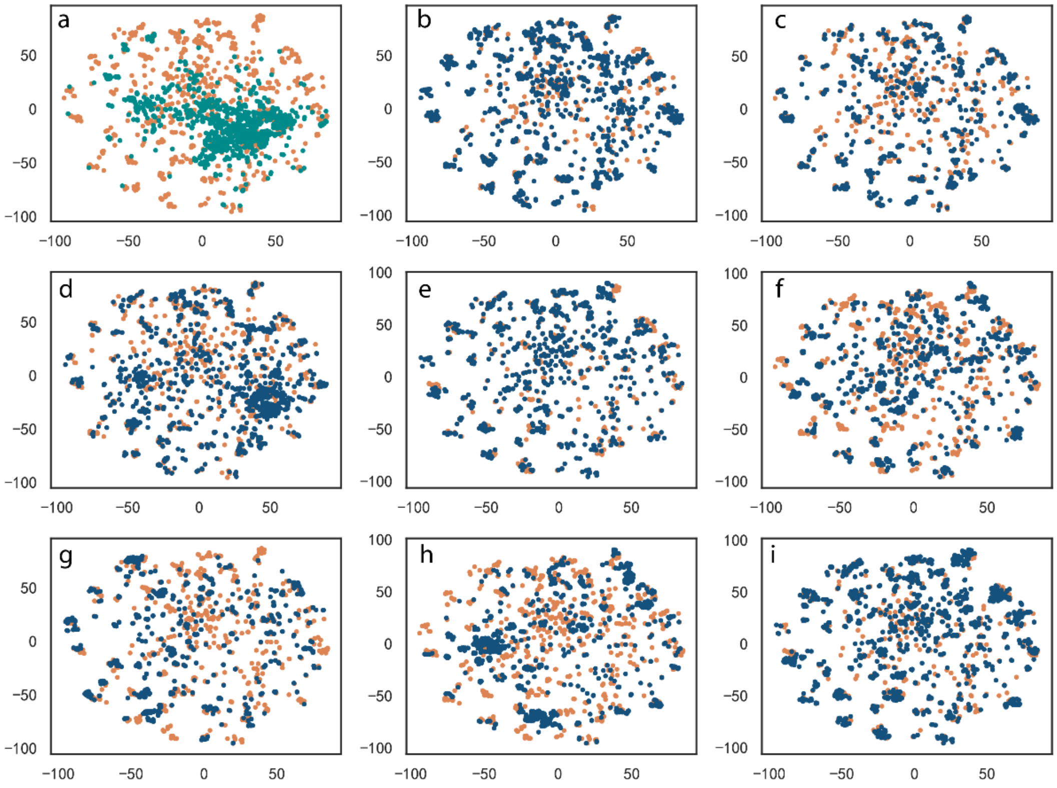

a: Inactive, b: SMILES, c:DeepSMILES, d:SELFIES, e:TSSA_S, f:TSSA_M, g:TSSA_B, h:TSSA_J
i: Pre-trained then fine-tuned model based on TSSA_S

Supplementary Fig.36 t-SNE projection of inactive molecules, active molecules, and novel active generated molecules come from SMILES, DeepSMILES, SELFIES, TSSA (Scaffold, MMPA, BRICS, JTVAE) classical models and TSSA_S based pretrain fine-tuning (Scaffold_TF) model based on JNK3 with 923 molecules. The active molecules from the training set are indicated in yellow in each panel. a: Active vs Inactive, b: Active vs SMILES, c:Active vs DeepSMILES, d: Active vs SELFIES, e: Active vs TSSA_S, f: Active vs TSSA_M, g: Active vs TSSA_B, h: Active-TSSA_J, i: Active vs pre-trained then fine-tuned model based on TSSA_S

Supplementary Fig.36 shows that newly generated active molecules cover the active molecule space, although different algorithms explore different chemical spaces.

D.2 Random Reconstruction on JNK3

Supplementary Table 20 Results of directly reconstruction using random reconstruction algorithm on JNK3.

Reconstruction	Valid	Unique	Novelty	KLD	FCD
TSSA_J[Rec1]	1.000	0.945	0.924	0.928	0.395
TSSA_B[Rec1]	1.000	0.960	0.515	0.977	0.537
TSSA_M[Rec1]	1.000	0.951	0.922	0.963	0.650
TSSA_S[Rec1]	1.000	0.997	0.859	0.950	0.692

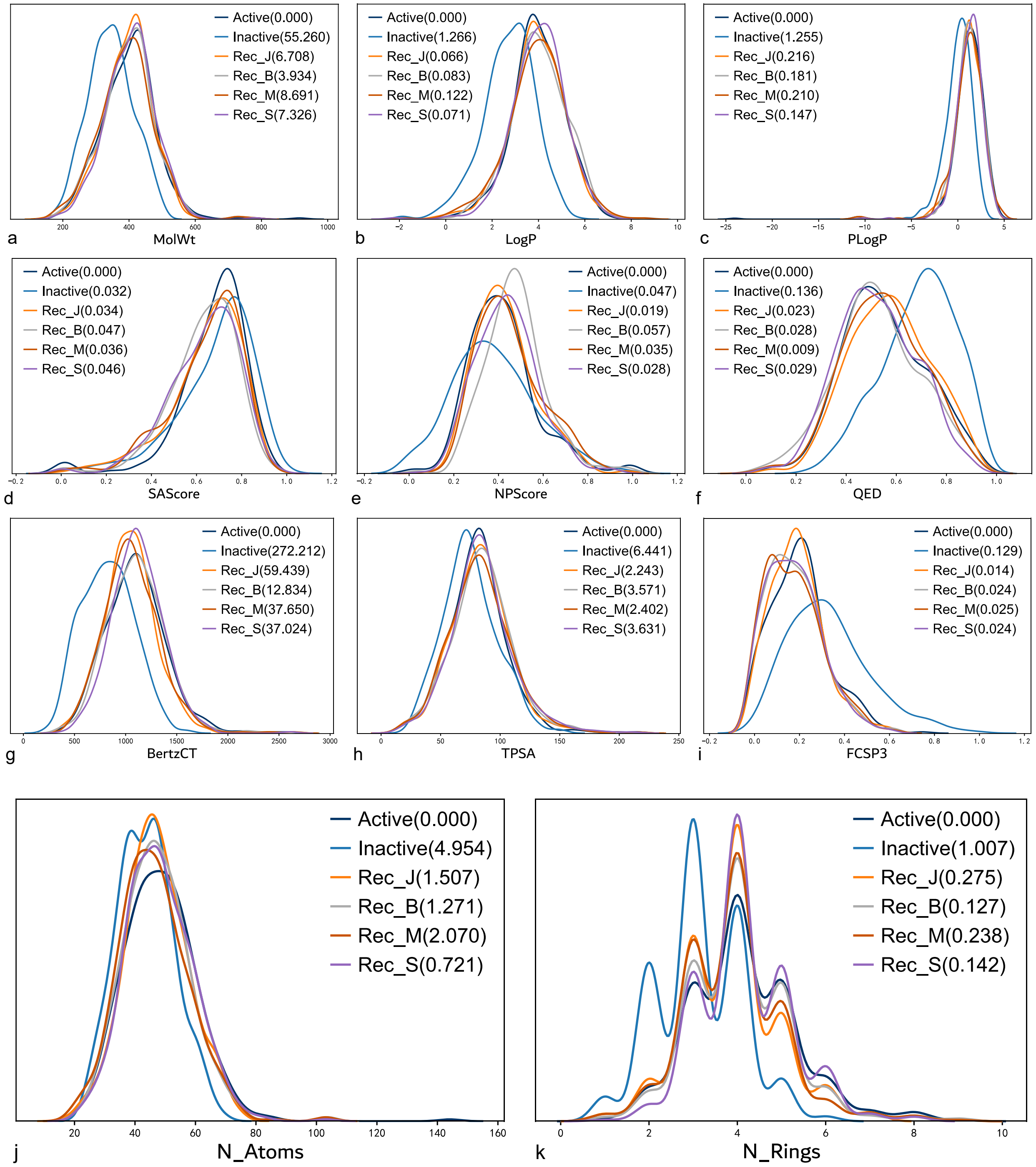

Supplementary Fig.37 Physicochemical properties of active, inactive and molecules reconstructed directly using a random algorithm on JNK3. The unit of the y-axis is 'Density'.

D.3 Distribution Learning on JNK3

Supplementary Table 21 Results of experiments on JNK3 using Singleton Models of TSSA.

Model	Valid	Unique	Novelty	KLD	FCD	%Active Novel	%FBT Novel	%Frag Novel
SMILES[R50]	0.009	0.009	**0.009**	0.333	**0.000**	0.004	N/A	N/A
SMILES[R100]	0.157	0.144	**0.119**	0.828	**0.034**	0.084	N/A	N/A
**SMILES[R200]**	0.795	0.517	**0.120**	0.971	**0.584**	**0.072**	N/A	N/A
SMILES[R300]	0.962	0.581	**0.037**	0.986	**0.704**	0.025	N/A	N/A
SMILES[R500]	0.993	0.613	**0.012**	0.988	**0.775**	0.011	N/A	N/A
SMILES[R1000]	0.995	0.613	**0.005**	0.995	**0.786**	0.004	N/A	N/A
**SMILES[R2000]**	1.000	0.600	**0.001**	0.992	**0.765**	**0.004**	N/A	N/A
DSMILES[R50]	0.009	0.009	**0.009**	0.162	**0.000**	0.002	N/A	N/A
DSMILES[R100]	0.049	0.043	**0.033**	0.688	**0.002**	0.022	N/A	N/A
**DSMILES[R200]**	0.677	0.462	**0.076**	0.966	**0.510**	**0.043**	N/A	N/A
DSMILES[R300]	0.939	0.583	**0.031**	0.987	**0.749**	0.019	N/A	N/A
DSMILES[R500]	0.988	0.610	**0.010**	0.983	**0.751**	0.008	N/A	N/A
DSMILES[R1000]	0.995	0.624	**0.004**	0.988	**0.788**	0.003	N/A	N/A
**DSMILES[R2000]**	0.999	0.593	**0.001**	0.980	**0.778**	**0.001**	N/A	N/A
SELFIES[R50]	1.000	0.975	**0.975**	0.555	**0.001**	0.496	N/A	N/A
SELFIES[R100]	1.000	0.969	**0.959**	0.639	**0.008**	0.599	N/A	N/A
**SELFIES[R200]**	1.000	0.689	**0.238**	0.944	**0.544**	**0.148**	N/A	N/A
SELFIES[R300]	1.000	0.602	**0.046**	0.990	**0.731**	0.041	N/A	N/A
SELFIES[R500]	1.000	0.627	**0.030**	0.981	**0.761**	0.064	N/A	N/A
SELFIES[R1000]	1.000	0.633	**0.014**	0.981	**0.786**	0.053	N/A	N/A
**SELFIES[R2000]**	1.000	0.614	**0.008**	0.984	**0.767**	**0.050**	N/A	N/A
TSSA_S[R50]	1.000	0.925	**0.912**	0.780	**0.125**	0.620	1.500	7.884
TSSA_S[R100]	1.000	0.963	**0.957**	0.812	**0.190**	**0.698**	2.224	12.083
TSSA_S[R200]	1.000	0.944	**0.892**	0.937	**0.495**	0.650	3.224	4.162
**TSSA_S[R300]**	1.000	0.918	**0.833**	0.934	**0.564**	**0.582**	**2.655**	**0.962**
TSSA_S[R500]	1.000	0.925	**0.835**	0.942	**0.599**	0.565	2.672	0.284
TSSA_S[R1000]	1.000	0.924	**0.821**	0.940	**0.587**	0.563	2.483	0.130
TSSA_S[R2000]	1.000	0.924	**0.830**	0.941	**0.588**	0.563	2.500	0.076
**TSSA_S[R5000]**	1.000	0.918	**0.817**	0.948	**0.608**	**0.564**	**2.534**	**0.049**
**TSSA_S[R50000]**	1.000	0.923	**0.824**	0.949	**0.572**	**0.571**	**2.379**	**0.023**
**TF_SMILES[R5]**	0.887	0.802	**0.707**	0.961	**0.523**	**0.526**	N/A	N/A
TF_SMILES[R10]	0.962	0.742	**0.404**	0.980	**0.683**	0.295	N/A	N/A
TF_SMILES[R20]	0.990	0.678	**0.181**	0.979	**0.738**	0.120	N/A	N/A
**TF_SMILES[R100]**	0.999	0.608	**0.033**	0.992	**0.764**	**0.023**	N/A	N/A
TF_SMILES[R1000]	1.000	0.600	**0.001**	0.992	**0.765**	0.004	N/A	N/A
**TF_TSSA_S[R5]**	1.000	0.960	**0.932**	0.935	**0.483**	**0.710**	**2.897**	**9.105**
TF_TSSA_S[R10]	1.000	0.927	**0.865**	0.931	**0.541**	0.658	2.121	3.613
TF_TSSA_S[R20]	1.000	0.926	**0.850**	0.944	**0.558**	0.589	2.259	1.217
**TF_TSSA_S[R100]**	1.000	0.937	**0.849**	0.932	**0.570**	**0.569**	**2.431**	**0.208**
**SMILES_Aug50[R10]**	0.807	0.727	**0.570**	0.962	**0.566**	**0.483**	N/A	N/A
SMILES_Aug50[R20]	0.919	0.735	**0.349**	0.985	**0.700**	0.300	N/A	N/A
SMILES_Aug50[R50]	0.971	0.694	**0.127**	0.989	**0.754**	0.100	N/A	N/A
**SMILES_Aug50[R100]**	0.995	0.649	**0.049**	0.989	**0.750**	**0.047**	N/A	N/A
SMILES_Aug50[R200]	0.998	0.631	**0.026**	0.990	**0.783**	0.021	N/A	N/A
**TSSA_S_Rec50[R10]**	1.000	0.970	**0.962**	0.893	**0.389**	**0.829**	**2.414**	**1.757**
TSSA_S_Rec50[R50]	1.000	0.977	**0.954**	0.889	**0.401**	0.817	2.397	0.722
**TSSA_S_Rec50[R100]**	1.000	0.975	**0.960**	0.894	**0.411**	**0.809**	**2.448**	**0.655**
TSSA_J [R500]	1.000	0.811	**0.801**	0.910	**0.304**	0.276	1.780	0.416
TSSA_J [R1000]	1.000	0.795	**0.779**	0.919	**0.309**	0.266	1.602	0.131
TSSA_B[R800]	1.000	0.681	**0.415**	0.959	**0.406**	0.174	0.913	0.157
TSSA_B[R1000]	1.000	0.716	**0.428**	0.955	**0.435**	0.149	0.783	0.132
TSSA_M[R500]	1.000	0.796	**0.769**	0.959	**0.508**	0.287	2.356	0.236
TSSA_M[R1000]	1.000	0.790	**0.771**	0.966	**0.533**	0.278	2.243	0.131

D.4 Distribution Learning on JNK3 for TSDY and Hybrid Models of TSSA

Supplementary Table 22 Results of experiments on JNK3 with Hybrid Models of TSSA. TSSA_H6 represents the hybrid of the following: TS_Vanilla, TSSA_J, TSSA_B, TSSA_M, TSSA_S, and TSSA_BRICS_Adv.

Model	Valid	Unique	Novelty	KLD	FCD	%Active Novel	%FBT Novel	%Frag Novel
TSSA_HSV[R50]	1.000	0.901	**0.869**	0.819	**0.155**	0.506	1.879	54.905
TSSA_HSV[R100]	1.000	0.893	**0.756**	0.944	**0.466**	0.538	2.000	40.766
**TSSA_HSV[R200]**	1.000	0.827	**0.483**	0.961	**0.680**	**0.350**	**2.086**	**5.044**
TSSA_HSV[R300]	1.000	0.843	**0.431**	0.965	**0.720**	0.322	1.862	2.336
TSSA_HSV[R500]	1.000	0.835	**0.419**	0.972	**0.727**	0.322	2.034	1.161
TSSA_HSV[R1000]	1.000	0.839	**0.437**	0.970	**0.683**	0.322	1.759	0.591
**TSSA_HSV[R2000]**	1.000	0.844	**0.447**	0.966	**0.716**	**0.319**	**1.810**	**0.365**
TSSA_H6[R50]	1.000	0.912	**0.849**	0.864	**0.306**	0.521	2.810	66.438
TSSA_H6[R100]	1.000	0.887	**0.677**	0.937	**0.579**	0.409	2.897	32.474
**TSSA_H6[R200]**	1.000	0.896	**0.683**	0.947	**0.622**	**0.374**	**2.310**	**25.978**
TSSA_H6[R300]	1.000	0.879	**0.647**	0.959	**0.640**	0.381	2.362	24.891
TSSA_H6[R500]	1.000	0.883	**0.652**	0.952	**0.631**	0.375	2.483	24.394
TSSA_H6[R1000]	1.000	0.894	**0.658**	0.958	**0.633**	0.377	2.672	24.044
**TSSA_H6[R2000]**	1.000	0.887	**0.657**	0.949	**0.619**	**0.437**	**2.672**	**23.745**

Supplementary Table 23 Results of experiments on JNK3 with TSDY.

Model	Valid	Unique	Novelty	KLD	FCD	%Active Novel	%FBT Novel	%Frag Novel
TSDY_B[R500]	1.000	0.645	**0.118**	0.985	**0.695**	0.033	0.409	0.564
TSDY_B[R1000]	1.000	0.634	**0.092**	0.985	**0.713**	0.019	0.545	0.291
TSDY_M[R500]	1.000	0.862	**0.740**	0.969	**0.580**	**0.275**	1.322	0.865
TSDY_M[R1000]	1.000	1.000	**0.732**	0.968	**0.617**	**0.263**	1.231	0.724
TSDY_S[R500]	1.000	0.748	**0.389**	0.988	**0.698**	0.109	0.231	0.408
TSDY_S[R1000]	1.000	0.726	**0.368**	0.977	**0.687**	0.098	0.154	0.163

D.5 Novelty and FCD Curves on JNK3 with TSSA

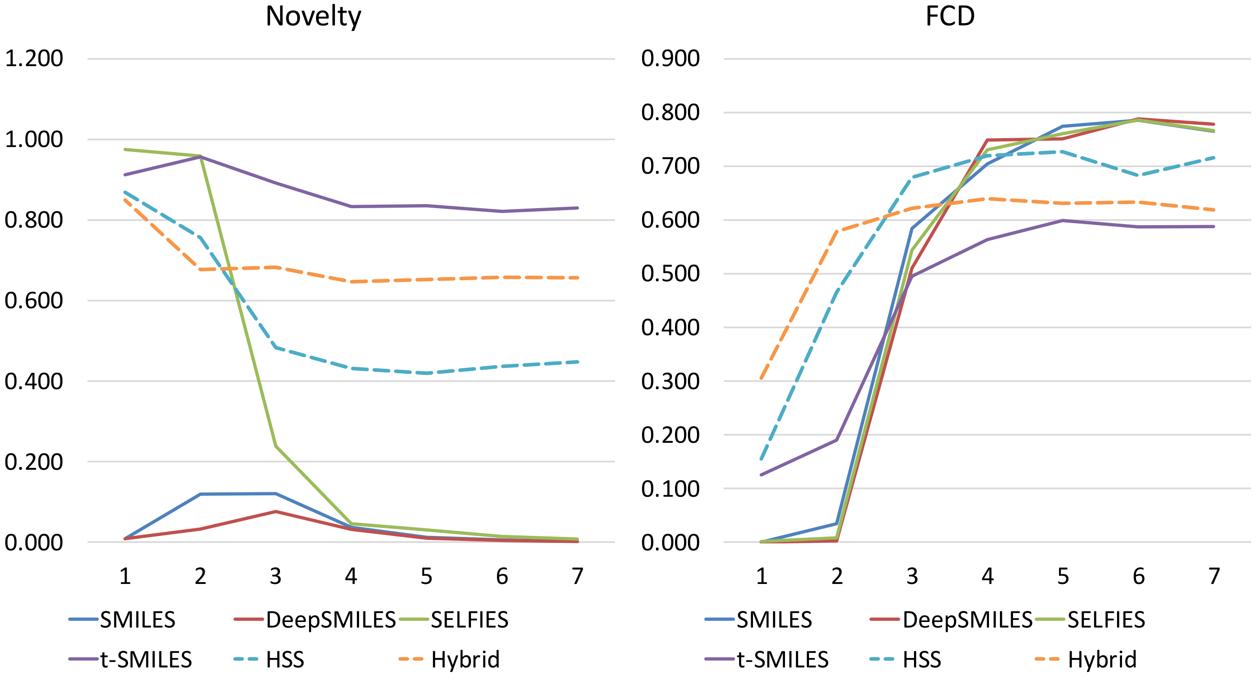

Supplementary Fig.38 Novelty and FCD scores against SMILES, DSMILES, SELFIES, TSSA, HSS(Hybrid model on Vanilla and Scaffold) and Hybrid(Hybrid model on Vanilla, JTVAE, BRICS, MMPA and Scaffold) on different training epochs. The numbers 1, 2, 3, 4, 5, 6, and 7 denote 50, 100, 200, 300, 500, 1K, and 2K training epochs.

D.6 Physicochemical Properties on JNK3

D.6.1 Baseline Models on JNK3

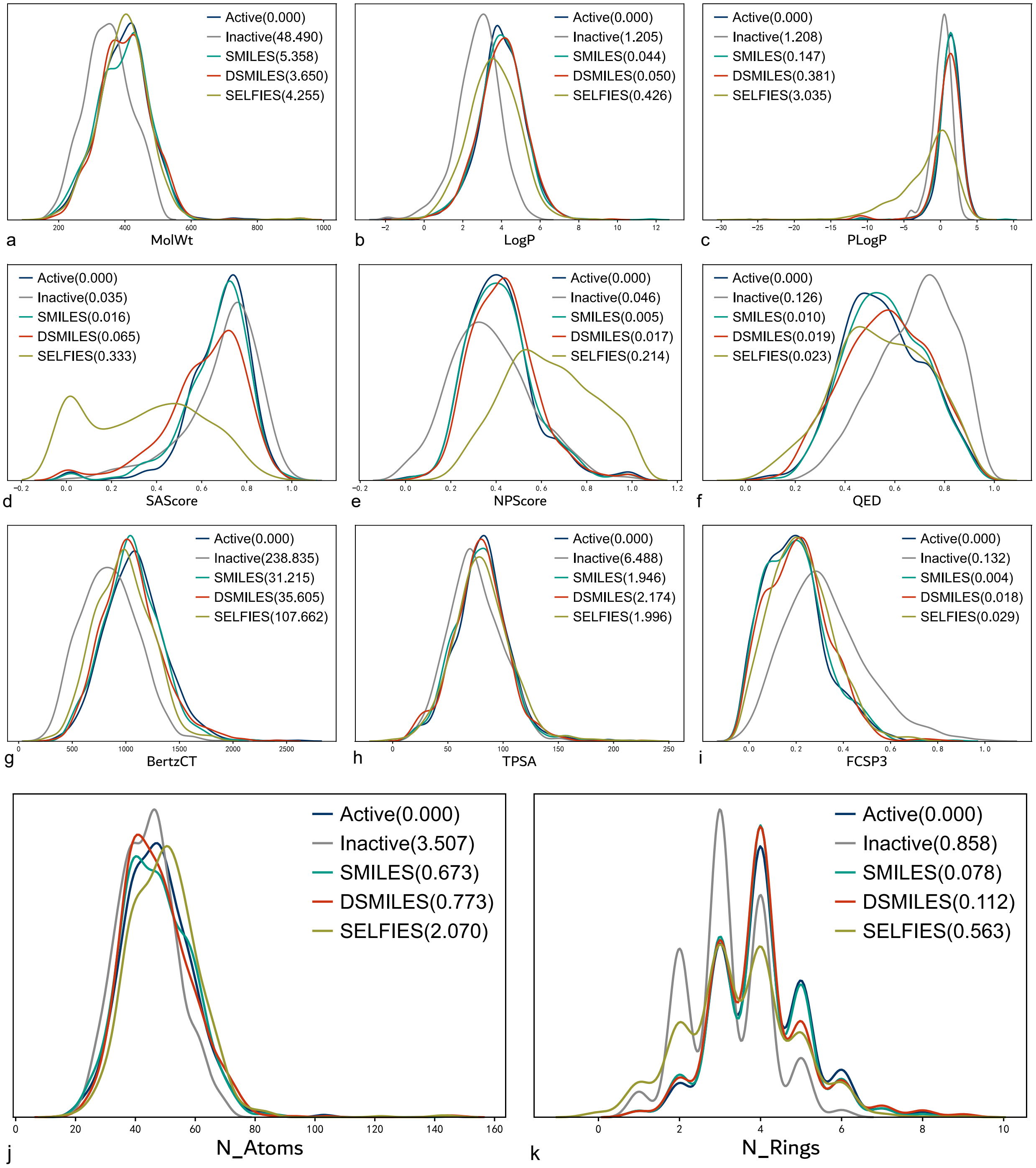

Supplementary Fig.39 Physicochemical properties of active, inactive and generated molecules by SMILES, DSMILES, SELFIES models on JNK3. The unit of the y-axis is 'Density'.

D.6.2 t-SMILES Models on JNK3

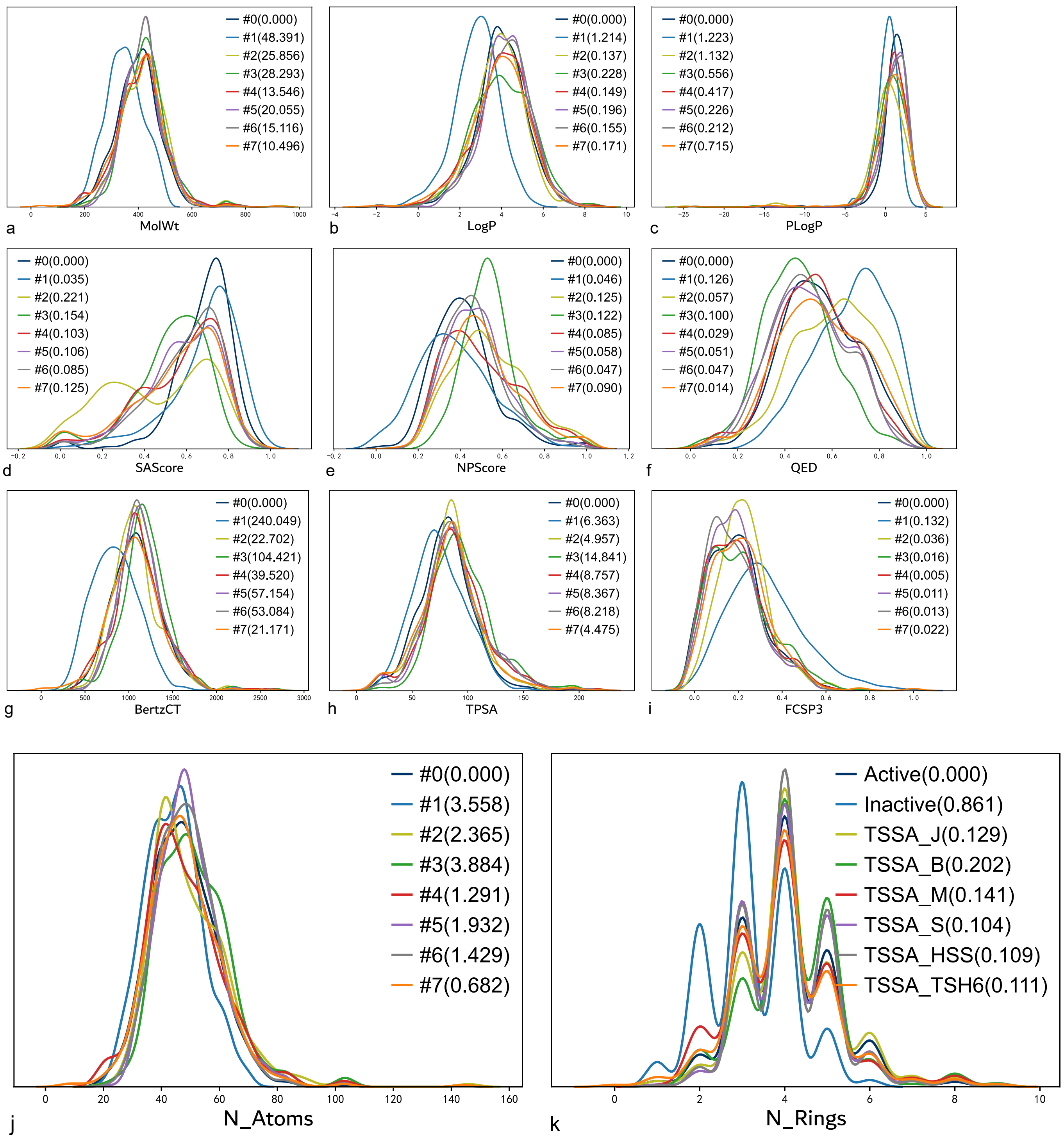

Supplementary Fig.40 Physicochemical properties of active, inactive and generated molecules by different TSSA models on JNK3. The unit of the y-axis is 'Density'.The figures contain labels ['#0', '#1', '#2', '#3', '#4', '#5', '#6', '#7'] corresponding to the following datasets: ['Active', 'Inactive', 'TSSA_J', 'TSSA_B', 'TSSA_M', 'TSSA_S', 'TSSA_HSS', 'TSSA_TSH6']. TSH6 represents the hybrid of the following: TS_Vanilla, TSSA_J, TSSA_B, TSSA_M, TSSA_S, and TSSA_BRICS_Adv.

D.7 Different Training Epochs for t-SMILES and SELFIES on JNK3

The difference between SELFIES and t-SMILES, which can theoretically generate 100% valid molecules, can be seen from the below generated molecules. These molecules were cluster centers of 10000 randomly selected molecules using the Ward algorithm based on MACCS fingerprints.

The SELFIES-based model generates more "big ring" (FCD scores are: 0.001, 0.008) molecules in the 50th and 100th training epoch, while the Scaffold-based t-SMILES model generates more smaller molecules (FCD scores are: 0.125, 0.190).

This reveals a tendency for SELFIES to create large rings in "valid" chemical graphs, leading to poor synthetic accessibility. Please refer SI.D.6.1 for SAScores.

D.7.1 Training Data [Active Molecules]

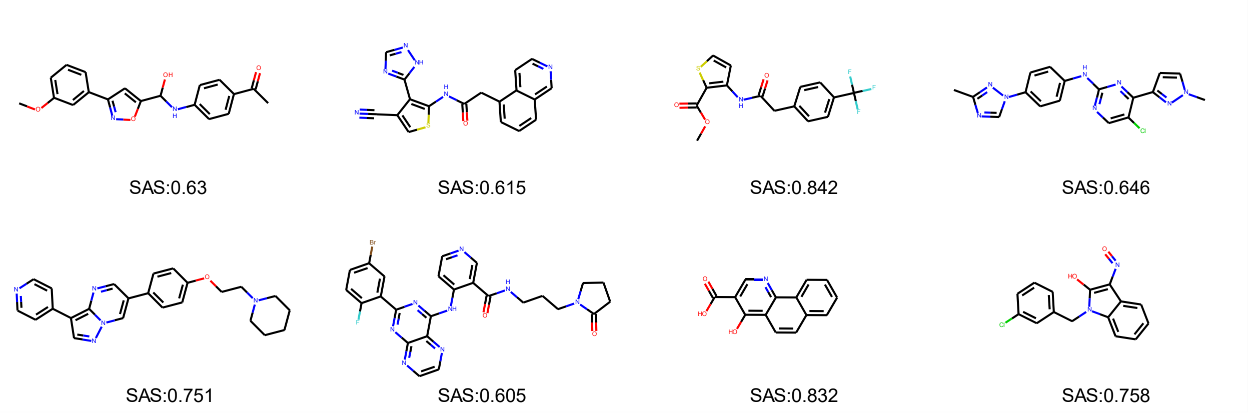

Supplementary Fig.41 Randomly selected molecules from the training dataset which are all active on JNK3.

D.7.2 TSSA_S[Rnd50]

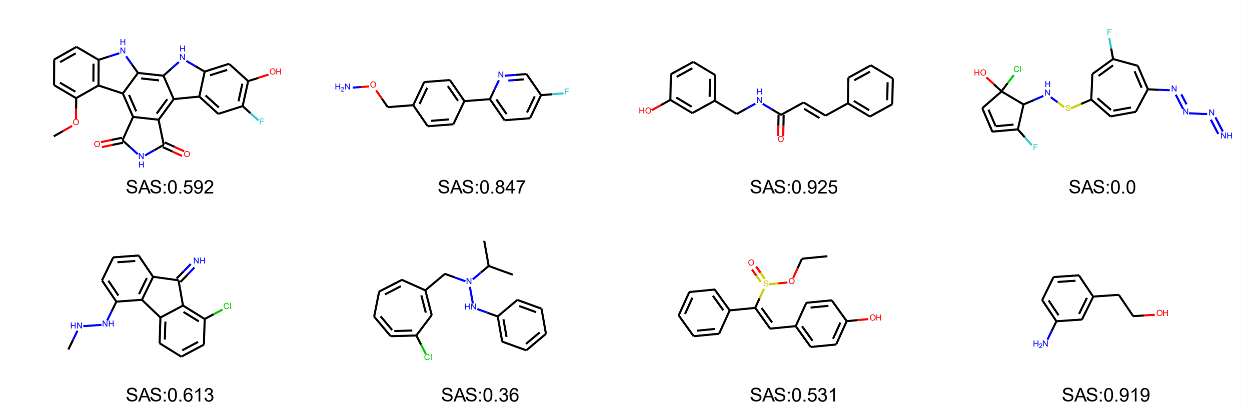

Supplementary Fig.42 Generated molecules by TSSA_S model with training round is 50 on JNK3.

D.7.3 TSSA_S[Rnd100]

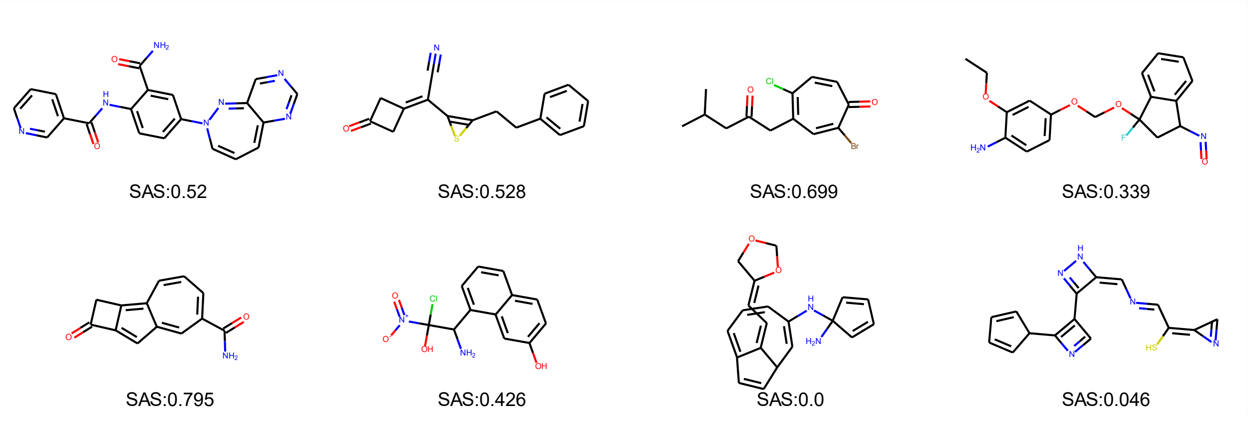

Supplementary Fig.43 Generated molecules by TSSA_S model with training round is 100 on JNK3.

D.7.4 TSSA_S[Rnd300]

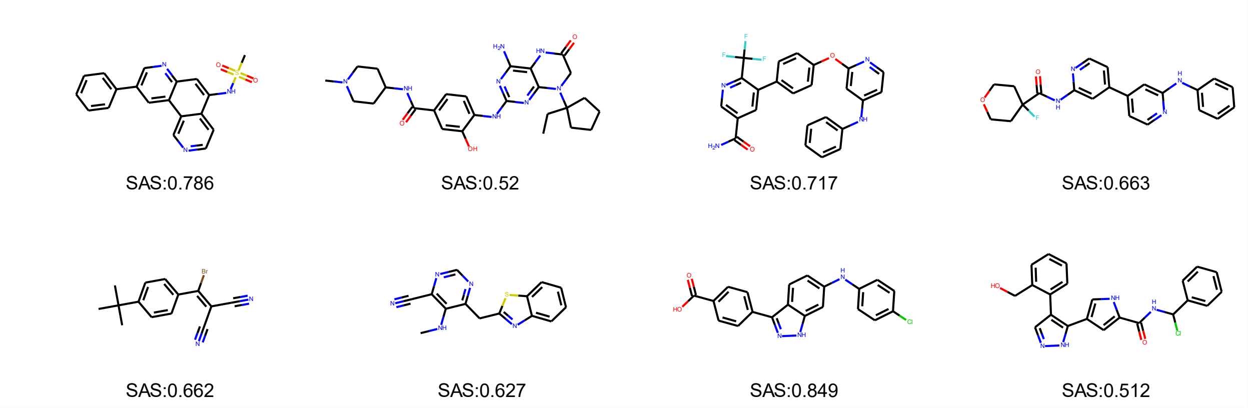

Supplementary Fig.44 Generated molecules by TSSA_S model with training round is 300 on JNK3.

D.7.5 TSSA_S[Rnd5000]

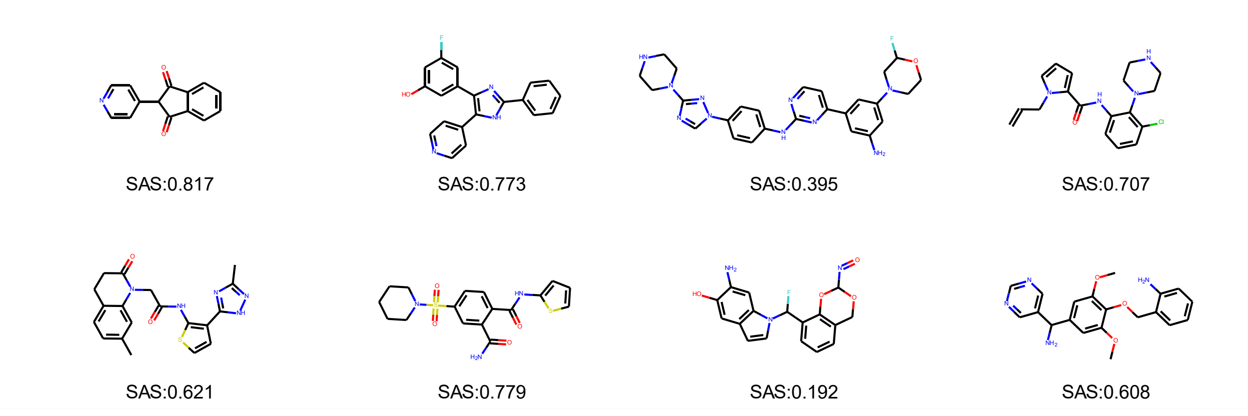

Supplementary Fig.45 Generated molecules by TSSA_S model with training round is 5000 on JNK3.

D.7.6 SELFIES[Rnd50]

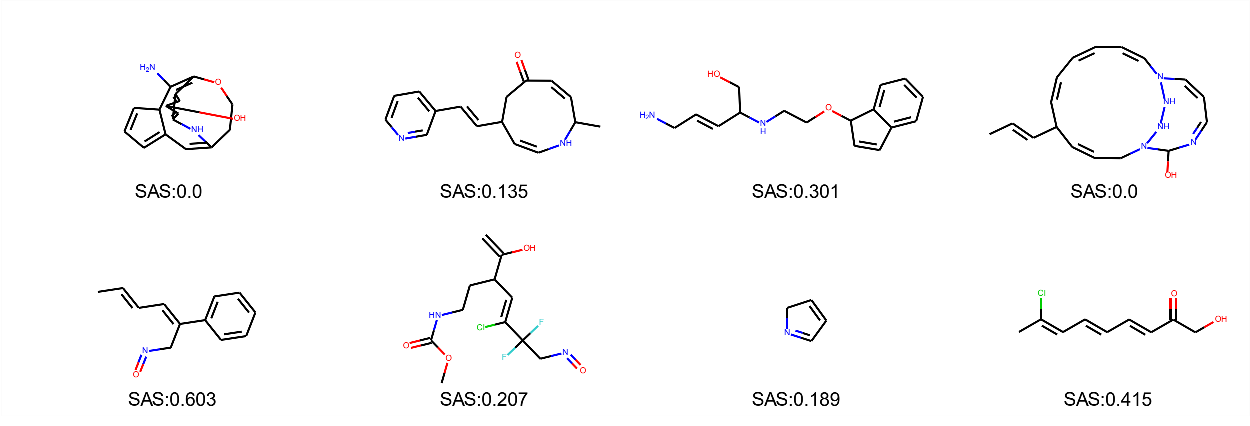

Supplementary Fig.46 Generated molecules by SELFIES model with training round is 50 on JNK3.

D.7.7 SELFIES[Rnd100]

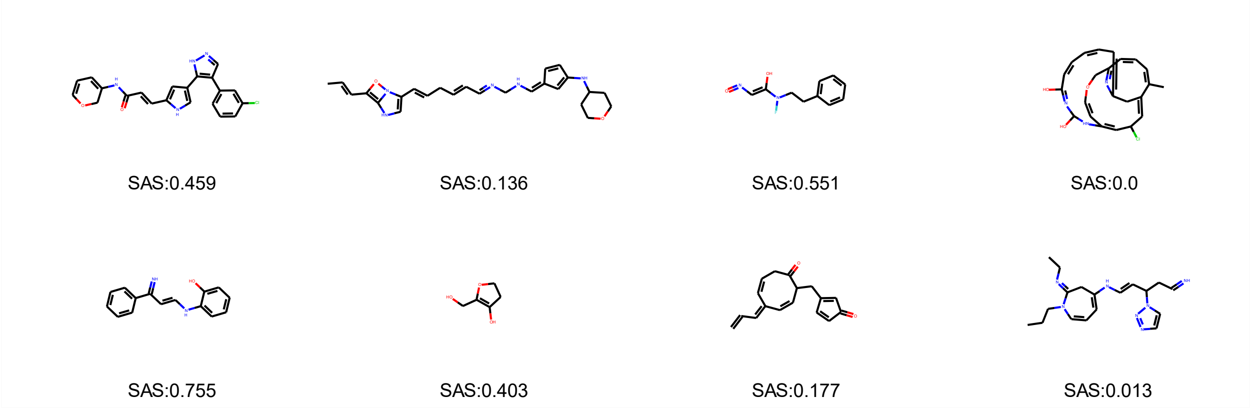

Supplementary Fig.47 Generated molecules by SELFIES model with training round is 100 on JNK3.

D.7.8 SELFIES[Rnd200]

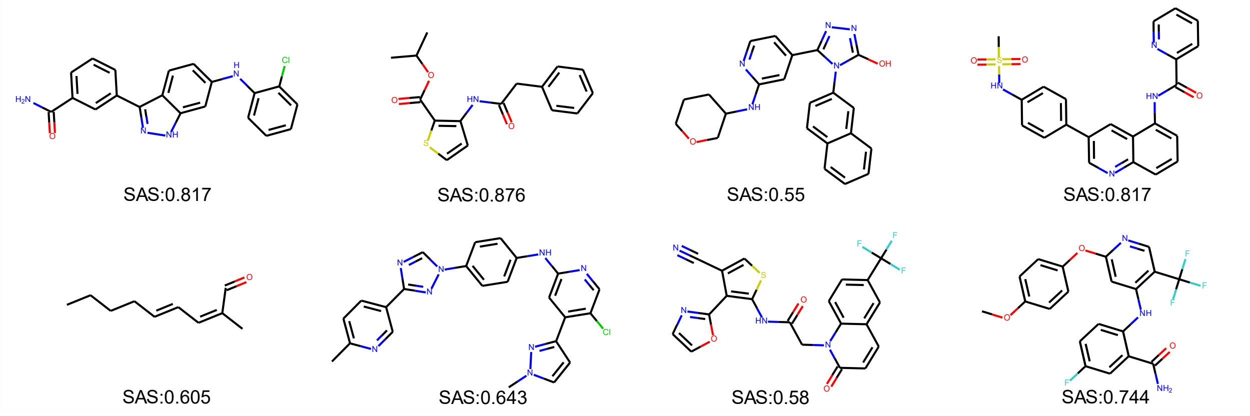

Supplementary Fig.48 Generated molecules by SELFIES model with training round is 200 on JNK3.

D.7.9 SELFIES[Rnd1000]

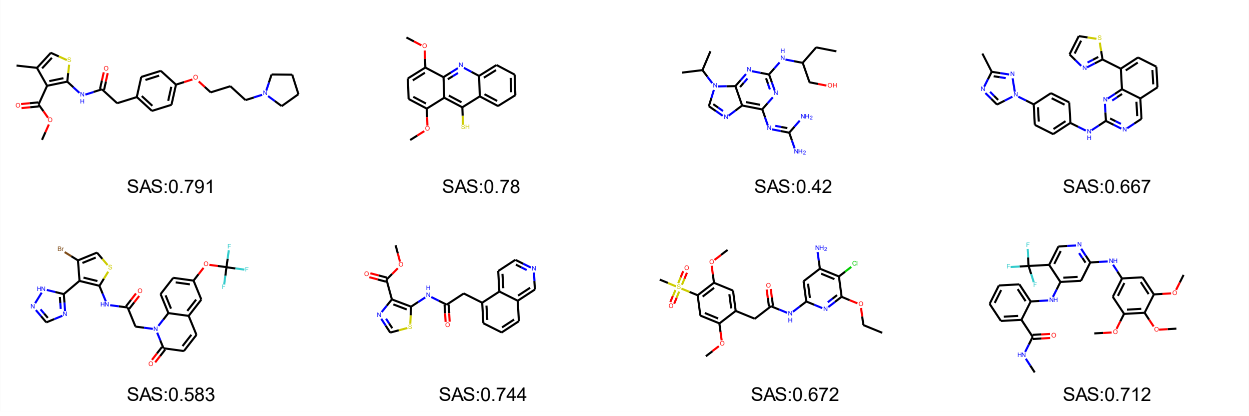

Supplementary Fig.49 Generated molecules by SELFIES model with training round 1000 on JNK3.

D.8 Ablation Study on JNK3

Splitting a molecule into fragments to generate the t-SMILES string and reconstructing the newly generated string into a valid molecule are two key steps in the overall t-SMILES solution. We carry out an ablation study to explore a scenario on JNK3. We train the generative model using classical SMILES and then decompose and reconstruct the generated molecules. The results can be found in Supplementary Table 24.

Supplementary Table 24 Results for ablation study on JNK3. SSR means: Decomposition and reconstruction of newly generated molecules by a SMILES-based generative model using the Scaffold algorithm.

MolGPT[SH]	Valid	Novelty	FCD	%Active Novel	%FBT Novel	%Frag Novel
SMILES[R200]->Scaffold->Recon(SSR)	0.774	0.645	0.500	0.451	1.638	3.236
TSSA_S[R300]	1.000	0.833	0.564	0.582	2.655	0.962

Table illustrates that the validity score of the SSR model remains lower than that of the t-SMILES model, primarily because the SMILES model could not generate 100% valid molecules while balancing FCD and novelty scores, particularly on low-resource datasets. The t-SMILES model achieves higher scores for all other metrics, even though there are more novel fragments for the SSR model.

D.9 Distribution Learning on AID1706: 329 Active Molecules

To investigate whether t-SMILES based model still works well on smaller datasets. A quick experiment is performed on AID1706, which contains only 329 active molecules. Supplementary Table 25 shows that when selecting similar FCD scores, the models based on TSSA_S obtain significantly higher novelty scores, which means that the t-SMILES based generation model could effectively explore active molecule space to generate more novel active molecules even on a very small training data.

Supplementary Table 25 Distributional results on AID1706 using GPT.

Models	Valid	Uniq(↑)	Novel(↑)	KLD(↑)	FCD(↑)
SMILES[R500]	0.973	0.602	**0.024**	0.985	**0.291**
SMILES_Aug50[R 50]	0.945	0.717	0.228	0.979	0.272
TSSA_S[R1000]	1.000	0.948	**0.903**	0.947	**0.257**
TSSA_S_Rec50[R200]	1.000	0.982	0.964	0.897	0.042

D.10 Physicochemical Properties on AID1706

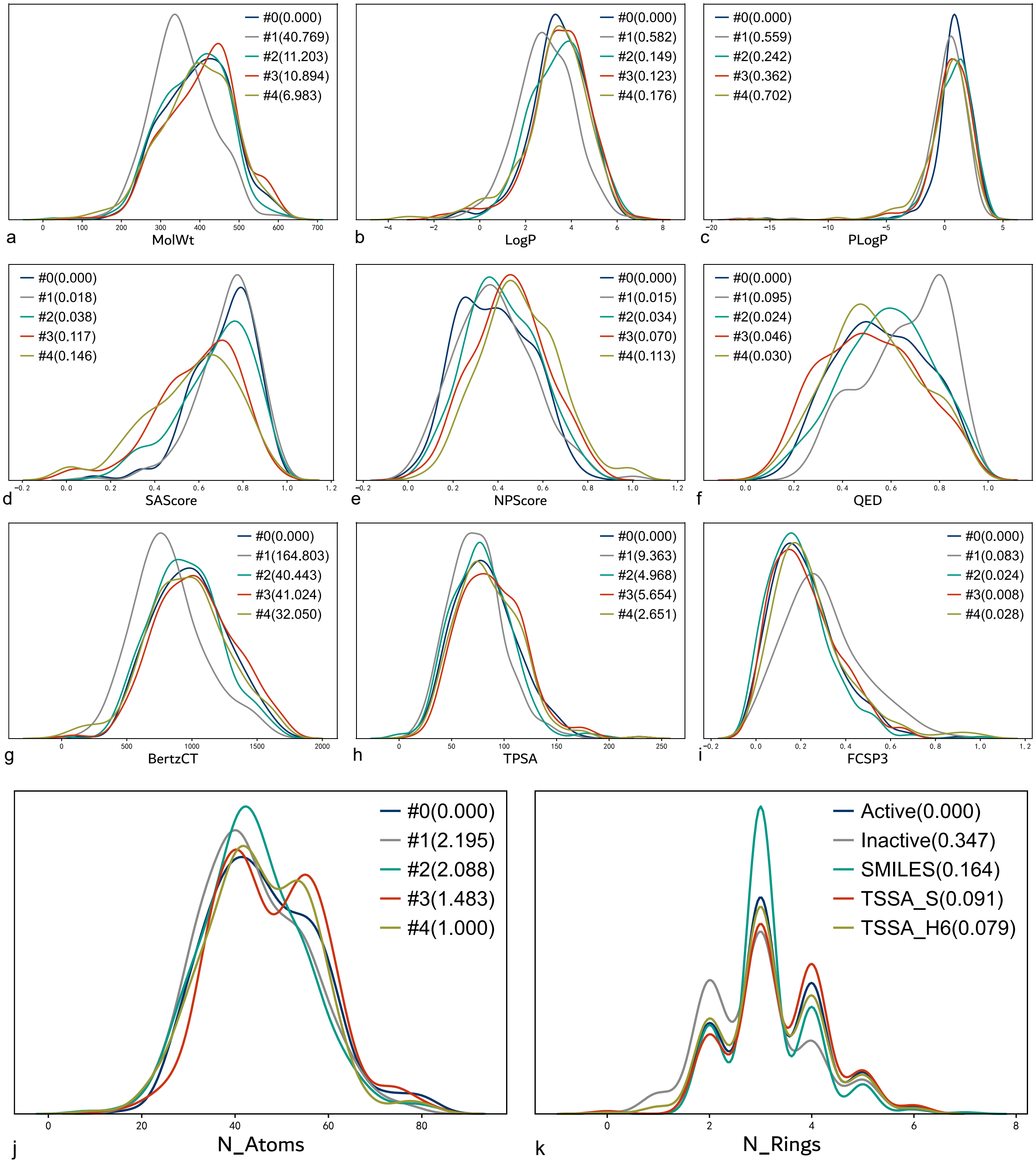

Supplementary Fig.50 Physicochemical properties of active, inactive and generated molecules by different models on AID1706. The unit of the y-axis is 'Density'.The figures contain labels ['#0', '#1', '#2', '#3', '#4'] corresponding to the following datasets: ['Active', 'Inactive', 'SMILES','TSSA_S','TSSA_H6']. TSSA_H6 represents the hybrid of the following: TS_Vanilla, TSSA_J, TSSA_B, TSSA_M, TSSA_S, and TSSA_BRICS_Adv.

D.11 Figure on AID1706

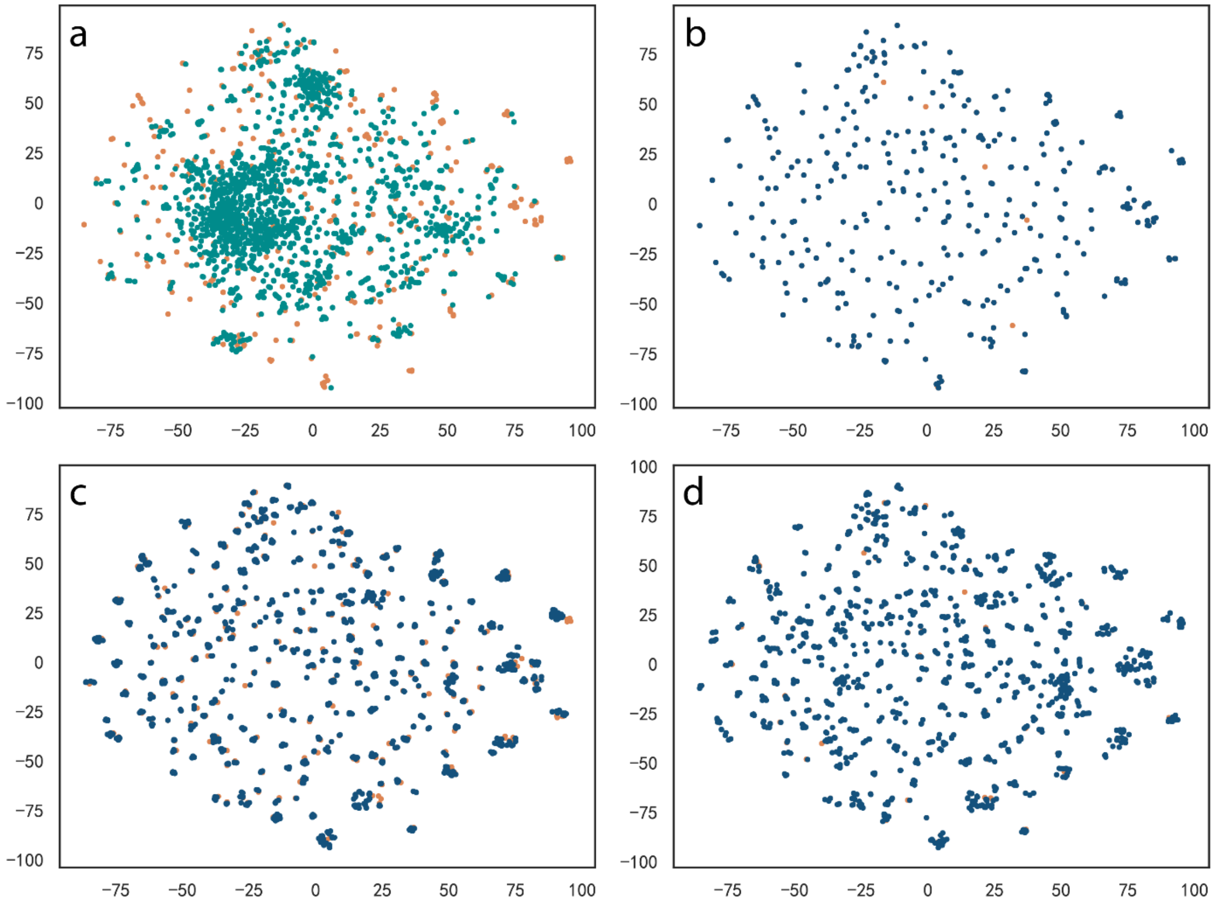

a: Active vs Inactive, b: Active vs SMILES, c: Active vs TSSA_S, d:Active vs H6

Supplementary Fig.51 t-SNE projection of inactive, active, and novel active generated molecules come from SMILES, TSSA_S and hybrid TSSA models on AID1706. The active molecules from the training set are indicated in yellow in each panel. H6 represents the hybrid of the following: TS_Vanilla, TSSA_J, TSSA_B, TSSA_M, TSSA_S, and TSSA_BRICS_Adv.

E. More Experimental Results on ChEMBL, Zinc and QM9

E.1 Random Reconstruction on ChEMBL

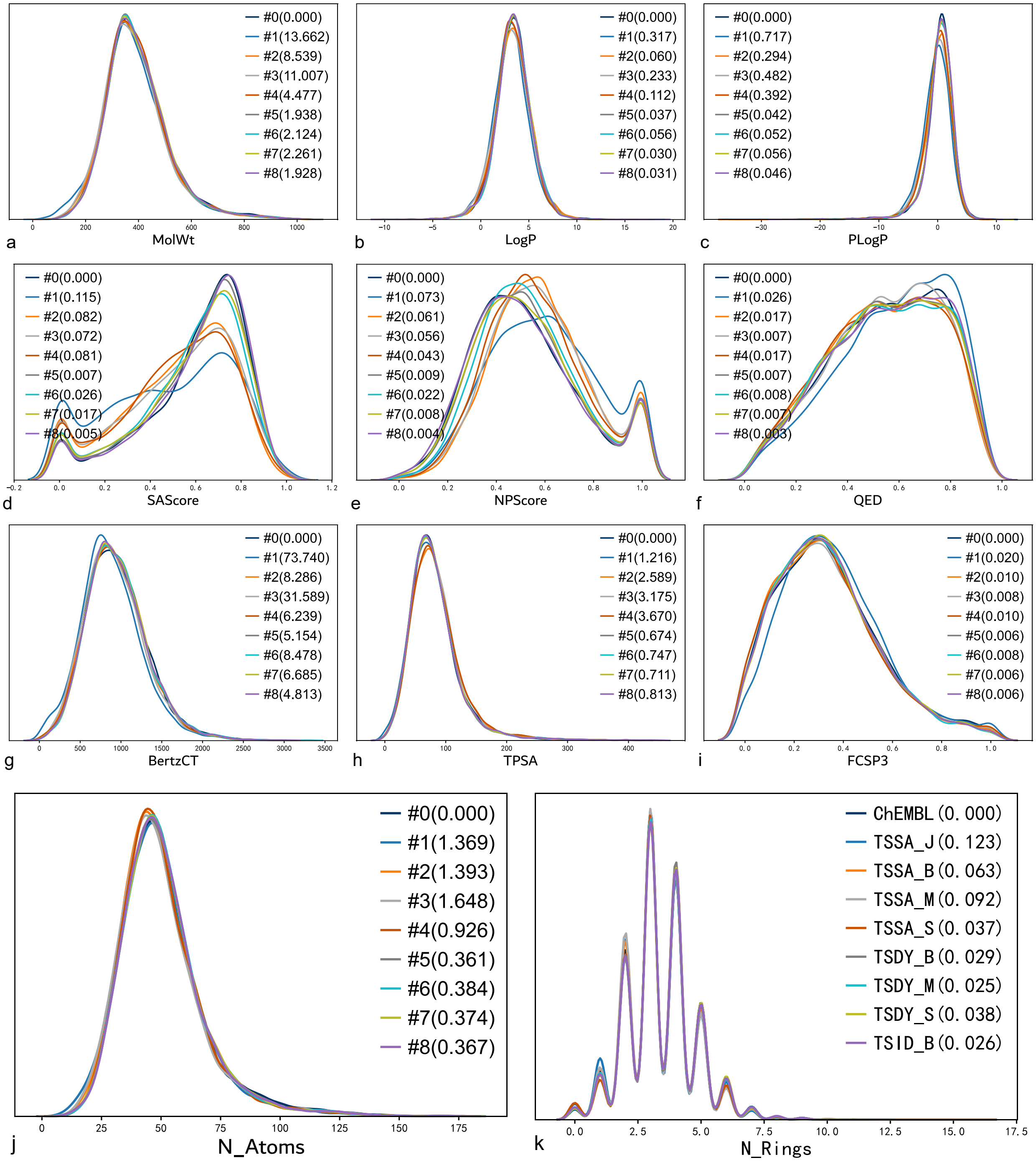

Supplementary Fig.52 Physicochemical properties of randomly reconstructed molecules on ChEMBL. The unit of the y-axis is 'Density'. The figures contain labels ['#0','#1', '#2', '#3', '#4', '#5', '#6', '#7', '#8'] corresponding to the following datasets: ['ChEMBL','TSSA_J', 'TSSA_B', 'TSSA_M', 'TSSA_S', 'TSDY_B', 'TSDY_M', 'TSDY_S', 'TSID_B']

If the definition of "recovery rate" is whether a SMILES/t-SMILES pair is exactly recovered. Supplementary Table 26 is the detailed result on ChEMBL.

$$recovery\ rate = \frac{exactly\ same\ canonicalized\ SMILES}{10K\ samples}$$

$$recovery\ rate \approx 1 - \ novelty$$

The small error arises from two factors: 1) the samples were randomly selected, and 2) t-SMILES A can be reconstructed into SMILES B due to the presence of closely related molecules in training data.

Supplementary Table 26 Recovery rate of random reconstruction on 10K samples.

Dataset	Frag Alg	Recovery Rate
ChEMBL	TSSA_J	0.122
	TSSA_B	0.307
	TSSA_M	0.114
	TSSA_S	0.104
	TSDY_B	0.781
	TSDY_M	0.275
	TSDY_S	0.541
	TSID_B	0.997
	TSID_M	0.997
	TSID_S	0.997

E.2 Random Reconstruction on Zinc

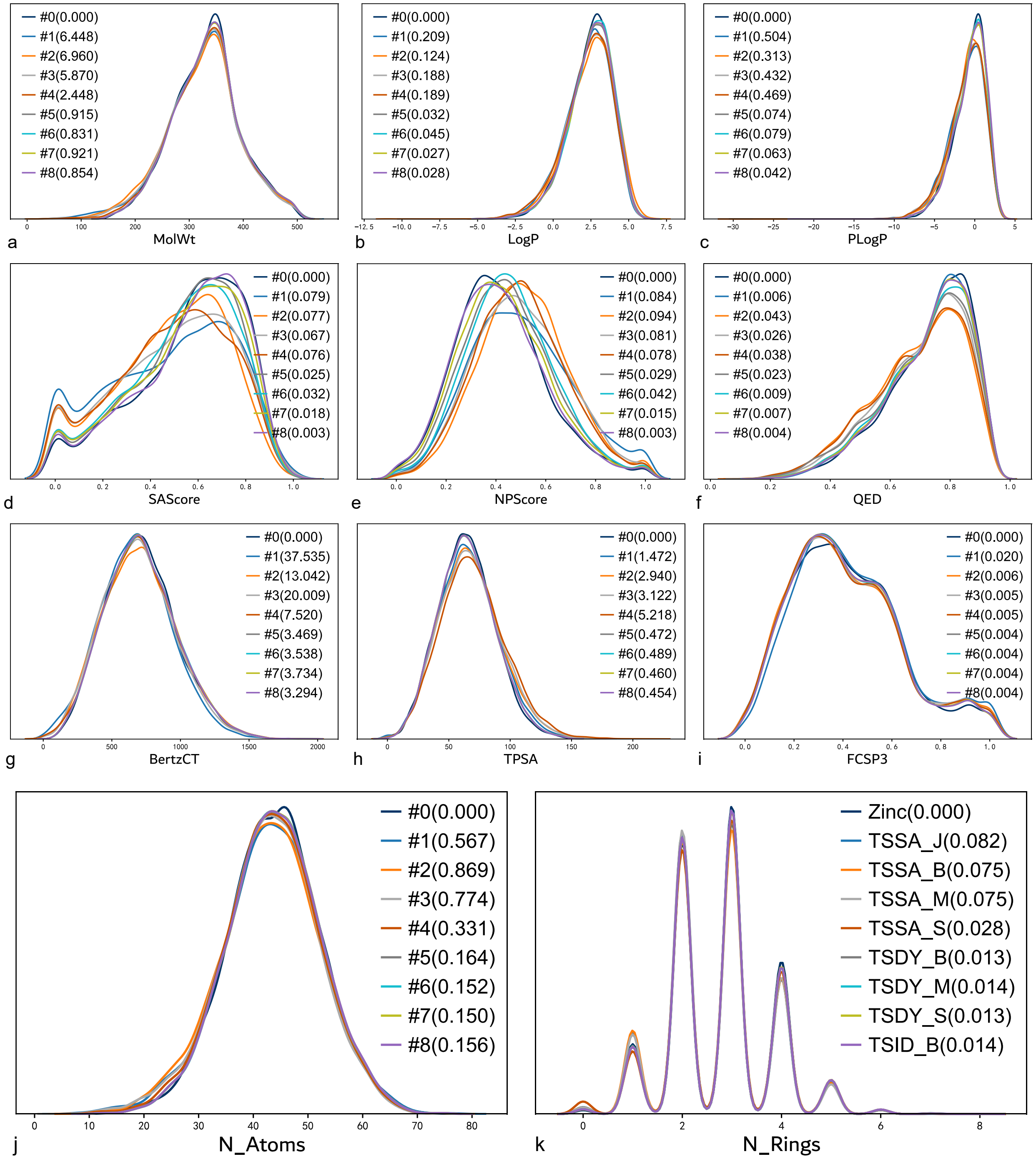

Supplementary Fig.53 Physicochemical properties of randomly reconstructed molecules on Zinc. The unit of the y-axis is 'Density'. The figures contain labels ['#0','#1', '#2', '#3', '#4', '#5', '#6', '#7', '#8'] corresponding to the following datasets: ['Zinc', 'TSSA_J', 'TSSA_B', 'TSSA_M', 'TSSA_S', 'TSDY_B', 'TSDY_M', 'TSDY_S', 'TSID_B']

E.3 Random Reconstruction on QM9

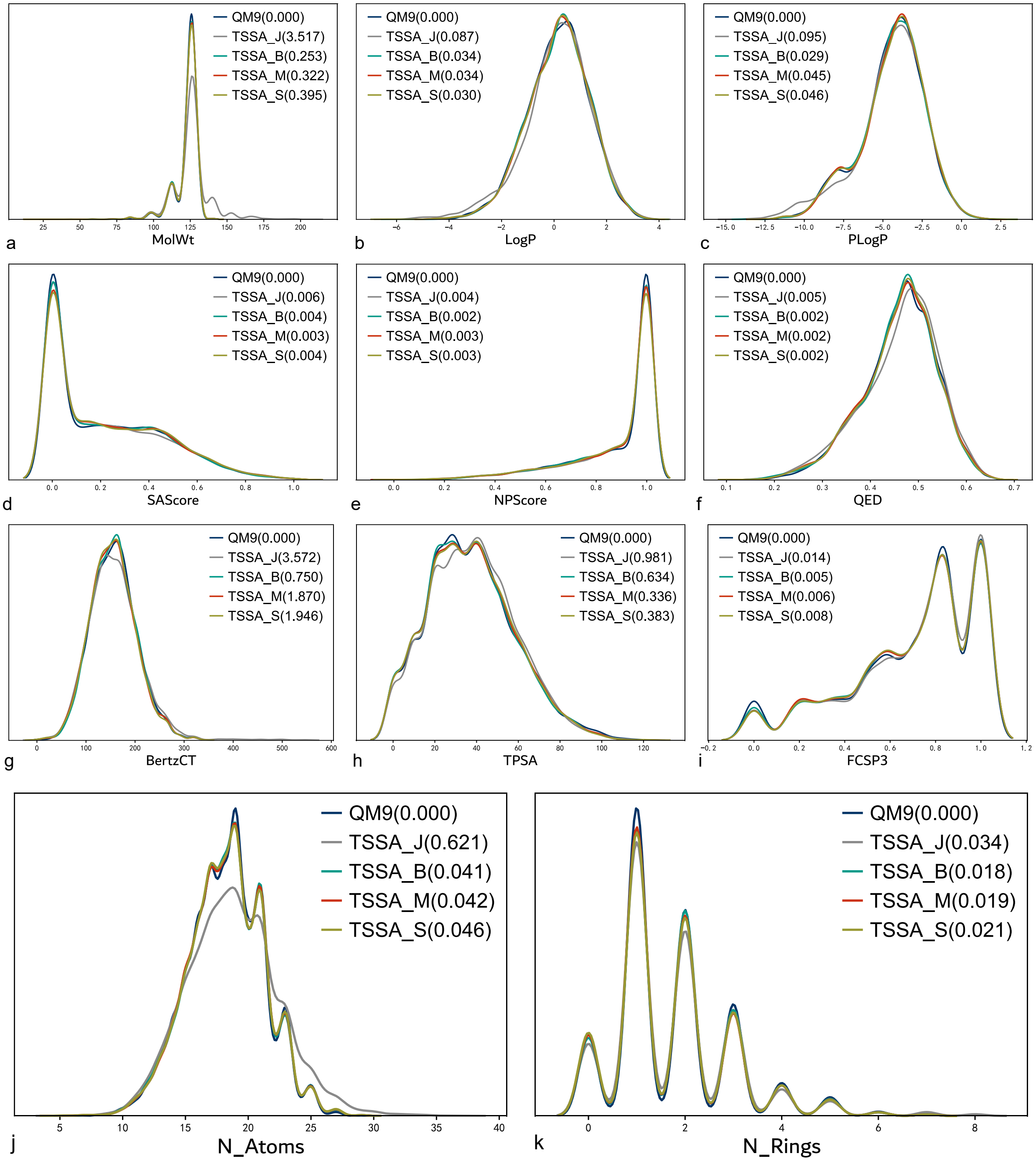

Supplementary Fig.54 Physicochemical properties of randomly reconstructed molecules on QM9. The unit of the y-axis is 'Density'.

E.4 Physicochemical Properties on ChEMBL

Supplementary Table 27 Wasserstein distance metrics for baseline description, baseline models and t-SMILES family on ChEMBL. The worst (higher) scores for each task are highlighted in bold red, scores that are better (lower) than classic SMILES are highlighted in bold blue.

Model	MolWt	LogP	pLogP	SAS	NPS	QED	BertzCT	TPSA	FCSP3
SMILES	3.657	0.045	0.075	0.007	0.016	0.007	7.613	1.220	0.006
DSMILES	12.409	0.156	0.120	0.026	0.026	0.021	40.551	**0.939**	0.007
SELFIES	4.911	**0.045**	0.302	0.033	**0.016**	**0.007**	39.716	1.229	**0.005**
hG2G	**77.934**	**0.625**	0.412	**0.118**	0.023	**0.089**	**268.146**	**16.538**	0.013
TSSA_J	5.967	0.247	0.659	0.116	0.069	0.022	51.397	**0.575**	**0.021**
TSSA_B	9.415	0.188	0.369	0.100	0.067	0.040	34.998	4.714	**0.003**
**TSSA_M**	13.748	0.238	0.440	0.059	0.056	**0.006**	29.179	2.089	0.012
TSSA_S	6.202	0.159	0.370	0.063	0.032	0.014	10.007	4.116	0.026
TSDY_B	7.048	0.083	0.121	**0.007**	**0.008**	0.019	11.361	2.301	**0.002**
TSDY_M	13.173	0.093	0.079	0.017	0.023	0.009	23.265	3.494	**0.005**
**TSDY_S**	8.321	**0.041**	0.100	0.013	**0.010**	**0.005**	17.014	1.753	0.021
TSID_B	8.433	**0.045**	0.086	**0.005**	**0.007**	**0.005**	17.421	2.387	0.008
TSID_M	16.354	0.074	0.107	0.019	**0.010**	0.021	43.373	5.102	**0.005**
**TSID_S**	5.174	**0.037**	**0.046**	**0.005**	**0.007**	0.012	11.876	**1.103**	0.009
TS_Vanilla	4.930	0.103	0.150	**0.004**	**0.006**	**0.007**	10.760	3.071	**0.005**
**TSSA_HJBMSV**	5.362	0.124	0.321	0.062 (0.063)	0.039	0.010	15.795	2.218	0.009
**TSDY_HBMSV**	7.017	0.145	0.241	0.009	**0.010**	**0.005**	11.079	1.861	0.011
**TSID_HBMSV**	7.298	0.072	0.184	0.018	0.020	**0.003**	12.029	1.277	0.009
TSDY_HBV	6.525	0.116	0.196	**0.007**	**0.008**	0.011	10.744	**0.514**	0.010
TSDY_HMV	7.186	**0.040**	0.137	0.011	**0.015**	**0.006**	9.274	2.465	0.018
TSDY_HSV	5.182	0.146	0.275	0.020	**0.012**	**0.002**	21.572	2.163	0.028
**TSID_HBV**	6.149	**0.041**	0.119	0.008	0.017	**0.006**	11.662	**1.145**	0.010

* To be noted that, molecules are randomly selected from both the training dataset and the generated group. Therefore, the results of these metrics may differ slightly each time. Small system errors are a result of random sampling. For instance, selecting a sample of 10,000 molecules out of a total of 1,570,407. A tiny system error (0.022498 vs 0.022534) was identified in the calculation of Wasserstein distances due to random sampling. So, the curve of the KDE should be the primary focus when analyzing the performance of distributed learning on properties.

Supplementary Table 28 Mean and StdDev of Wasserstein distance metrics for three samplings on ChEMBL.

	Model	MolWt	LogP	pLogP	SAS	NPS	QED	BertzCT	TPSA	FCSP3
Mean	SMILES	3.772	0.055	0.074	0.005	0.014	0.006	8.884	1.292	0.006
	DSMILES	11.180	0.147	0.110	0.025	0.022	0.020	40.742	0.938	**0.005**
	SELFIES	3.877	**0.046**	0.307	0.035	0.018	**0.006**	39.071	1.396	0.007
	hG2G	76.148	0.591	0.374	0.114	0.022	0.087	263.296	16.654	0.015
	TSID_S	3.801	**0.042**	**0.054**	**0.004**	**0.006**	0.010	**8.690**	**1.067**	**0.006**
StdDev	SMILES	0.331	0.010	0.001	0.002	0.002	0.001	1.265	0.126	0.003
	DSMILES	1.078	0.017	0.016	0.002	0.005	0.002	3.058	0.151	0.002
	SELFIES	1.925	0.001	0.014	0.004	0.002	0.001	4.560	0.170	0.002
	hG2G	2.044	0.031	0.033	0.004	0.001	0.003	7.009	0.101	0.002
	TSID_S	1.213	0.005	0.019	0.001	0.001	0.002	2.869	0.032	0.003

E.4.1 Baseline Models on ChEMBL

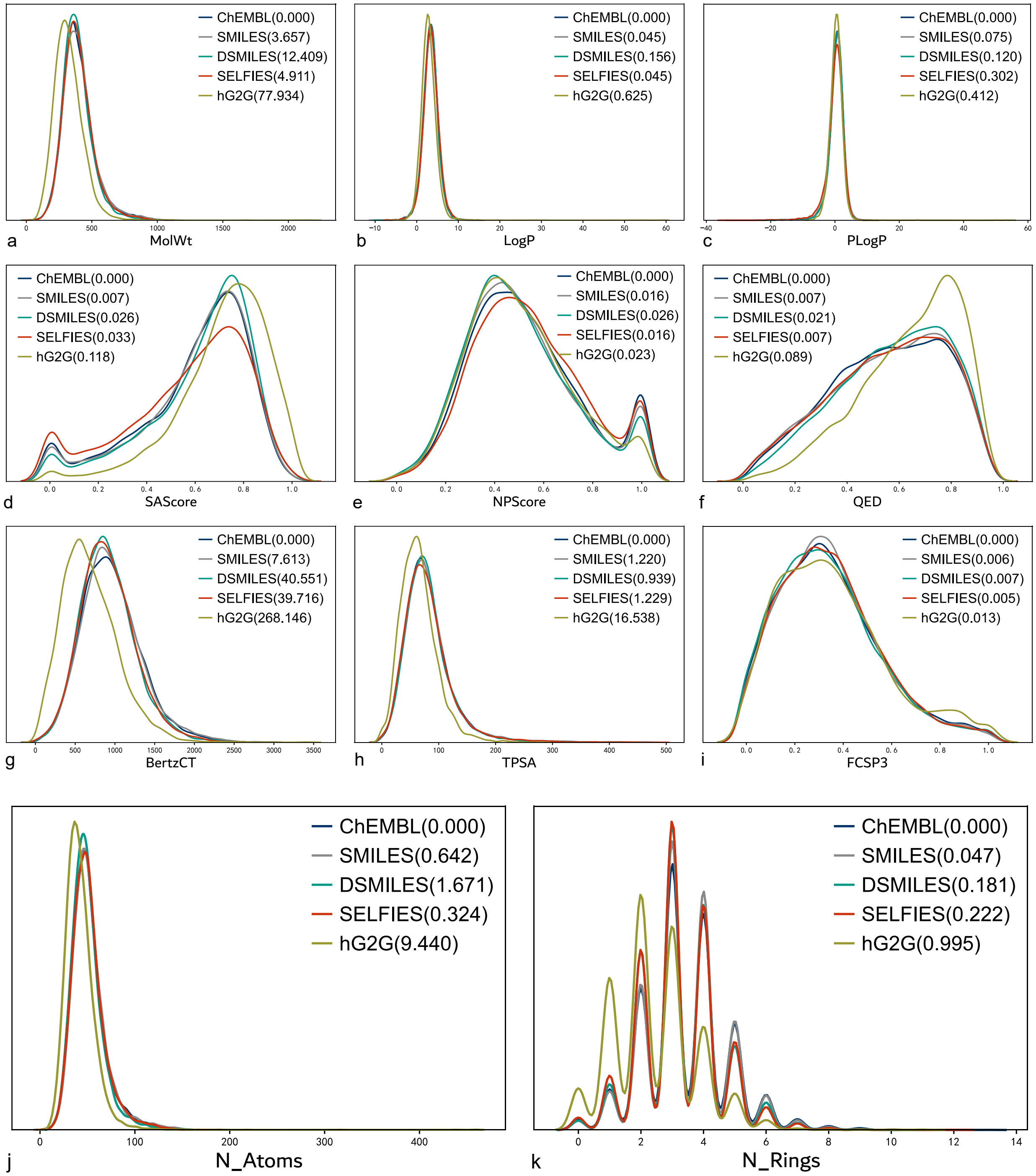

Supplementary Fig.55 Physicochemical properties of generated molecules by baseline models on ChEMBL. The unit of the y-axis is 'Density'.

E.4.2 Singleton TSSA Models on ChEMBL

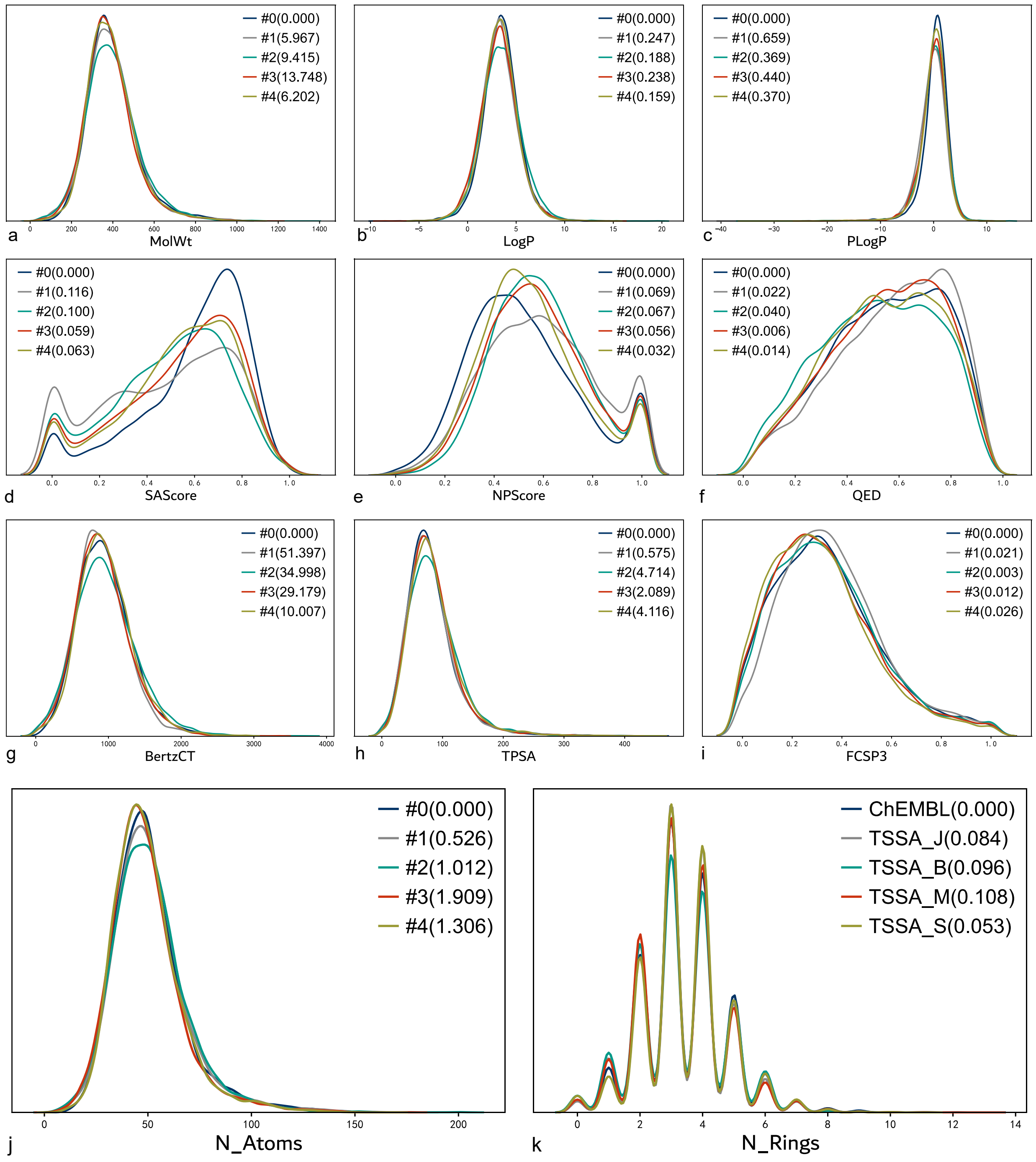

Supplementary Fig.56 Physicochemical properties of generated molecules by singleton TSSA models on ChEMBL. The unit of the y-axis is 'Density'. The figures contain labels ['#0', '#1', '#2', '#3', '#4'] corresponding to the following datasets: ['ChEMBL', 'TSSA_J', 'TSSA_B', 'TSSA_M', 'TSSA_S']

E.4.3 Singleton TSDY and TSID Models on ChEMBL

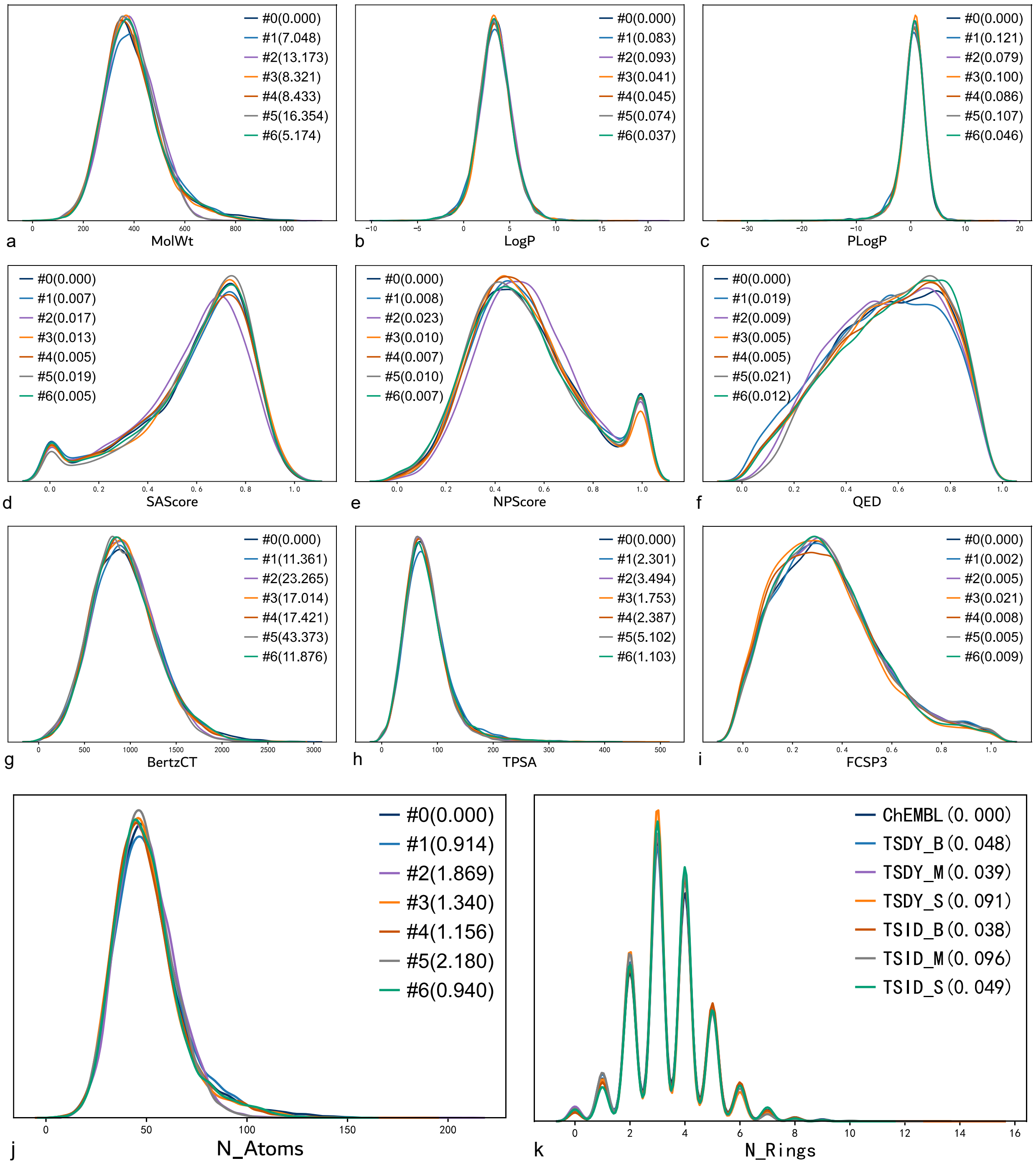

Supplementary Fig.57 Physicochemical properties of generated molecules by singleton TSDY and TSID models on ChEMBL. The unit of the y-axis is 'Density'. The figures contain labels ['#0', '#1', '#2', '#3', '#4', '#5', '#6'] corresponding to the following datasets: ['ChEMBL', 'TSDY_B', 'TSDY_M', 'TSDY_S', 'TSID_B', 'TSID_M', 'TSID_S']

E.4.4 Hybrid t-SMILES Models on ChEMBL

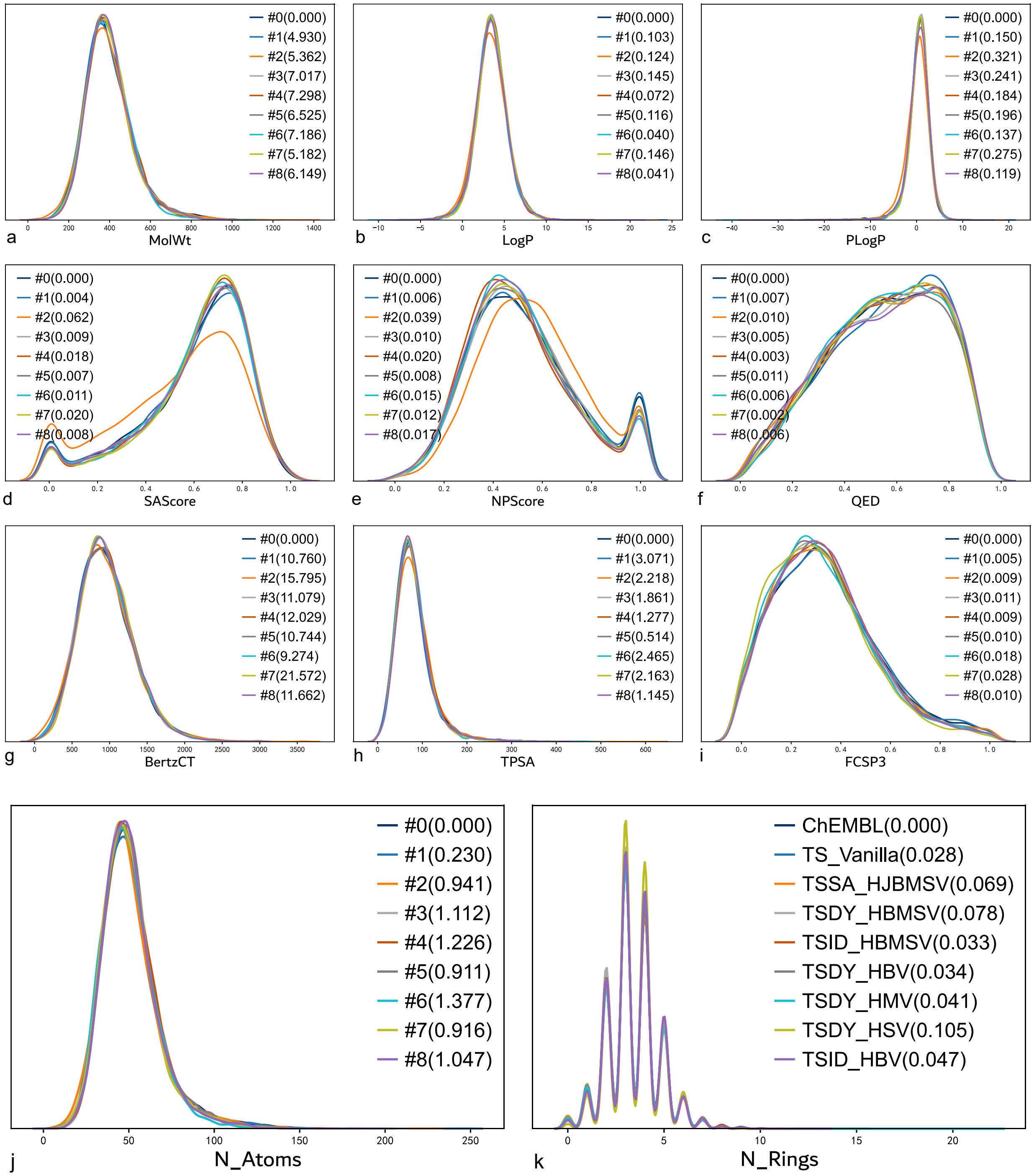

Supplementary Fig.58 Physicochemical properties of generated molecules by hybrid t-SMILES models on ChEMBL. The unit of the y-axis is 'Density'. The figures contain labels ['#0', '#1','#2','#3','#4','#5', '#6', '#7', '#8'] corresponding to the following datasets: ['ChEMBL', 'TS_Vanilla', 'TSSA_HJBMSV' (TSSA_J, TSSA_B, TSSA_M, TSSA_S and TS_Vanilla), 'TSDY_HBMSV' (TSDY_B, TSDY_M, TSDY_S and TS_Vanilla), 'TSID_HBMSV'(TSID_B, TSID_M, TSID_S and TS_Vanilla), 'TSDY_HBV', 'TSDY_HMV', 'TSDY_HSV', 'TSID_HBV'].

E.5 Physicochemical Properties on Zinc

Supplementary Table 29 Wasserstein distance metrics for baseline description, baseline models and t-SMILES family on Zinc. The worst (higher) scores for each task are highlighted in bold red, scores that are better (smaller) than classic SMILES are highlighted in bold blue.

Model	MolWt	LogP	pLogP	SAS	NPS	QED	BertzCT	TPSA	FCSP3
SMILES	4.964	0.052	0.052	0.002	0.006	0.003	7.374	1.979	0.008
DSMILES	**2.400**	**0.040**	**0.034**	**0.002**	**0.005**	0.005	7.470	**0.495**	**0.007**
SELFIES	**4.184**	**0.045**	0.109	0.019	0.011	0.010	7.772	**1.143**	**0.006**
JTVAE	46.777	**0.810**	**1.213**	0.096	**0.117**	0.024	**141.039**	2.561	0.036
FragDgm	**61.077**	0.547	0.976	**0.169**	0.091	**0.144**	147.943	**13.936**	**0.058**
BRICS_Rnd	11.168	0.359	0.738	0.118	0.108	0.019	43.565	4.371	0.038
TSSA_J	**3.358**	0.184	0.435	0.072	0.072	**0.002**	20.550	2.832	0.013
TSSA_B	**4.138**	0.117	0.300	0.081	0.086	0.044	7.961	4.458	**0.004**
TSSA_M	**2.789**	0.227	0.481	0.072	0.081	0.025	**6.620**	5.006	**0.002**
TSSA_S	**2.668**	0.213	0.532	0.088	0.076	0.035	**5.460**	6.678	**0.005**
TSDY_B	**3.812**	**0.033**	0.067	0.017	0.017	0.013	**5.462**	**0.582**	0.012
TSDY_M	5.943	0.104	0.063	0.021	0.032	0.011	19.133	**1.274**	0.011
TSDY_S	**2.553**	0.060	**0.051**	0.010	0.011	0.010	10.280	**0.878**	**0.007**
TSID_B	6.385	**0.052**	**0.047**	0.013	0.014	0.018	18.087	**0.929**	**0.006**
TS_Vanilla	**4.952**	**0.040**	**0.046**	0.003	0.007	0.004	11.825	1.981	**0.006**
TSSA_HJBMSV	**3.210**	0.140	0.361	0.058	0.066	0.022	9.162	3.284	**0.003**
TSDY_HBMSV	7.365	**0.044**	0.082	0.022	0.019	0.016	18.954	**1.507**	**0.003**
TSDY_HBV	**4.468**	0.092	0.094	0.004	**0.005**	0.010	9.354	**0.684**	**0.004**
TSDY_HMV	**3.494**	**0.047**	**0.032**	0.010	0.011	0.004	8.732	**0.837**	**0.006**
TSDY_HSV	**2.541**	**0.034**	0.057	0.008	0.008	0.007	7.995	**1.221**	**0.006**
TSID_HBV	5.565	**0.035**	0.062	0.013	**0.007**	0.008	11.588	**0.790**	**0.005**

* To be noted that, molecules are randomly selected from both the training dataset and the generated group. Therefore, the results of these metrics may differ slightly each time. Any small system errors are a result of random sampling. For instance, selecting a sample of 10,000 molecules out of a total of 249,454. A tiny system error was identified in the calculation of Wasserstein distances due to random sampling. So, the curve of the KDE should be the primary focus when analyzing the performance of distributed learning on properties.

E.5.1 Baseline Models on Zinc 1

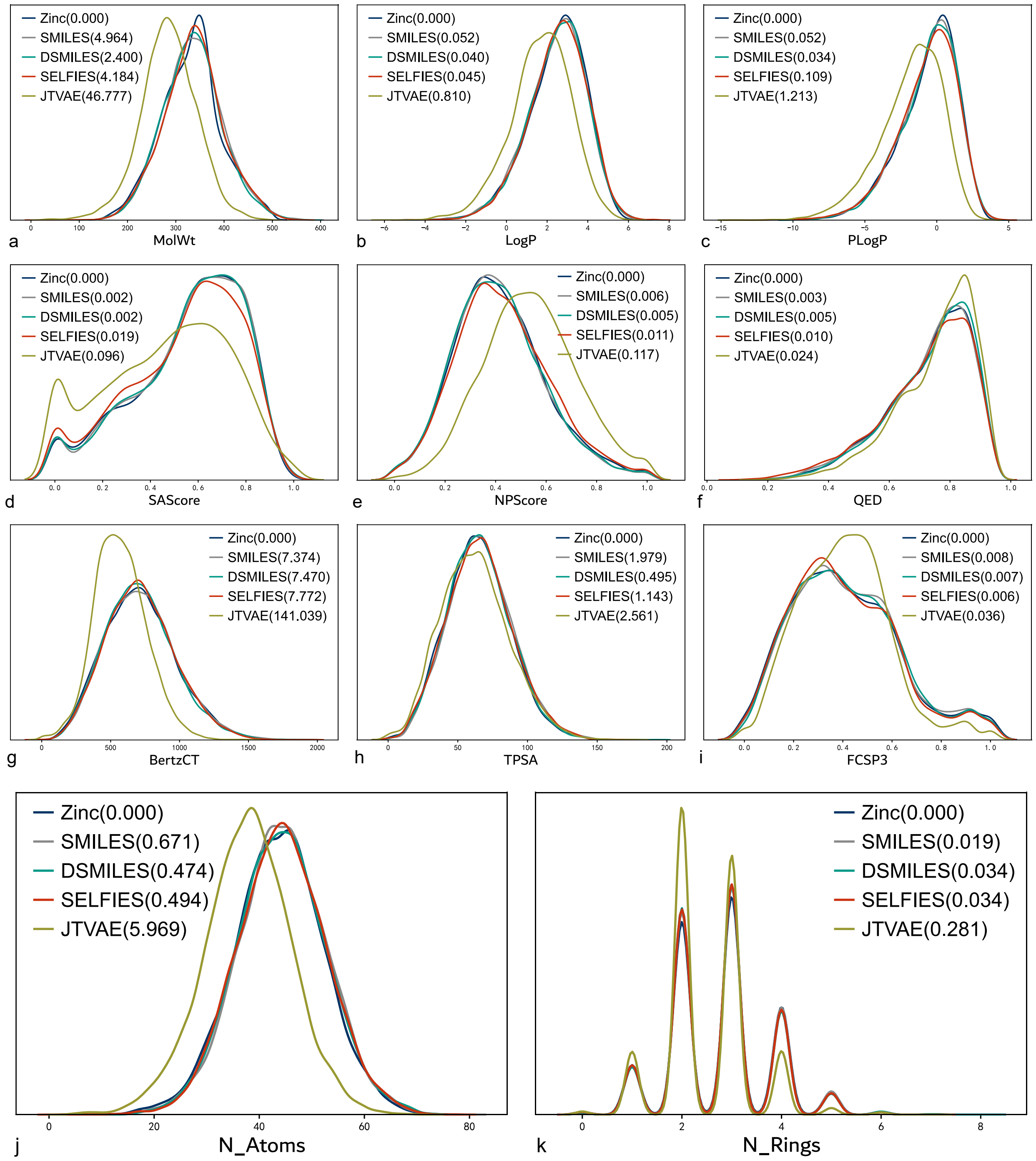

Supplementary Fig.59 Physicochemical properties of generated molecules by baseline models on Zinc. The unit of the y-axis is 'Density'.

E.5.2 Baseline Models on Zinc 2

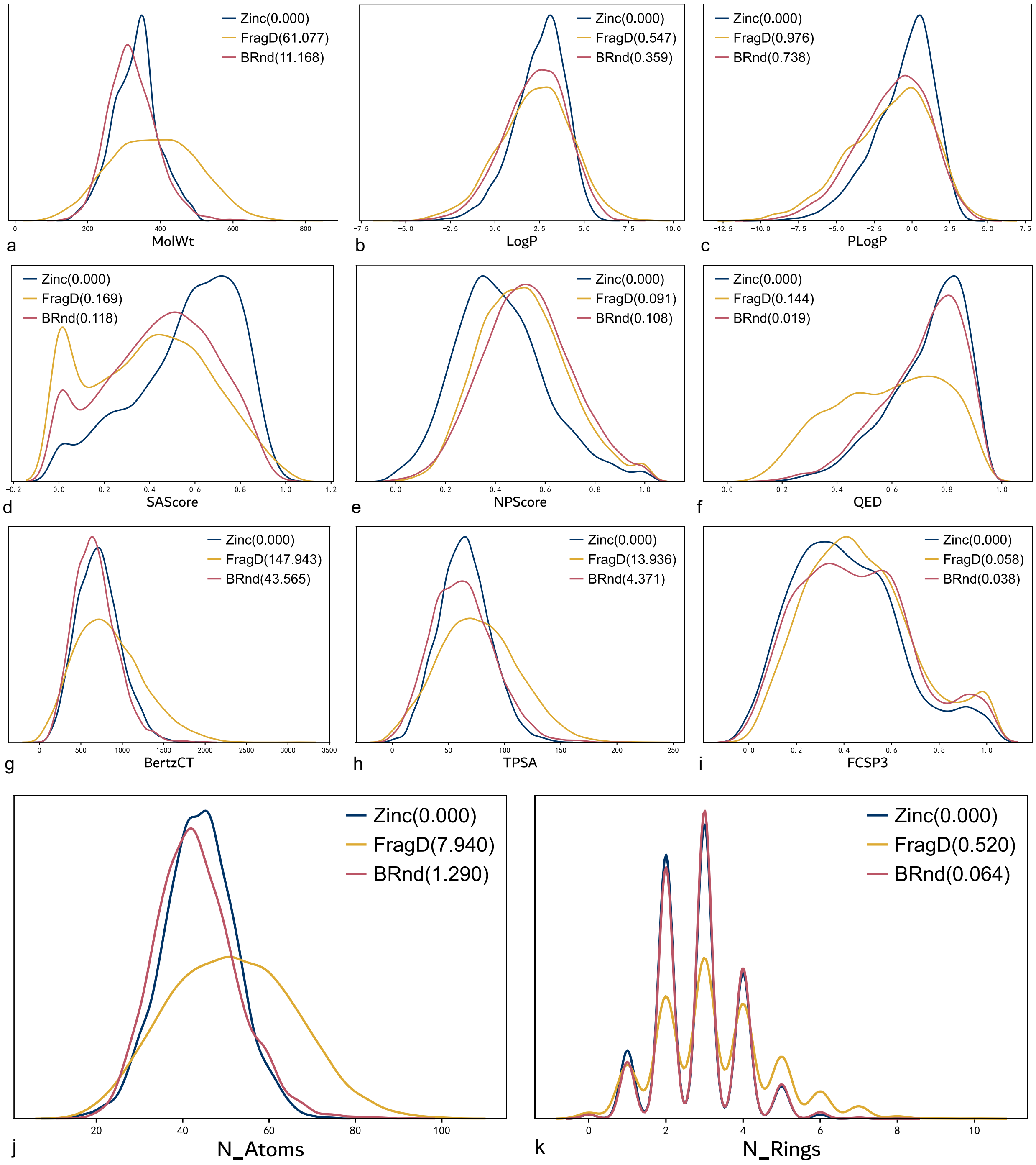

Supplementary Fig.60 Physicochemical properties of generated molecules by baseline models: FragDgm and BRICS_Rnd on Zinc. The unit of the y-axis is 'Density'. Label ‘FragD’ means model: Fragment-base-DGM(FragDgm)[27], ‘BRnd’ means model: BRICS_Random. FragDgm splits molecule in a linear mode as a sequence of fragment IDs. Although FragDgm uses a segmented mode and being based on distributional learning, its FCD value of 0.303 is the lowest among all listed models.

E.5.3 Singleton t-SMILES Models on Zinc

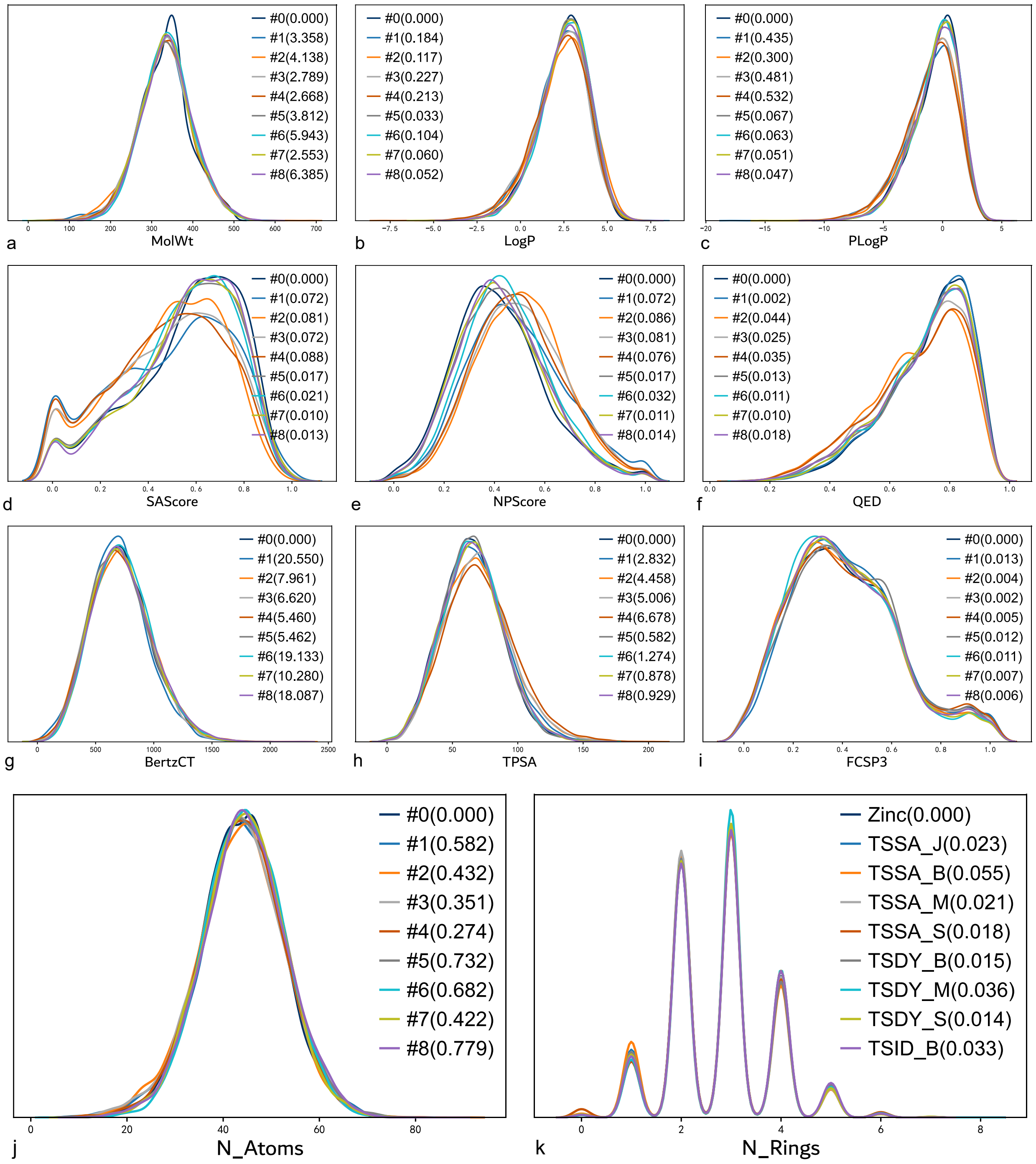

Supplementary Fig.61 Physicochemical properties of generated molecules by singleton t-SMILES models on Zinc. The unit of the y-axis is 'Density'. The figures contain labels ['#0', '#1','#2','#3','#4','#5', '#6', '#7', '#8'] corresponding to the following datasets: ['Zinc', 'TSSA_J', 'TSSA_B','TSSA_M', 'TSSA_S', 'TSDY_B','TSDY_M', 'TSDY_S', 'TSID_B']

E.5.4 Hybrid t-SMILES Models on Zinc

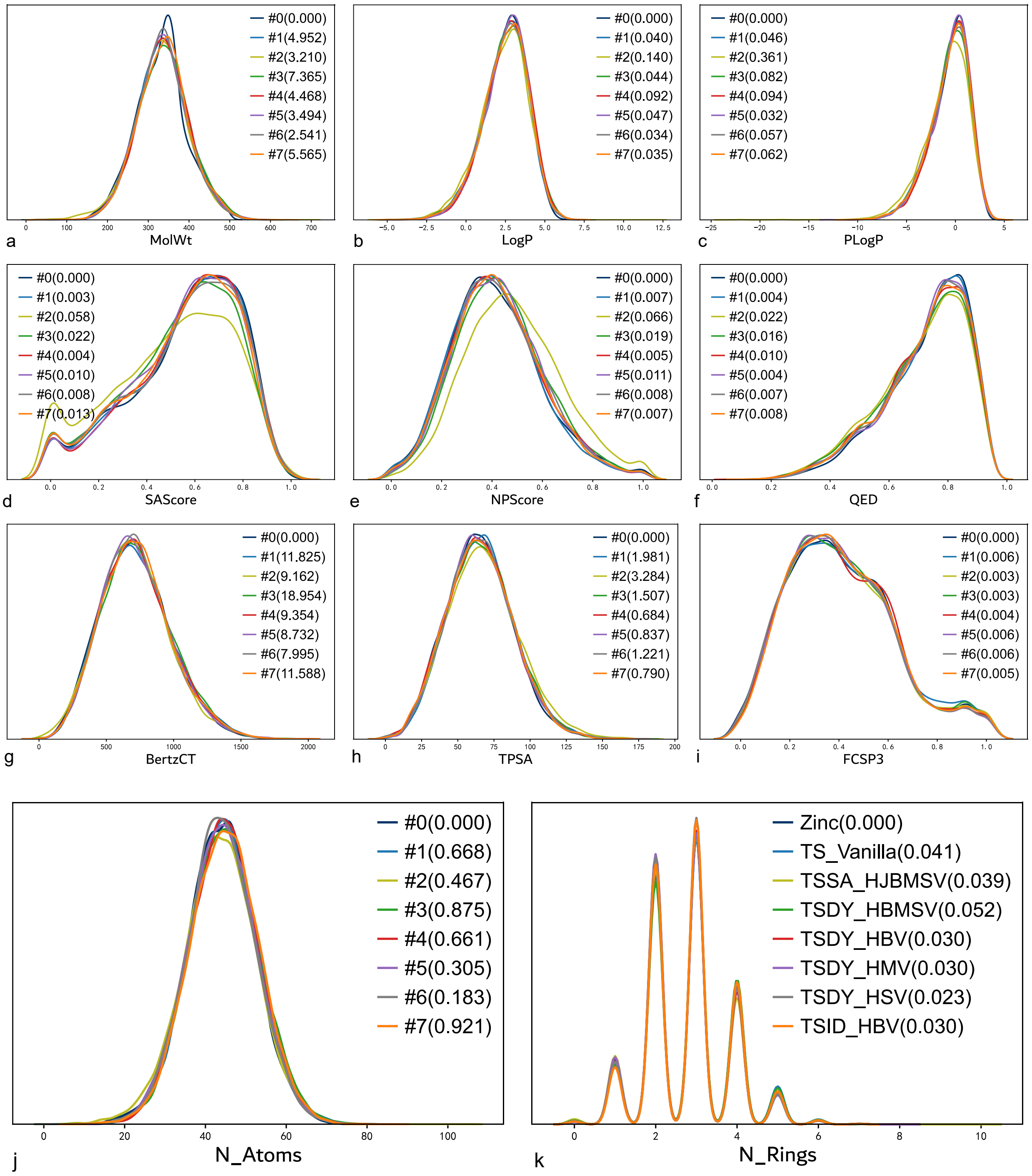

Supplementary Fig.62 Physicochemical properties of generated molecules by hybrid t-SMILES models on Zinc. The unit of the y-axis is 'Density'. The figures contain labels ['#0', '#1','#2','#3','#4','#5', '#6', '#7'] corresponding to the following datasets: ['Zinc', 'TS_Vanilla', 'TSSA_HJBMSV'(TS_Vanilla, TSSA_J, TSSA_B, TSSA_M, TSSA_S), 'TSDY_HBMSV' (TS_Vanilla, TSDY_B, TSDY_M, TSDY_S), 'TSDY_HBV','TSDY_HMV', 'TSDY_HSV', 'TSID_HBV']

E.6 Experiments on QM9

E.6.1 Distribution Learning on QM9

As shown in Supplementary Table 29, all of the string-based QM9 models, except CharacterVAE, achieve FCD scores close to or greater than 0.96, while having reasonably lower novelty scores. This demonstrates their ability to accurately capture the features present in the training data. From the perspective of sequence-based models, our approach performs better than existing string-based approaches. Compared to graph-based baseline models, our approach demonstrates superior performance. Among the three alternative string representations, SELFIES model achieves higher novelty and FCD scores than SMILES and DSMILES. Please refer to SI.E.6.2 for physicochemical property metrics.

The TSSA_J[R10] model get the highest novelty score of 0.383 when training 10 rounds. In comparison, we train TSID_M, TSID_S and SMILES[R2] for only 2 epochs on purpose to achieve higher novelty scores and reasonable lower FCD scores. Therefore, there are five models get higher novelty score than 0.250, which are TSSA_J, TSDY_S, TSID_M, TSID_S and SMILES[R2]. In this scenario, t-SMILES models outperform SMILES as well. Please refer to SI.E.6.3 for physicochemical property metrics.

Supplementary Table 30 Distributional results on QM9 using GPT. The results of CharacterVAE[52,31], Transformer Reg[31], GraphVAE[53,31], MolGAN[30,31] and MGM[31] are taken from O. Mahmood *et al*.[31].

	Model	Valid	Unique	Novelty	KLD	FCD	Nov./Uni.
Baseline SMILES	CharacterVAE[52,31]	0.103	0.675	0.900	N/A	N/A	N/A
	Transformer Reg[31]	0.965	0.957	0.183	0.994	0.958	N/A
Baseline Graph	GraphVAE[53,31]	0.557	0.760	0.616	N/A	N/A	N/A
	MolGAN[30,31]	0.981	0.104	0.942	N/A	N/A	N/A
	MGM[31]	0.886	0.978	0.518	0.966	0.842	N/A
String	SMILES[R10]	0.984	0.951	0.138	0.998	0.983	0.145
	SMILES[R2]	0.897	0.871	**0.299**	0.992	0.973	0.343
	DSMILES[R10]	0.966	0.932	0.155	0.998	0.981	0.166
	SELFIES[R10]	1.000	0.963	0.170	0.997	0.985	0.177
t-SMILES Family	TS_Vanilla[R10]	1.000	0.965	0.187	0.995	0.978	0.193
	TSSA_J[R10]	1.000	0.928	**0.383**	0.971	0.970	0.412
	TSSA_B[R10]	1.000	0.946	0.173	0.997	0.977	0.183
	TSSA_M[R10]	1.000	0.906	0.227	0.995	0.974	0.250
	TSSA_S[R10]	1.000	0.897	0.241	0.994	0.970	0.269
	TSDY_B[R10]	1.000	0.966	0.155	0.998	0.981	0.161
	TSDY_M[R10]	1.000	0.964	0.239	0.997	0.979	0.248
	TSDY_S[R10]	1.000	0.966	**0.252**	0.997	0.981	0.261
	TSID_B[R10]	1.000	0.964	0.163	0.997	0.982	0.169
	TSID_M[R2]	0.998	0.970	**0.406**	0.989	0.967	0.419
	TSID_S[R2]	0.999	0.968	**0.363**	0.992	0.970	0.375

E.6.2 Physicochemical Properties on QM9

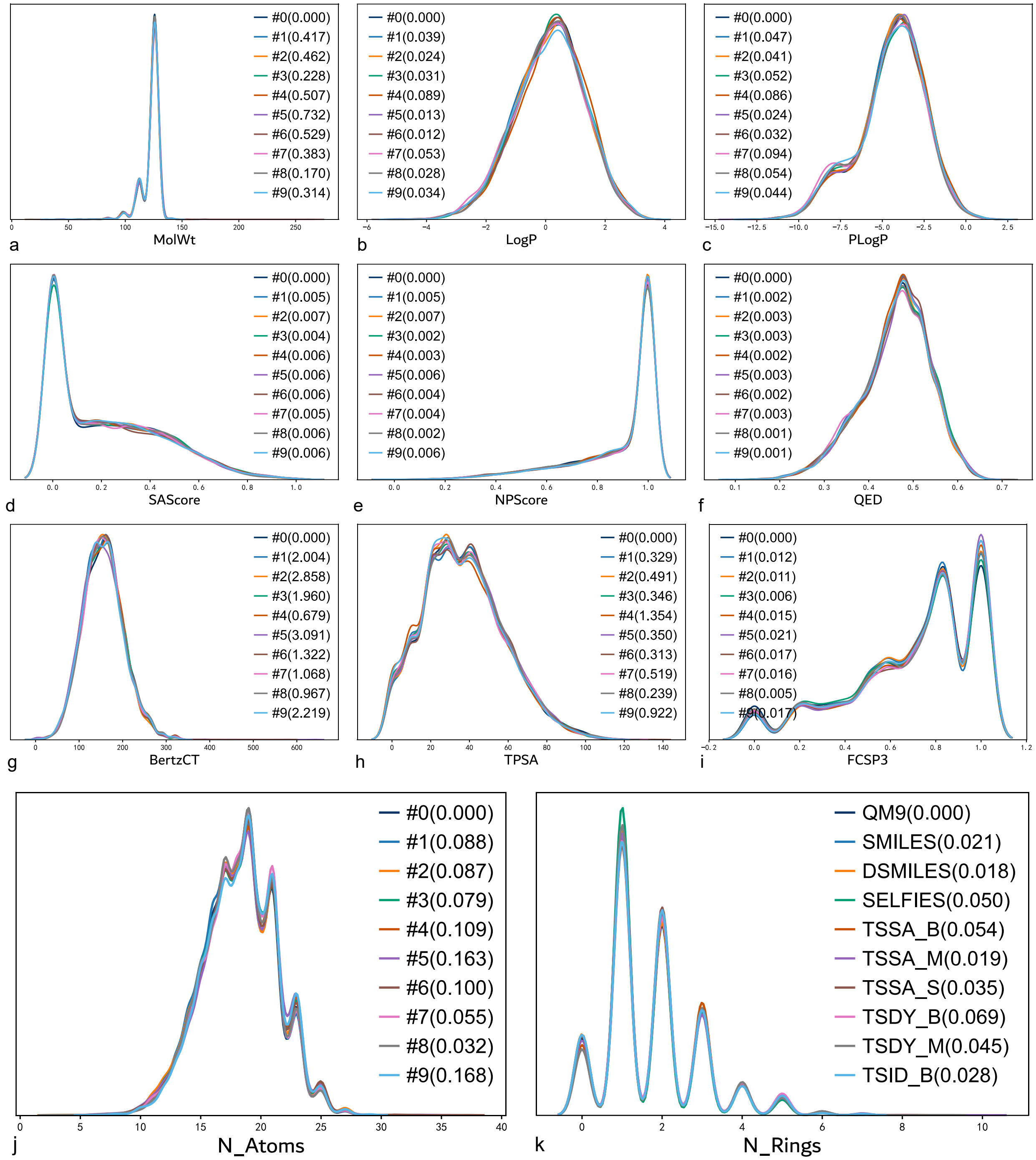

Supplementary Fig.63 Physicochemical properties of generated molecules on QM9. The unit of the y-axis is 'Density'. The figures contain labels ['#0','#1','#2','#3','#4','#5','#6','#7','#8','#9'] corresponding to the following datasets: ['QM9','SMILES', 'DSMILES', 'SELFIES', 'TSSA_B', 'TSSA_M', 'TSSA_S', 'TSDY_B', 'TSDY_M', 'TSID_B']

E.6.3 Models with Higher Novelty Scores on QM9

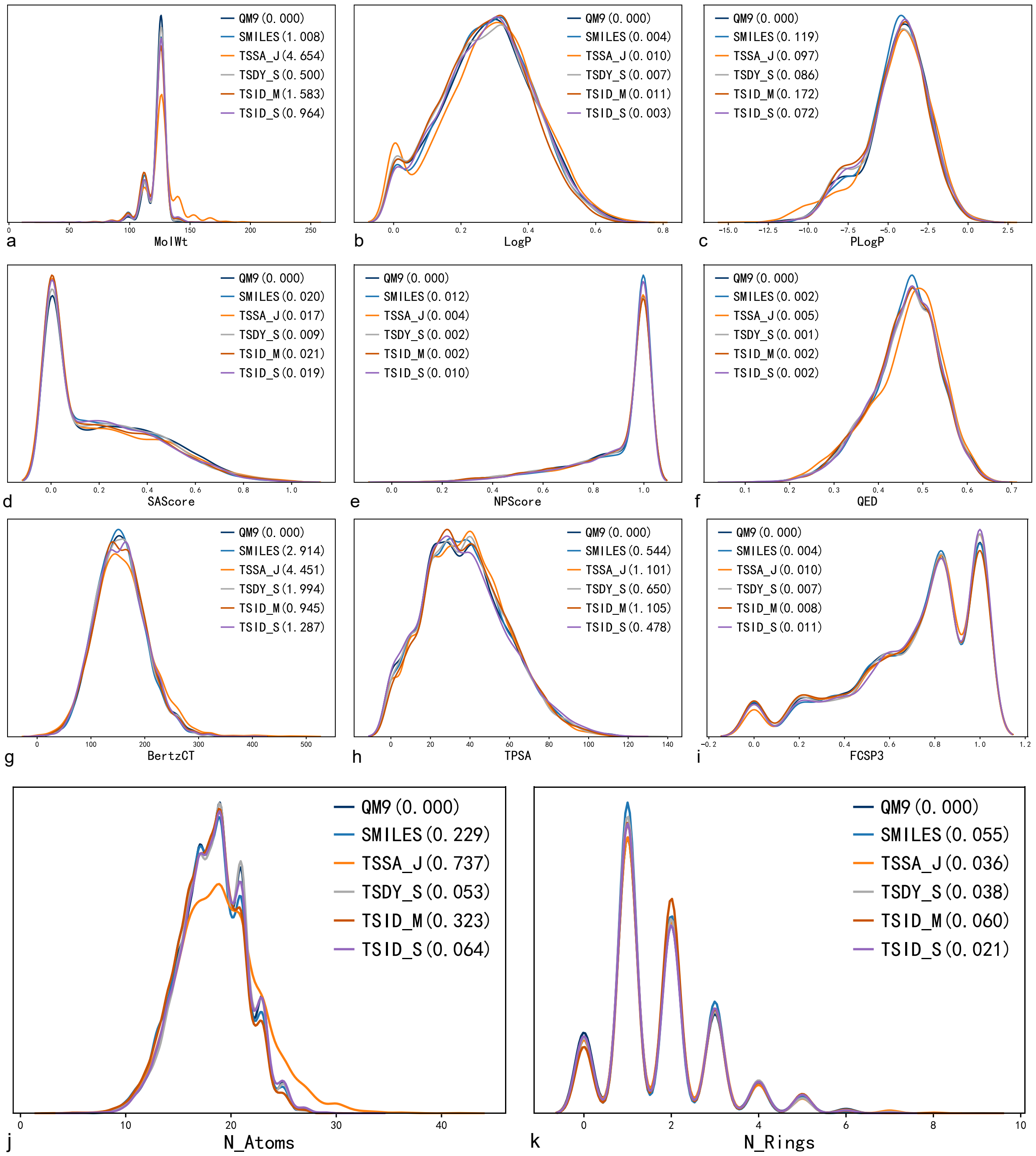

Supplementary Fig.64 Physicochemical properties of generated molecules with High Novelty Scores on QM9. The unit of the y-axis is 'Density'.

Figures indicate that the TSSA_J generates more molecules with greater molecular weights which is likely because these molecules contain more atoms. But other models fit the training data well.

E.7 Experiments using LSTM and 5 Layers miniGPT

Supplementary Table 31 Results of experiments using LSTM and 5 Layers miniGPT.

Dataset	Model	Valid(↑)	Uniq(↑)	Novel(↑)	KLD(↑)	FCD(↑)
ChEMBL	SMILES_LSTM	0.774	0.609	0.598	0.943	0.725
	SMILES_miniGPT	0.850	0.670	0.641	0.972	0.809
	t-SMILES_J_LSTM	1.000	**0.782**	0.781	0.944	0.670
	t-SMILES_J_miniGPT	1.000	0.781	0.765	0.913	0.564
Zinc	SMILES_LSTM	0.850	0.667	0.666	0.966	0.831
	SMILES_miniGPT	0.853	0.674	0.672	0.960	0.830
	t-SMILES_J_LSTM	1.000	0.782	0.781	0.944	0.670
	t-SMILES_J_miniGPT	1.000	0.775	0.774	0.970	0.773
QM9	SMILES_LSTM	0.899	0.690	0.182	0.992	0.965
	SMILES_miniGPT	0.949	0.728	0.172	0.992	0.970
	t-SMILES_J_LSTM	1.000	0.736	0.325	0.966	0.950
	t-SMILES_J_miniGPT	1.000	0.720	0.289	0.976	0.953

E.8 Experiments for Open-Ring on ChEMBL

Breaking down molecules into pieces is a challenging problem but isn't the main concept of this research. The four published fragmentation algorithms used in this study (BRICE, MMPA, Scaffold, and JTVAE) do not break rings. To assess the scalability and adaptability of t-SMILES for open-ring problem , we take RBrics[54] to fragment molecules and use TSID as target t-SMILES code algorithm. The experiments on CheEMBL are described in detail below.

Table and Figure show that there are indeed some differences on N-Rings metric between open-ring and normal BRICS algorithm. But overall, TSID based models can learn well from training data and get expected results.

E.8.1 Distribution Learning and Physicochemical Properties

Supplementary Table 32 Results for the Distribution-Learning Benchmarks on ChEMB to support Open-Ring using GPT.

Model	Valid	Unique	Novelty	KLD	FCD	Nov./Uni.
TSID_B_[R10]	1.000	0.999	0.937	0.988	0.889	0.938
TSID_RB_[R10]	1.000	0.998	0.930	0.982	0.857	0.932

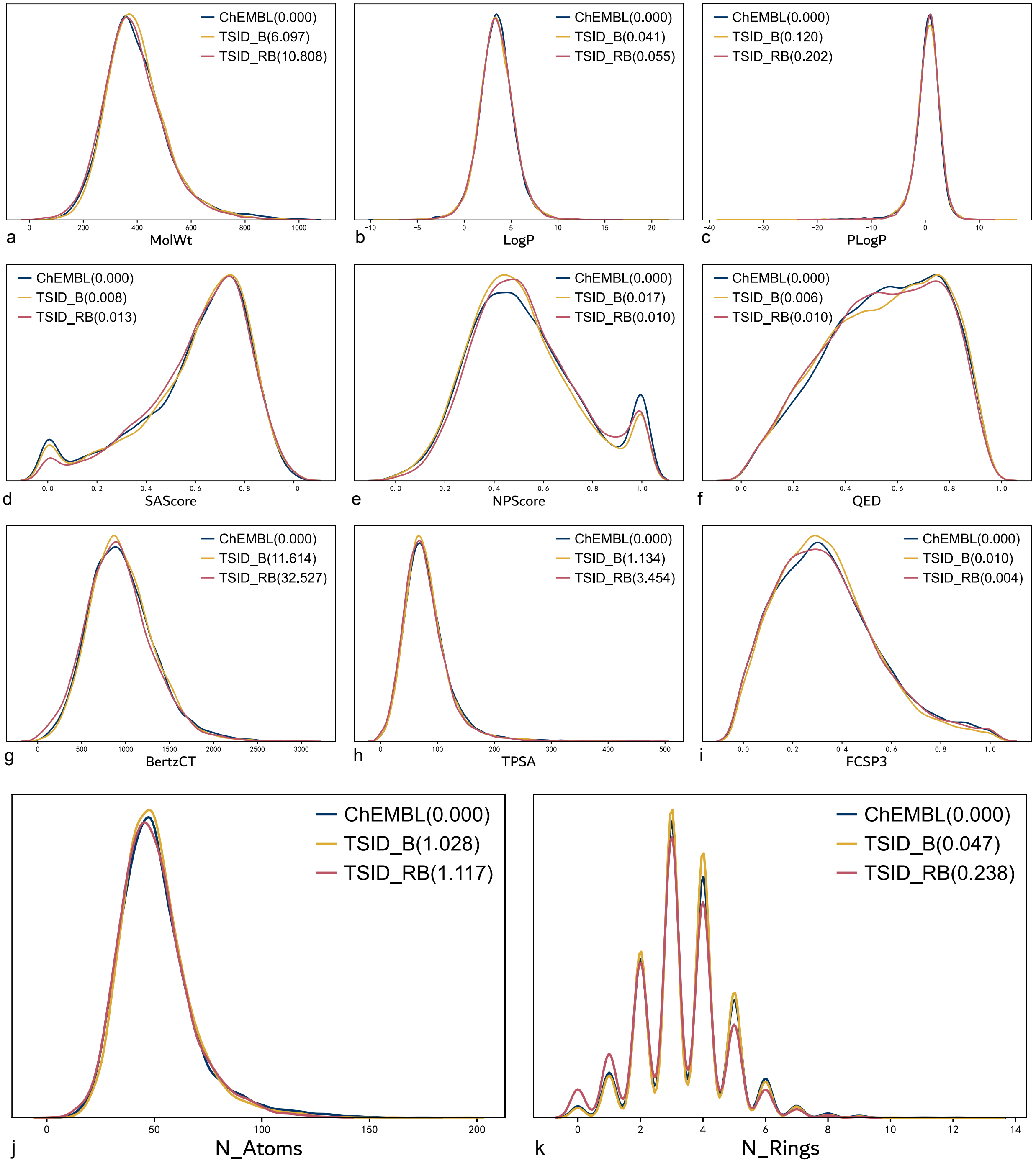

Supplementary Fig.65 Physicochemical properties of generated molecules by TSID_RB model on ChEMBL. The unit of the y-axis is 'Density'.

E.8.2 Supplementary Discussion

RBrics could break rings in some molecules like below one:

SMILES:= 'FC(F)(F)C1=NS(=O)(=O)C2=C(N1)C=C(C=C2)C1CCCC1'

TSID_RBRICS:= '[1*]C(F)(F)F&[1*]C(=NS([3*])(=O)=O)N[4*]&[2*]c1ccc([3*])c([4*])c1&[2*]C1CCCC1&&&'

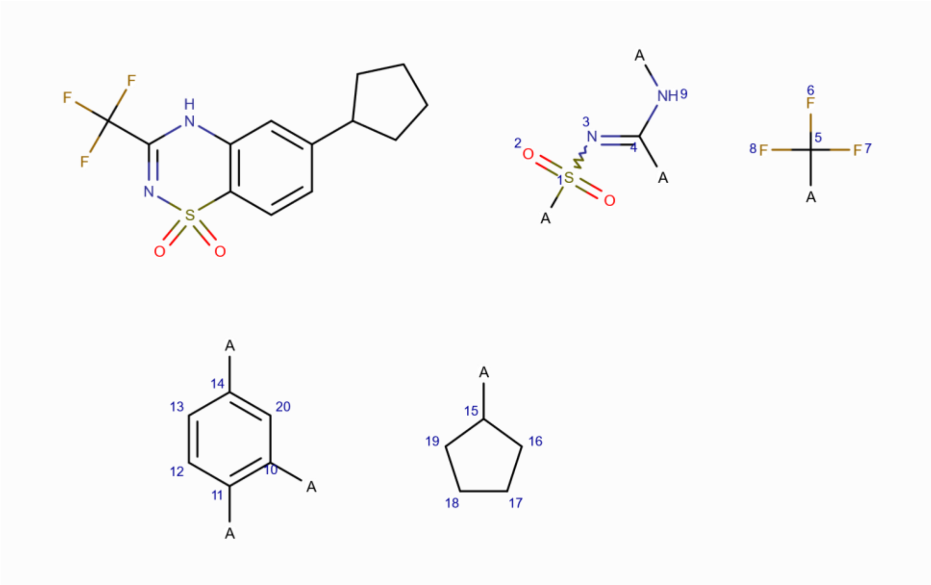

Supplementary Fig.66 SI.Fig.E.8.2.1 The first molecule is the original one, which is broken down to pieces.

However, it breaks some molecules like **CNC1CC2CCC1C2** down to too many pieces.

Although t-SMILES could correctly encode it as TSID style string:

[1*]NC&[1*]C([7*])C[2*]&[2*]C([3*])[4*]&[3*]CC[5*]^[5*]C([6*])[7*]&[4*]C[6*]&&&&

It is worth discussing whether this level of detailed decomposition meets the expectations of fragmentation methods, both from a chemical and an algorithmic perspective. We are expecting that t-SMILES could integrate more and more published fragmentation algorithms to help chemists solve practical problems.

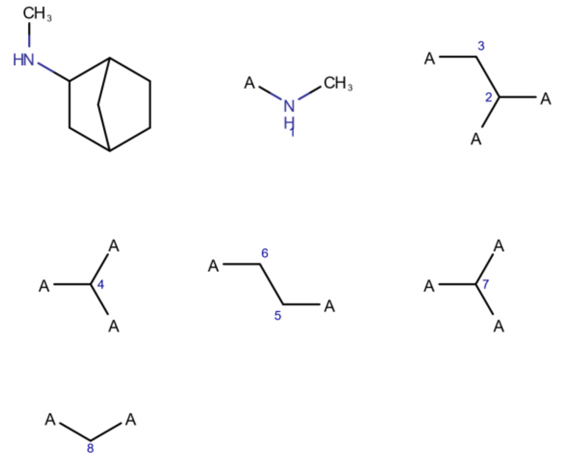

Supplementary Fig.67 SI.Fig.E.8.2.2 The first molecule is the original one, which is broken down to pieces

F. Newly Generated Molecules

Molecules are selected randomly from the training dataset and the generated sets. The generated molecules are checked by RDKit only after they can be parsed correctly. In real-world experiments, it is recommended to use more conditions to filter molecules and obtain the target molecules.

F.1.0 Molecules on Training Dataset ChEMBL (Randomly Selected)

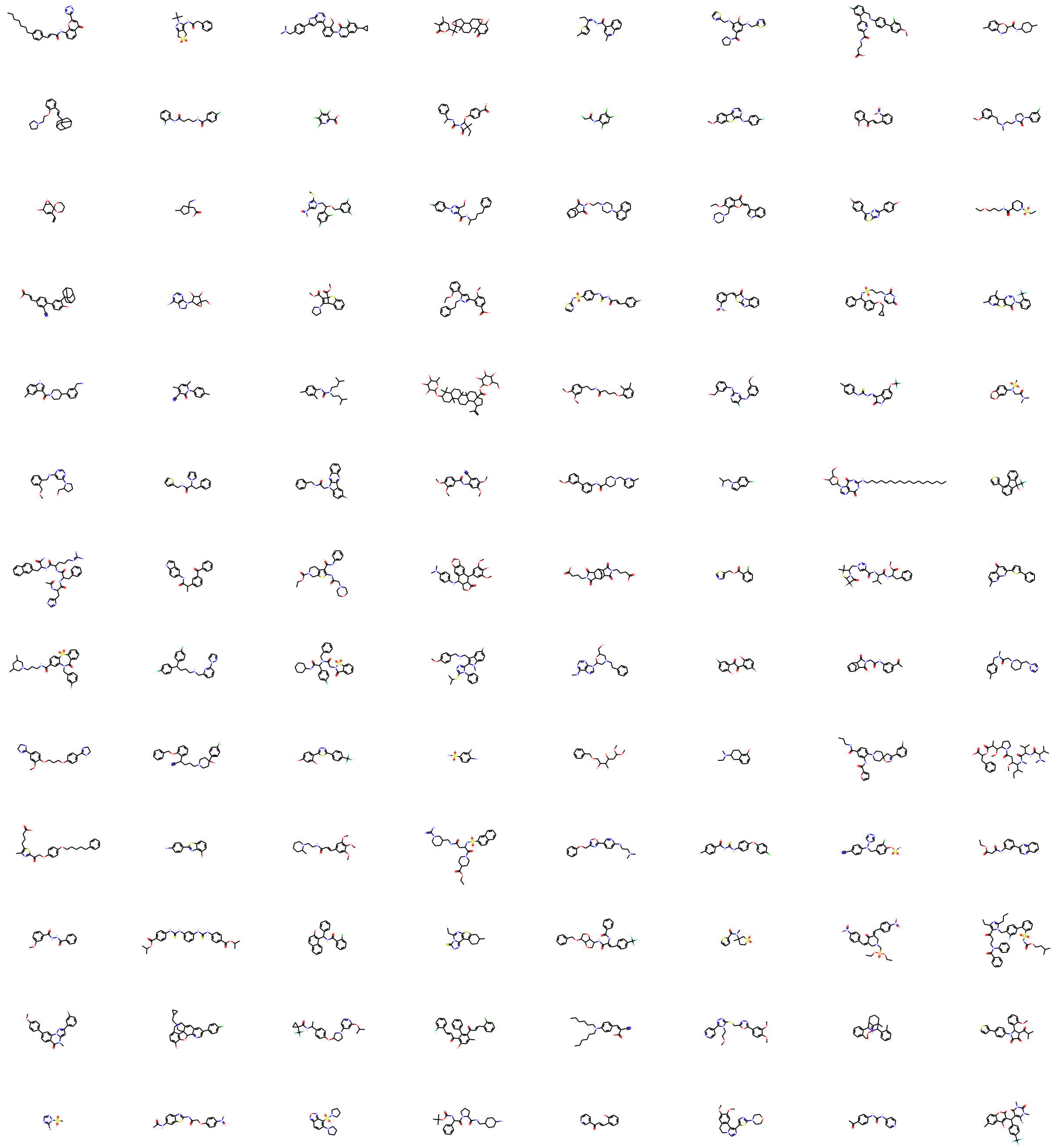

Supplementary Fig.68 F.2.0 Molecules on Training Dataset ChEMBL (Randomly Selected)

F.1.1 Generated Molecules on ChEMBL (Randomly Selected, TSDY_HBMSV)

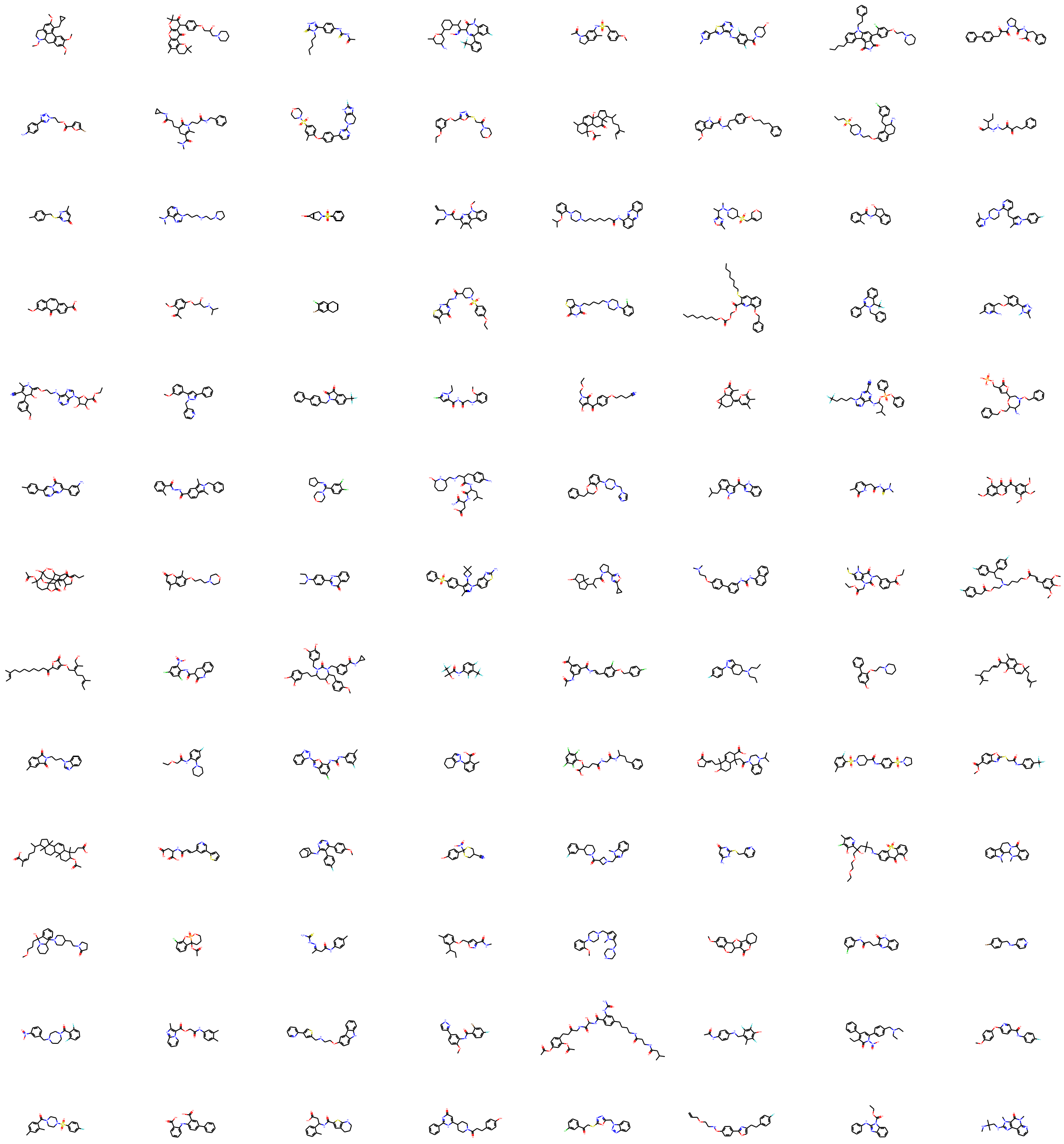

Supplementary Fig.69 Generated Molecules on ChEMBL (Randomly Selected, TSDY_HBMSV)

F.2.0 Molecules on Training Dataset Zinc (Randomly Selected)

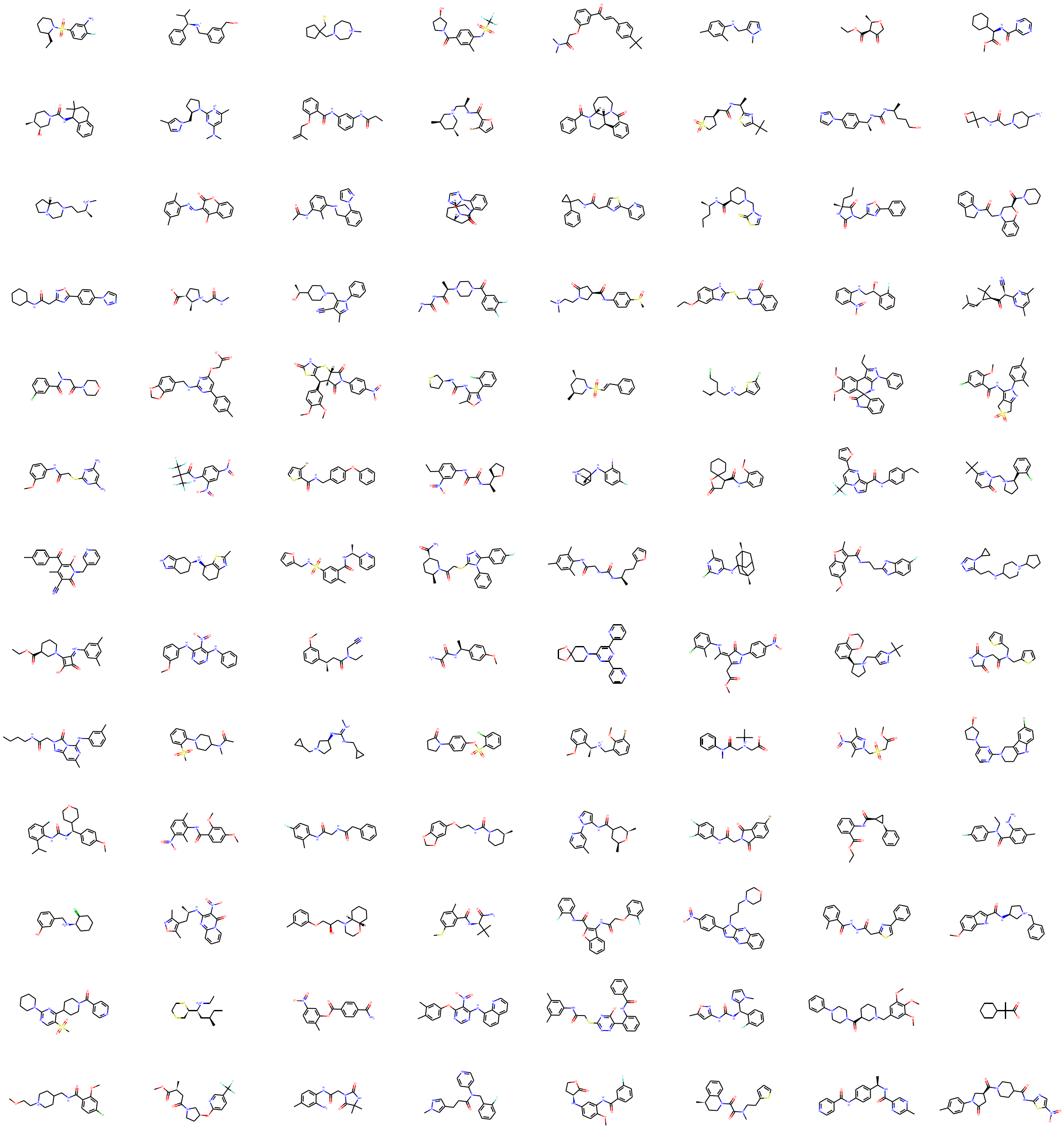

Supplementary Fig.70 Molecules on Training Dataset Zinc (Randomly Selected)

F.2.1 Generated Molecules on Zinc (Randomly Selected)

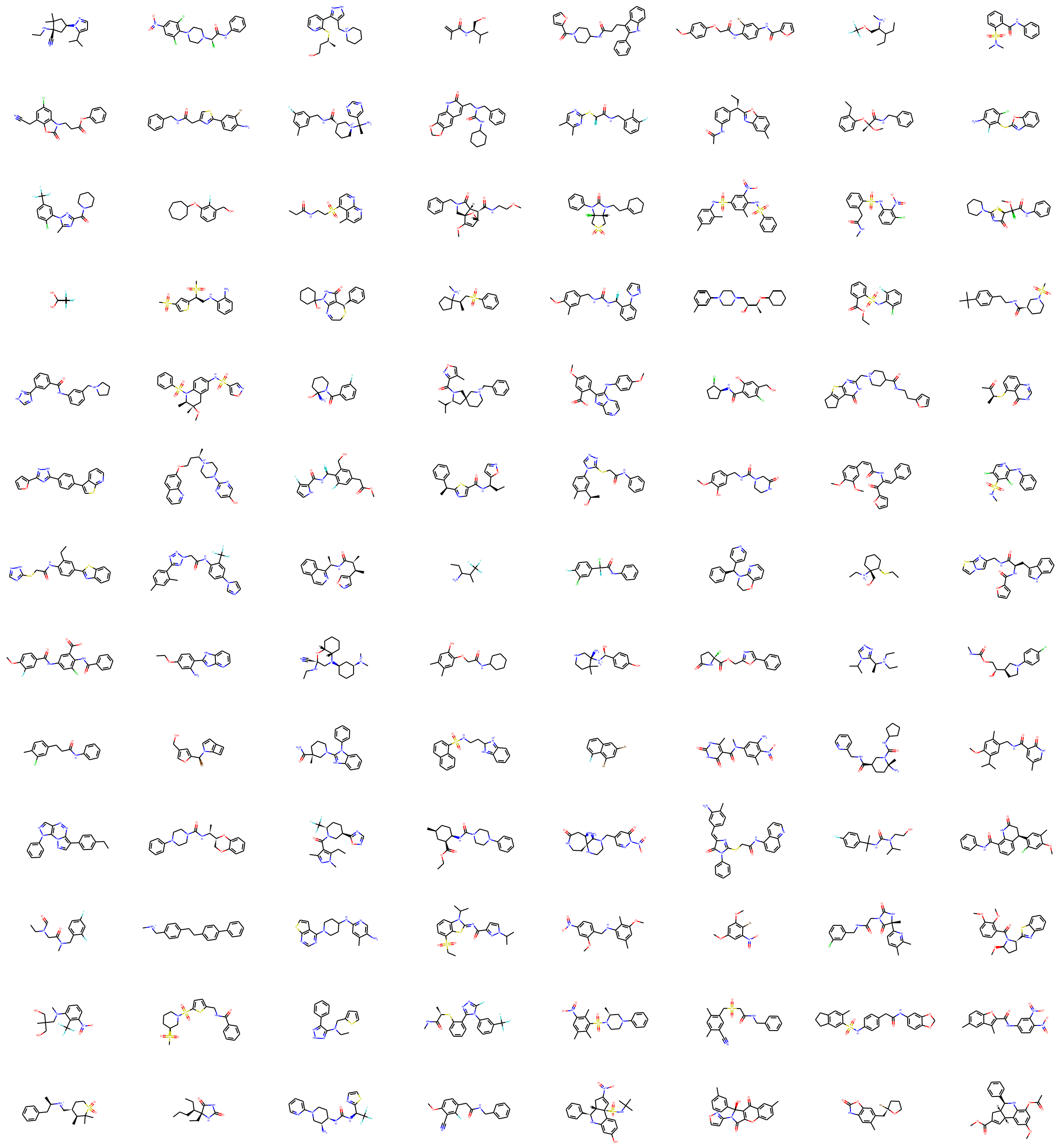

Supplementary Fig.71 Generated Molecules on Zinc (Randomly Selected)

F.3.0 Molecules on Training Dataset QM9 (Randomly Selected)

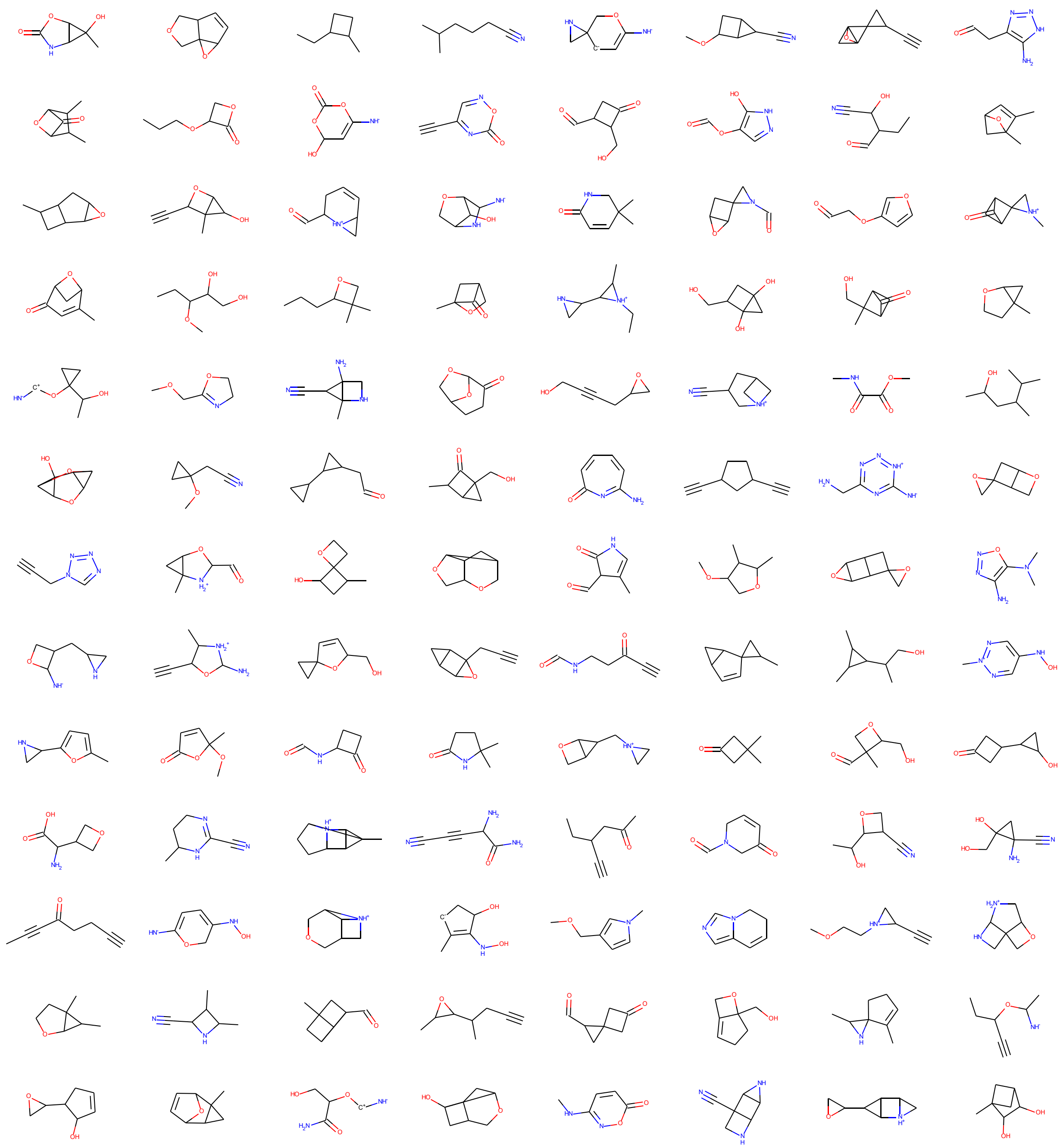

Supplementary Fig.72 Molecules on Training Dataset QM9 (Randomly Selected)

F.3.1 Generated Molecules on QM9 (Randomly Selected)

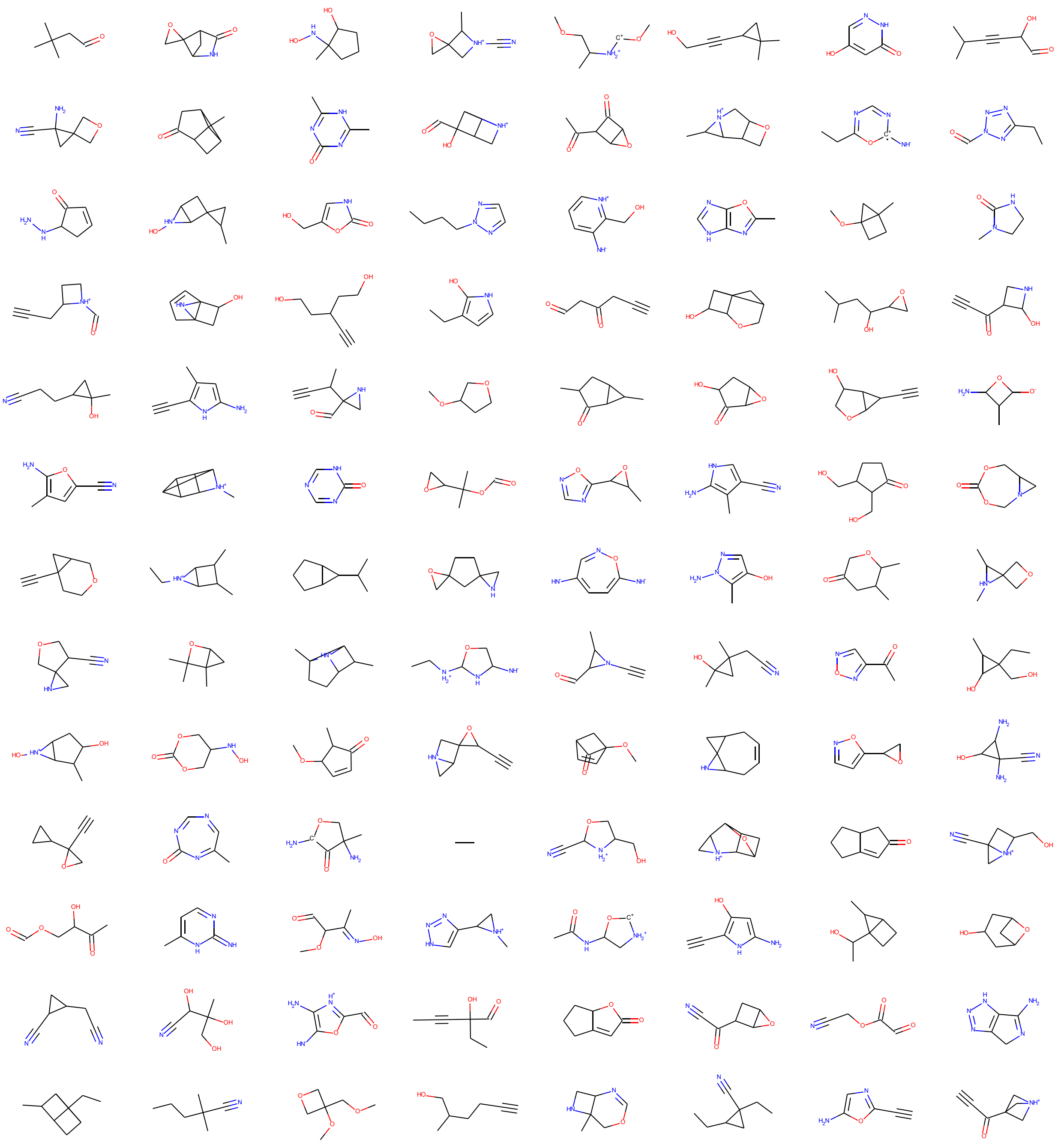

Supplementary Fig.73 Generated Molecules on QM9 (Randomly Selected)

G. Atom Environment Substructure on ChEMBL

Atom environments is defined in RDKit as atoms plus the neighbor atoms with chemical bonds. Top 20 atom environment (radius= 3, based on dummy atom '*') in ChEMBL after decomposed and generated. We use this as a weighting function to rank the assembly of fragments.

TSDY_B Dataset

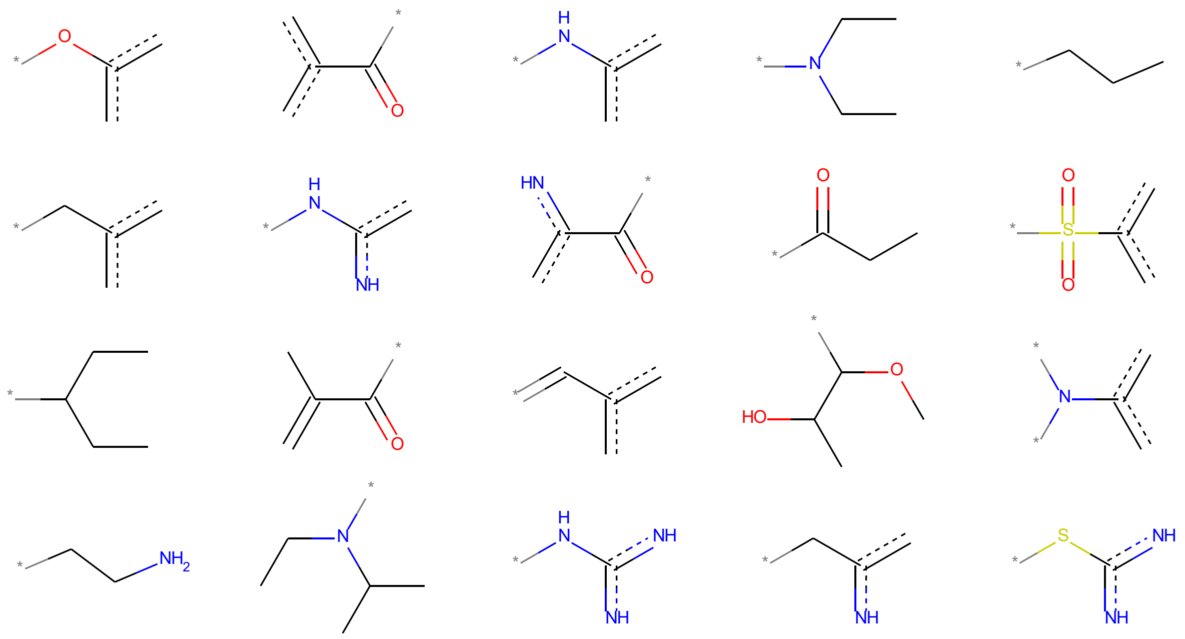

Supplementary Fig.74 Atom environment generated by TSDY_B fragmentation algorithm comes from the training dataset.

TSDY_M Dataset

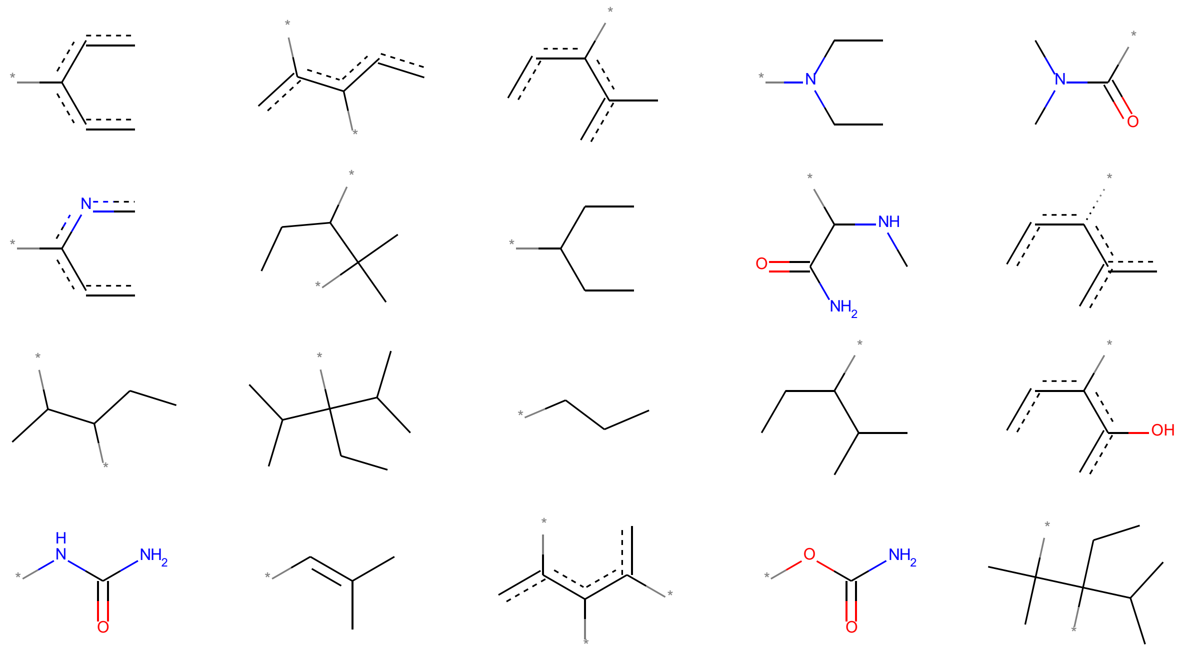

Supplementary Fig.75 Atom environment generated by TSDY_M fragmentation algorithm comes from the training dataset.

TSDY_S Dataset

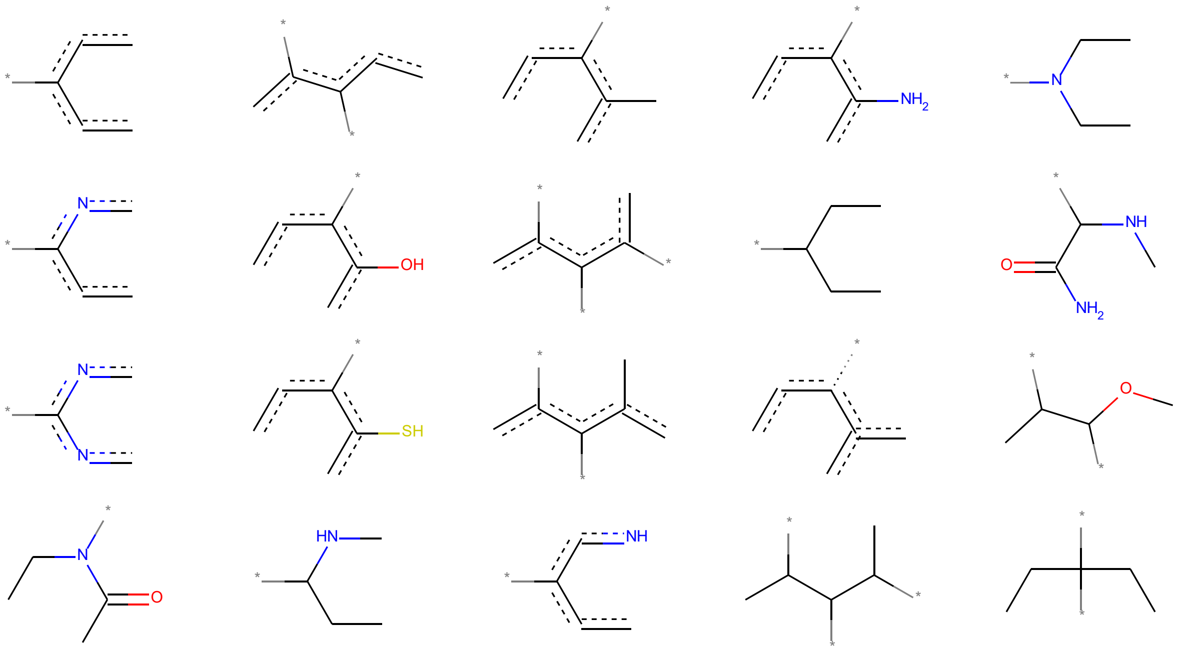

Supplementary Fig.76 Atom environment generated by TSDY_S fragmentation algorithm comes from the training dataset.

TSDY_B Generated_GPT

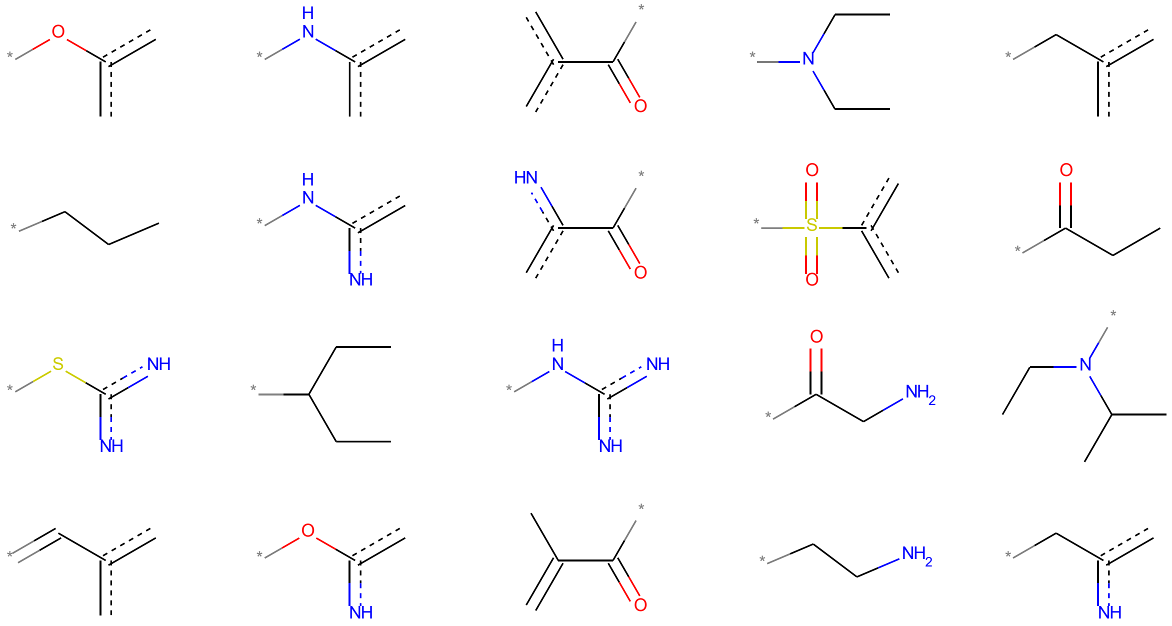

Supplementary Fig.77 Atom environment comes from TSDY_B based models using GPT.

TSDY_B Generated_LSTM

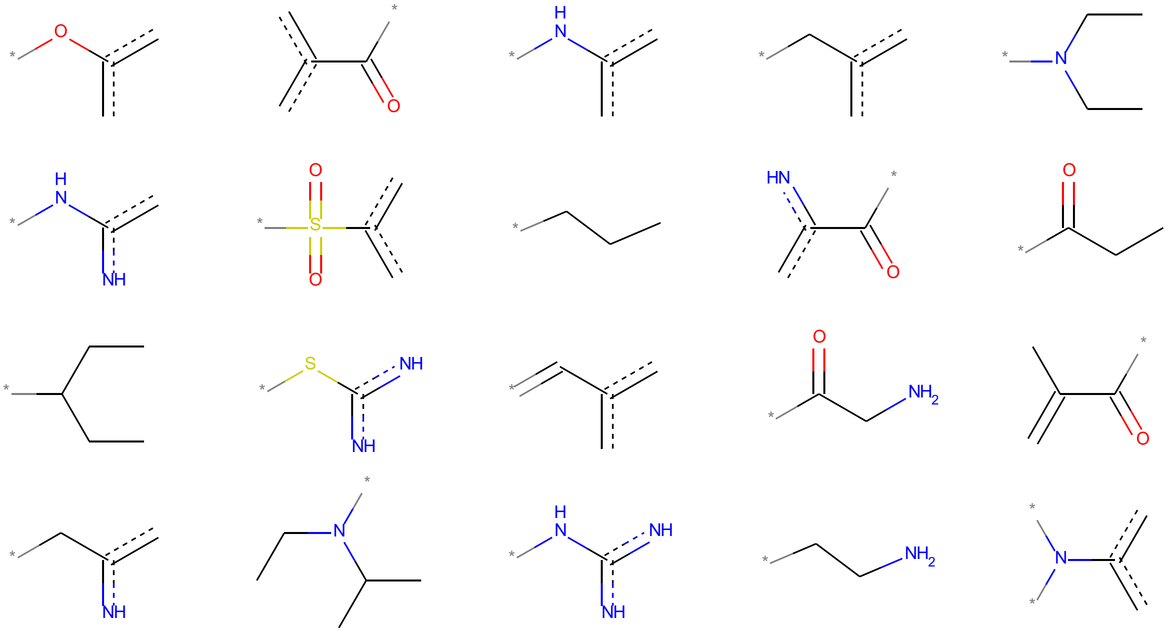

Supplementary Fig.78 Atom environment comes from TSDY_B based models using LSTM.

TSID_B Generated_GPT

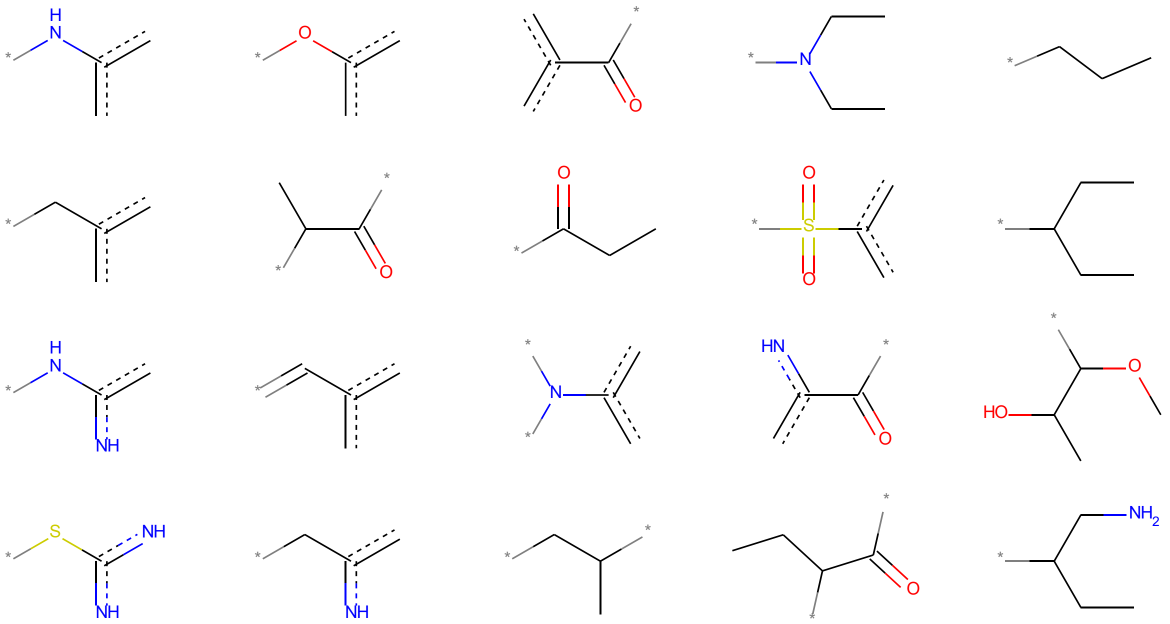

Supplementary Fig.79 Atom environment comes from TSID_B based models using GPT.

TSID_RB Generated_GPT

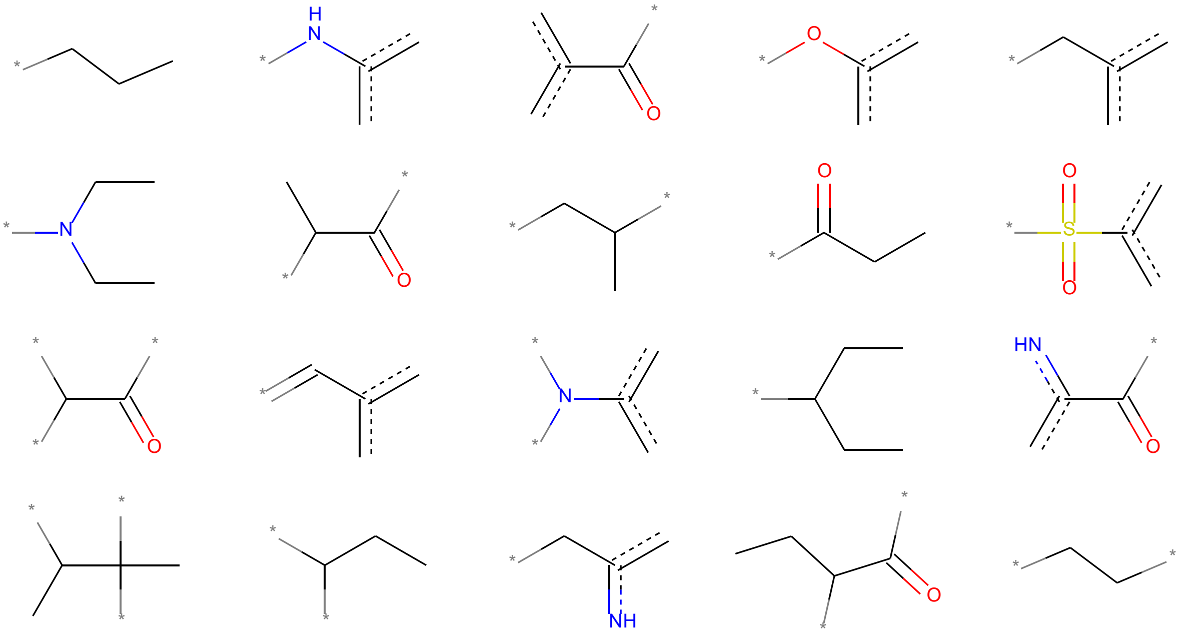

Supplementary Fig.80 Atom environment comes from TSID_RB based models using GPT.

TSDY_M Generated_GPT

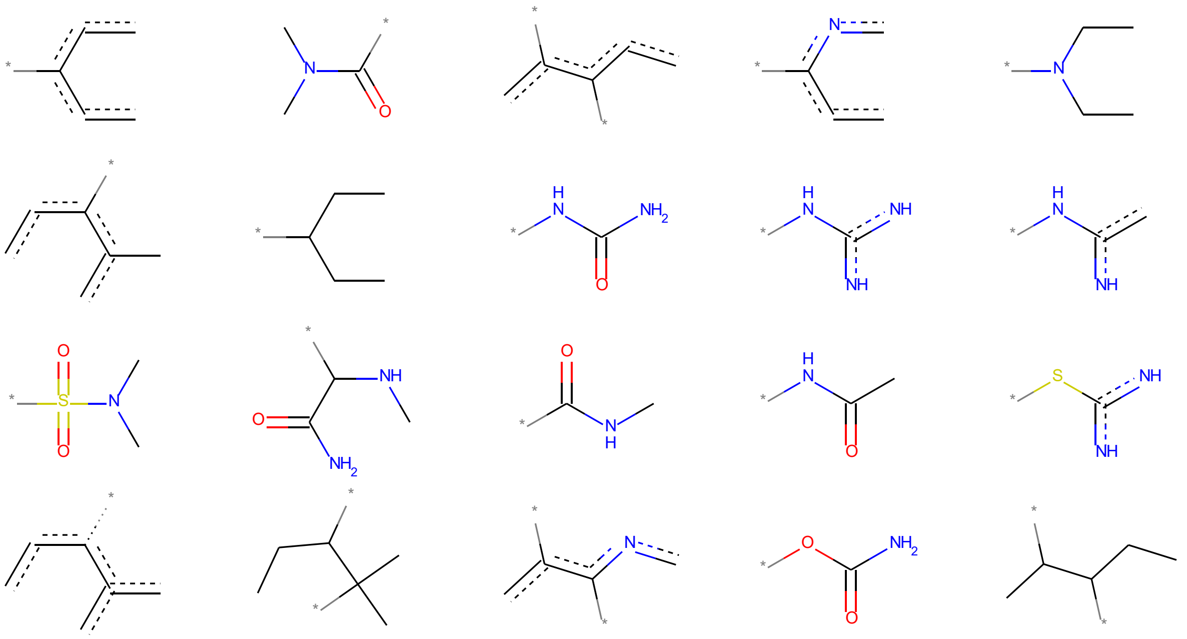

Supplementary Fig.81 Atom environment comes from TSDY_M based models using GPT.

TSDY_M Generated_LSTM

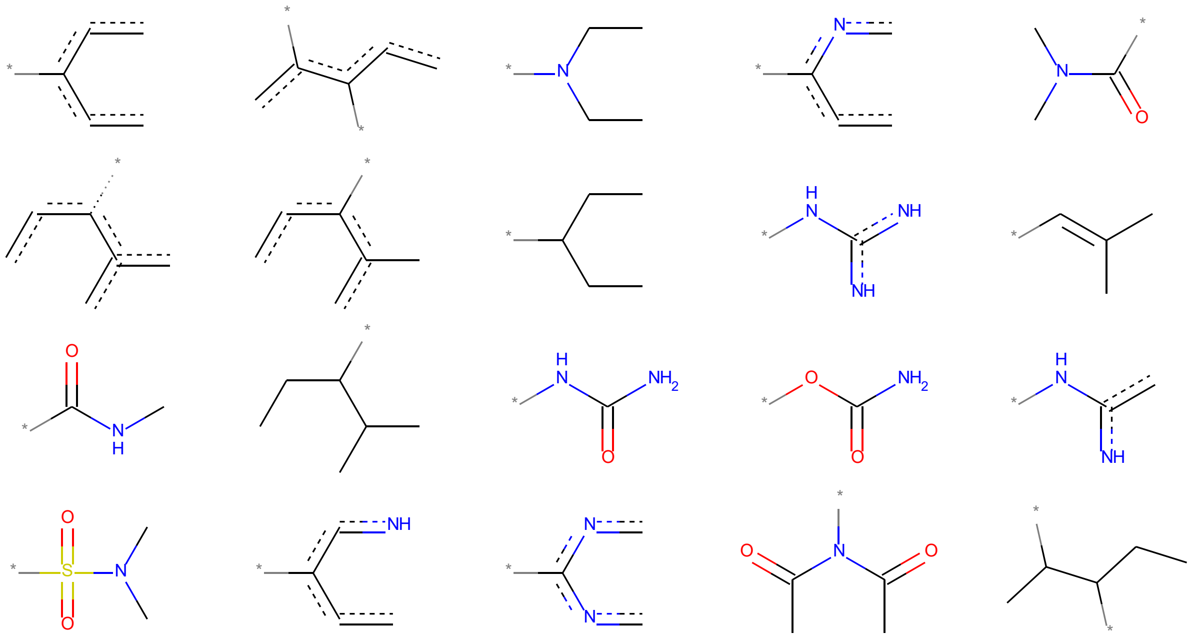

Supplementary Fig.82 Atom environment comes from TSDY_M based models using LSTM.

TSDY_S Generated_GPT

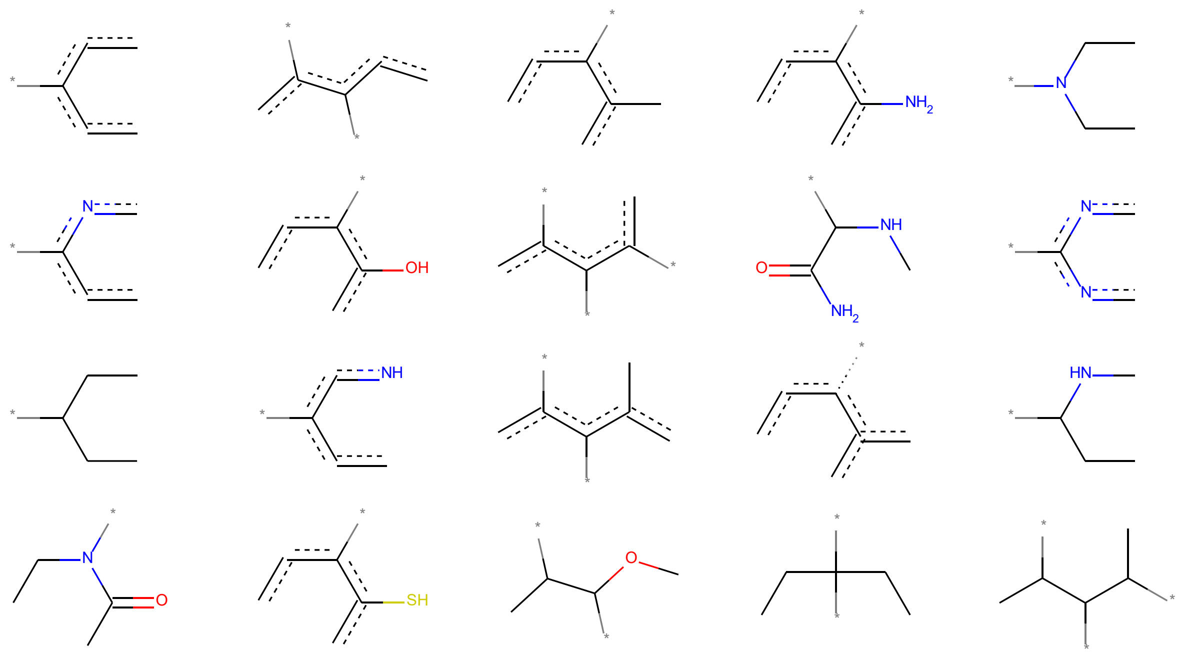

Supplementary Fig.83 Atom environment comes from TSDY_S based models using GPT.

TSDY_S Generated_LSTM

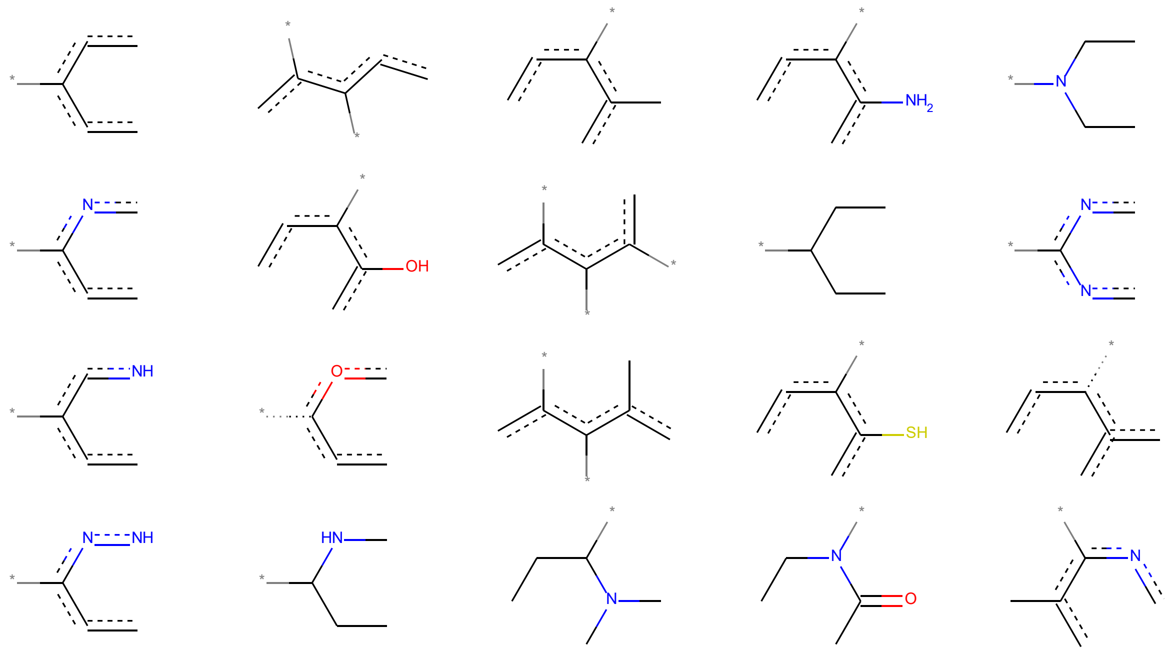

Supplementary Fig.84 Atom environment comes from TSDY_S based models using LSTM.

List of Abbreviations

1. SMILES: Simplified Molecular Input Line Entry Specification
2. t-SMILES: Tree based SMILES
3. TSSA: t-SMILES with shared atom
4. TSDY: t-SMILES with dummy atom but without ID
5. TSID: t-SMILES with ID of dummy atom
6. TS_Vanilla: Classical SMILES in t-SMILES format
7. TS*_J: t-SMILES with JTVAE as fragmentation algorithm
8. TS*_B: t-SMILES with BRICS as fragmentation algorithm
9. TS*_M: t-SMILES with MMPA as fragmentation algorithm
10. TS*_S: t-SMILES with Scaffold as fragmentation algorithm
11. TS*_H*: Hybrid t-SMILES
12. DSMILES: DeepSMILES
13. SELFIES: Self-referencing embedded strings
14. BFS: Breadth First Search
15. DFS: Depth First Search
16. AMT: Acyclic Molecular Tree
17. FBT: full binary tree
18. NLP: Natural Language Processing
19. LLM: Large language model
20. LM: Language Model
21. SOTA: State-of-the-art
22. FBDD: Fragment-based drug discovery
23. PlogP: Penalized logP
24. SAS: Synthetic Accessibility Score
25. QED: Quantitative Estimate of Drug-likeness
26. NPS: Natural Product-likeness score
27. FCD: Fréchet ChemNet Distance
28. KLD: Kullback–Leibler divergence

Supplementary References

1. Jin, W., Barzilay, R. & Jaakkola, T. Junction Tree Variational Autoencoder for Molecular Graph Generation. in *International Conference on Machine Learning* 2323–2332 (PMLR, 2018).
2. Degen, J., Wegscheid-Gerlach, C., Zaliani, A. & Rarey, M. On the art of compiling and using 'drug-like' chemical fragment spaces. *ChemMedChem* **3**, 1503–1507 (2008).
3. Hussain, J. & Rea, C. Computationally efficient algorithm to identify matched molecular pairs (MMPs) in large data sets. *J. Chem. Inf. Model.* **50**, 339–348 (2010).
4. Bemis, G. W. & Murcko, M. A. The properties of known drugs. 1. Molecular frameworks. *J. Med. Chem.* **39**, 2887–2893 (1996).
5. Radhakrishnan, S., Kolippakkam, D. & Mathura, V. S. *Introduction to algorithms*. *Bioinformatics: A Concept-Based Introduction* (MIT press, 2007). doi:10.1007/978-0-387-84870-9_3.
6. Rarey, M. & Dixon, J. S. Feature trees: A new molecular similarity measure based on tree matching. *J. Comput. Aided. Mol. Des.* **12**, 471–490 (1998).
7. Clayden, J., Greeves, N. & Warren, S. G. *Organic chemistry*. *Oxford University Press* vol. 36 (Oxford University Press, 2001).
8. Lehn, J. Supramolecular Chemistry—Scope and Perspectives Molecules, Supermolecules, and Molecular Devices (Nobel Lecture). *Angew. Chemie Int. Ed. English* **27**, 89–112 (1988).
9. Jean-Marie Lehn - Interview. https://www.nobelprize.org/prizes/chemistry/1987/lehn/interview/.
10. Cadeddu, A., Wylie, E. K., Jurczak, J., Wampler-Doty, M. & Grzybowski, B. A. Organic chemistry as a language and the implications of chemical linguistics for structural and retrosynthetic analyses. *Angew. Chemie Int. Ed.* **53**, 8108–8112 (2014).
11. Jinsong, S., Qifeng, J., Xing, C., Hao, Y. & Wang, L. Molecular fragmentation as a crucial step in the AI-based drug development pathway. *Commun. Chem.* **7**, 20 (2024).
12. Liu, T., Naderi, M., Alvin, C., Mukhopadhyay, S. & Brylinski, M. Break Down in Order to Build Up: Decomposing Small Molecules for Fragment-Based Drug Design with eMolFrag. *J. Chem. Inf. Model.* **57**, 627–631 (2017).
13. Lewell, X. Q., Judd, D. B., Watson, S. P. & Hann, M. M. RECAP - Retrosynthetic Combinatorial Analysis Procedure: A powerful new technique for identifying privileged molecular fragments with useful applications in combinatorial chemistry. *J. Chem. Inf. Comput. Sci.* **38**, 511–522 (1998).
14. Leach, A. G. *et al.* Matched Molecular Pairs as a Guide in the Optimization of Pharmaceutical Properties; a Study of Aqueous Solubility, Plasma Protein Binding and Oral Exposure. *J. Med. Chem.* **49**, 6672–6682 (2006).
15. Griffen, E., Leach, A. G., Robb, G. R. & Warner, D. J. Matched Molecular Pairs as a Medicinal Chemistry Tool. *J. Med. Chem.* **54**, 7739–7750 (2011).
16. Tyrchan, C. & Evertsson, E. Matched Molecular Pair Analysis in Short: Algorithms, Applications and Limitations. *Comput. Struct. Biotechnol. J.* **15**, 86–90 (2017).
17. Lim, J., Hwang, S. Y., Moon, S., Kim, S. & Kim, W. Y. Scaffold-based molecular design with a graph generative model. *Chem. Sci.* **11**, 1153–1164 (2020).
18. Singh, S. & Mahmood, A. The NLP Cookbook: Modern Recipes for Transformer Based Deep Learning Architectures. *IEEE Access* **9**, 68675–68702 (2021).
19. Hochreiter, S. & Schmidhuber, J. Long Short-Term Memory. *Neural Comput.* **9**, 1735–1780 (1997).
20. Cho, K., van Merriënboer, B., Bahdanau, D. & Bengio, Y. On the properties of neural machine translation: Encoder–decoder approaches. *Proc. SSST 2014 - 8th Work. Syntax. Semant. Struct. Stat. Transl.* 103–111 (2014) doi:10.3115/v1/w14-4012.
21. Vaswani, A. *et al.* Attention is all you need. in *Advances in neural information processing systems 30* (2017).
22. Radford, A., Narasimhan, K., Salimans, T. & Sutskever, I. Improving Language Understanding by Generative Pre-Training. *Homol. Homotopy Appl.* (2018).
23. Alec Radford, Jeffrey Wu, Rewon Child, David Luan, Dario Amodei, I. S. Language Models are Unsupervised Multitask Learners. *OpenAI* **1**, 1–7 (2020).
24. Brown, T. B. *et al.* Language models are few-shot learners. *Adv. Neural Inf. Process. Syst.* **2020-Decem**, (2020).

25. Devlin, J., Chang, M. W., Lee, K. & Toutanova, K. BERT: Pre-training of deep bidirectional transformers for language understanding. *NAACL HLT 2019 - 2019 Conf. North Am. Chapter Assoc. Comput. Linguist. Hum. Lang. Technol. - Proc. Conf.* **1**, 4171–4186 (2019).
26. Bagal, V., Aggarwal, R., Vinod, P. K. & Priyakumar, U. D. MolGPT: Molecular Generation Using a Transformer-Decoder Model. *J. Chem. Inf. Model.* **62**, 2064–2076 (2021).
27. Podda, M., Bacciu, D. & Micheli, A. A deep generative model for fragment-based molecule generation. in *International conference on artificial intelligence and statistics* 2240–2250 (PMLR, 2020).
28. Hu, Y., Hu, Y. & Cen, E. HierVAE++: An Update of Hierarchical Generation of Molecular Graphs Using Structural Motifs. *Proc. - 2021 2nd Int. Conf. Big Data Artif. Intell. Softw. Eng.* (2021).
29. Polishchuk, P. CReM: chemically reasonable mutations framework for structure generation. *J. Cheminform.* **12**, 28 (2020).
30. De Cao, N. & Kipf, T. MolGAN: An implicit generative model for small molecular graphs. *ArXiv* (2018).
31. Mahmood, O., Mansimov, E., Bonneau, R. & Cho, K. Masked graph modeling for molecule generation. *Nat. Commun.* **12**, (2021).
32. Ip, Y. T. & Davis, R. J. Signal transduction by the c-Jun N-terminal kinase (JNK) - From inflammation to development. *Curr. Opin. Cell Biol.* **10**, 205–219 (1998).
33. AID 1706 - QFRET-based primary biochemical high throughput screening assay to identify inhibitors of the SARS coronavirus 3C-like Protease (3CLPro) - PubChem. https://pubchem.ncbi.nlm.nih.gov/bioassay/1706.
34. Gaulton, A. *et al.* ChEMBL: A large-scale bioactivity database for drug discovery. *Nucleic Acids Res.* **40**, (2012).
35. Sterling, T. & Irwin, J. J. ZINC 15 - Ligand Discovery for Everyone. *J. Chem. Inf. Model.* **55**, 2324–2337 (2015).
36. Ramakrishnan, R., Dral, P. O., Rupp, M. & Von Lilienfeld, O. A. Quantum chemistry structures and properties of 134 kilo molecules. *Sci. Data* **1**, 1–7 (2014).
37. Brown, N., Fiscato, M., Segler, M. H. S. & Vaucher, A. C. GuacaMol: Benchmarking Models for de Novo Molecular Design. *J. Chem. Inf. Model.* **59**, 1096–1108 (2019).
38. Polykovskiy, D. *et al.* Molecular Sets (MOSES): A Benchmarking Platform for Molecular Generation Models. *Front. Pharmacol.* **11**, (2020).
39. Renz, P., Van Rompaey, D., Wegner, J. K., Hochreiter, S. & Klambauer, G. On failure modes in molecule generation and optimization. *Drug Discov. Today Technol.* **32**–**33**, 55–63 (2019).
40. Kullback, S. & Leibler, R. A. On Information and Sufficiency. *Ann. Math. Stat.* **22**, 79–86 (1951).
41. Preuer, K., Renz, P., Unterthiner, T., Hochreiter, S. & Klambauer, G. Fréchet ChemNet Distance: A Metric for Generative Models for Molecules in Drug Discovery. *J. Chem. Inf. Model.* **58**, 1736–1741 (2018).
42. Landrum, G. RDKit : A software suite for cheminformatics , computational chemistry , and predictive modeling. *RDKit A Softw. Suite Cheminformatics, Comput. Chem. Predict. Model.* (2013).
43. Goh, G. B., Siegel, C., Vishnu, A. & Hodas, N. O. ChemNet: A Transferable and Generalizable Deep Neural Network for Small-Molecule Property Prediction. *arXiv* **abs/1712.0**, 1712.02734 (2017).
44. Rajasekar, A. A., Raman, K. & Ravindran, B. Goal directed molecule generation using Monte Carlo Tree Search. *ArXiv* **abs/2010.1**, (2020).
45. Ertl, P. & Schuffenhauer, A. Estimation of synthetic accessibility score of drug-like molecules based on molecular complexity and fragment contributions. *J. Cheminform.* **1**, (2009).
46. Bertz, S. H. The First General Index of Molecular Complexity. *J. Am. Chem. Soc.* **103**, 3599–3601 (1981).
47. Bickerton, G. R., Paolini, G. V., Besnard, J., Muresan, S. & Hopkins, A. L. Quantifying the chemical beauty of drugs. *Nat. Chem.* **4**, 90–98 (2012).
48. Ertl, P., Roggo, S. & Schuffenhauer, A. Natural product-likeness score and its application for prioritization of compound libraries. *J. Chem. Inf. Model.* **48**, 68–74 (2008).
49. Virtanen, P. *et al.* SciPy 1.0: fundamental algorithms for scientific computing in Python. *Nat. Methods* **17**, 261–272 (2020).
50. Naderi, M., Alvin, C., Ding, Y., Mukhopadhyay, S. & Brylinski, M. A graph-based approach to construct target-focused libraries for virtual screening. *J. Cheminform.* **8**, 1–16 (2016).
51. Lipinski, C. A., Lombardo, F., Dominy, B. W. & Feeney, P. J. Experimental and computational approaches to estimate solubility and permeability in drug discovery and development settings. *Adv. Drug Deliv. Rev.* **23**, 3–25 (1997).

52. Gómez-Bombarelli, R. *et al.* Automatic Chemical Design Using a Data-Driven Continuous Representation of Molecules. *ACS Cent. Sci.* **4**, 268–276 (2018).
53. Simonovsky, M. & Komodakis, N. GraphVAE: Towards generation of small graphs using variational autoencoders. *Lect. Notes Comput. Sci. (including Subser. Lect. Notes Artif. Intell. Lect. Notes Bioinformatics)* **11139 LNCS**, 412–422 (2018).
54. Zhang, L., Rao, V. & Cornell, W. r-BRICS – a revised BRICS module that breaks ring structures and carbon chains. *ChemMedChem* e202300202 (2023) doi:10.1002/cmdc.202300202.
