# Supplementary material for: t-SMILES: a fragment-based molecular representation framework for de novo ligand design
Source: Nat Commun. 2024 Jun 11;15:4993. doi: 10.1038/s41467-024-49388-6 (PMC11167009; doi:10.1038/s41467-024-49388-6)
Supplement: Supplementary file 4 — Source Data [file 41467_2024_49388_MOESM4_ESM.zip › Soure data/Source data and code for all other graphs/Metrics/Figs/Supplementary Fig. 1_TSSA.pdf]

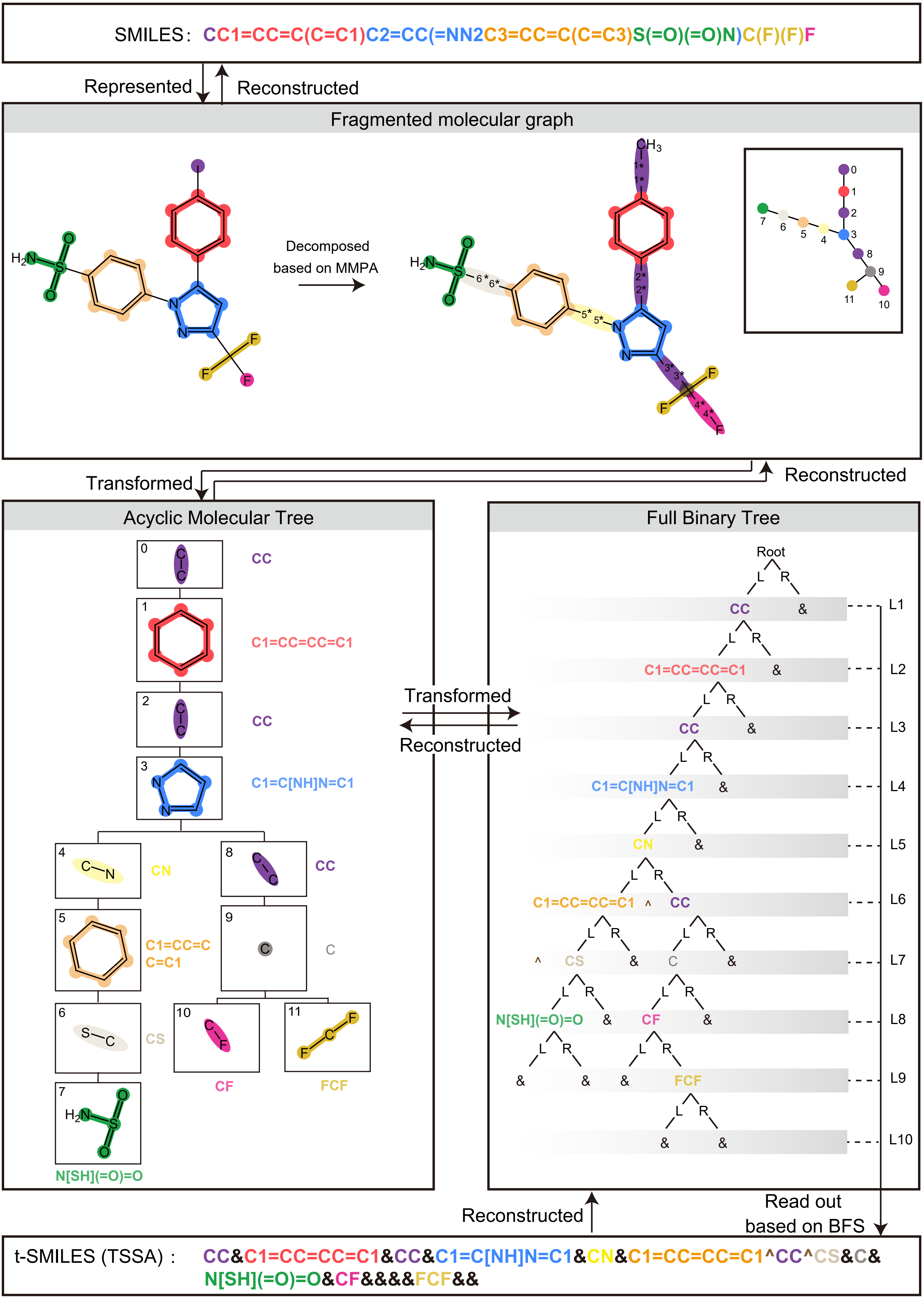
SMILES: CC1=CC=C(C=C1)C2=CC(=NN2C3=CC=C(C=C3)S(=O)(=O)N)C(F)(F)F
Represented
Reconstructed
Fragmented molecular graph
Decomposed
based on MMPA
Transformed
Reconstructed
Acyclic Molecular Tree
CC
C1=CC=CC=C1
CC
C1=C[NH]N=C1
CN
C1=CC=C
C=C1
CS
N[SH](=O)=O
CC
C
CF
FCF
Transformed
Reconstructed
Full Binary Tree
Root
L1
L2
L3
L4
L5
L6
L7
L8
L9
L10
Reconstructed
Read out
based on BFS
t-SMILES (TSSA) : CC&C1=CC=CC=C1&CC&C1=C[NH]N=C1&CN&C1=CC=CC=C1^CC^CS&C&
N[SH](=O)=O&CF&&&&FCF&&
