# Supplementary figures and images for: t-SMILES: a fragment-based molecular representation framework for de novo ligand design
Source: Nat Commun. 2024 Jun 11;15:4993. doi: 10.1038/s41467-024-49388-6 (PMC11167009; doi:10.1038/s41467-024-49388-6)

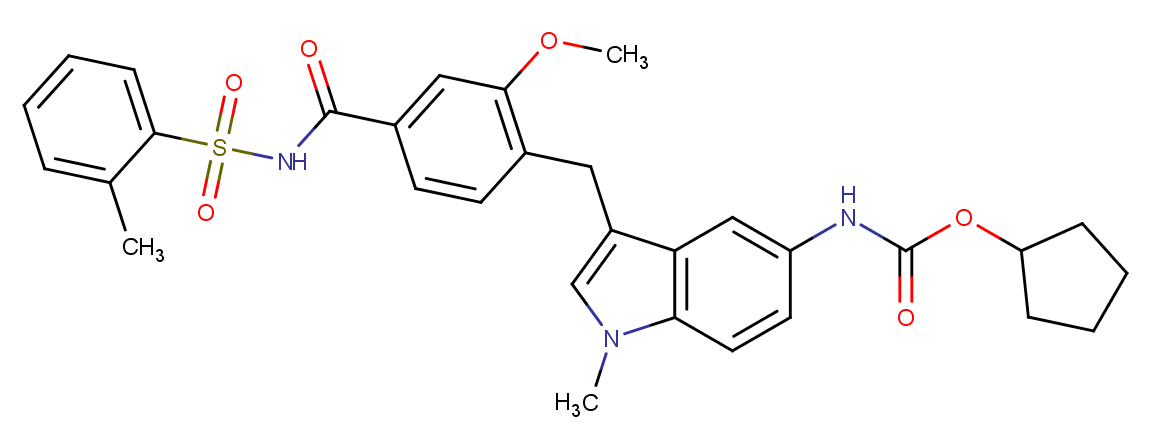

Supplement: Supplementary file 4 — Source Data [file 41467_2024_49388_MOESM4_ESM.zip › Soure data/Source data and code for all other graphs/Metrics/Dataset/AID1706/Active/onemol/1/onemol.png]

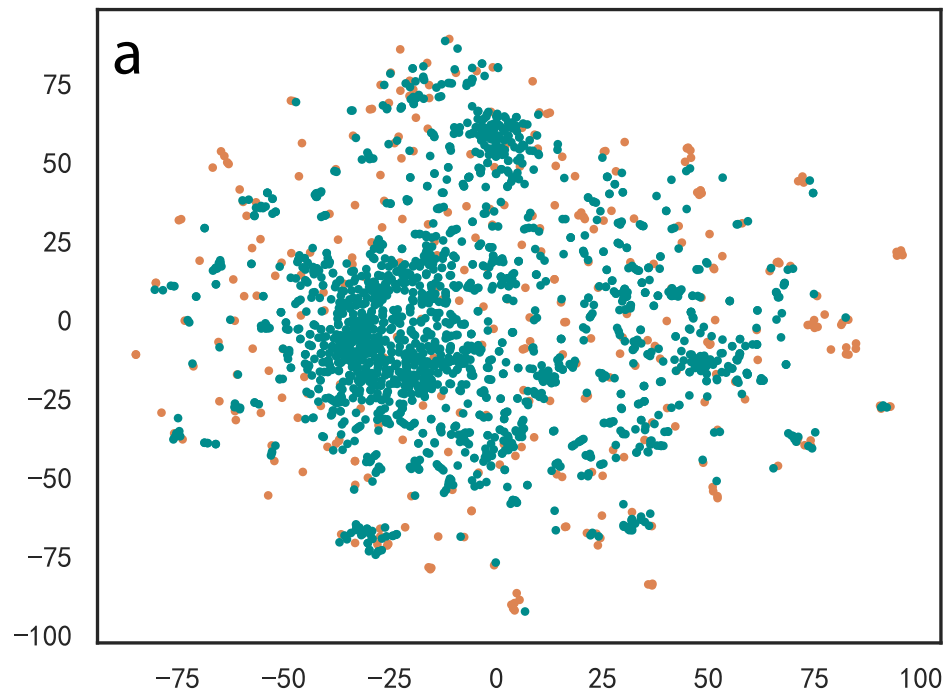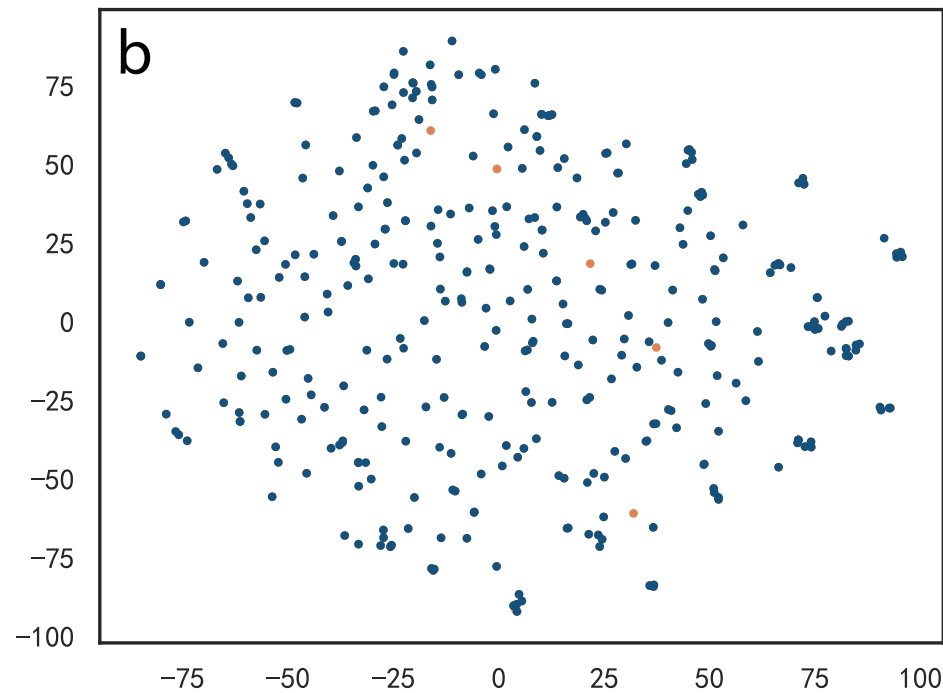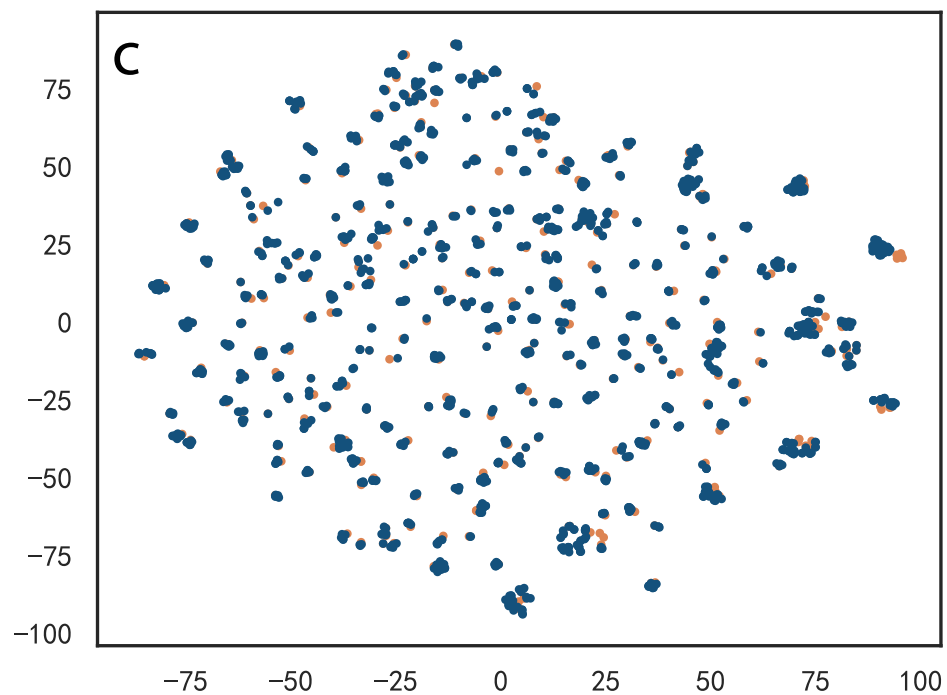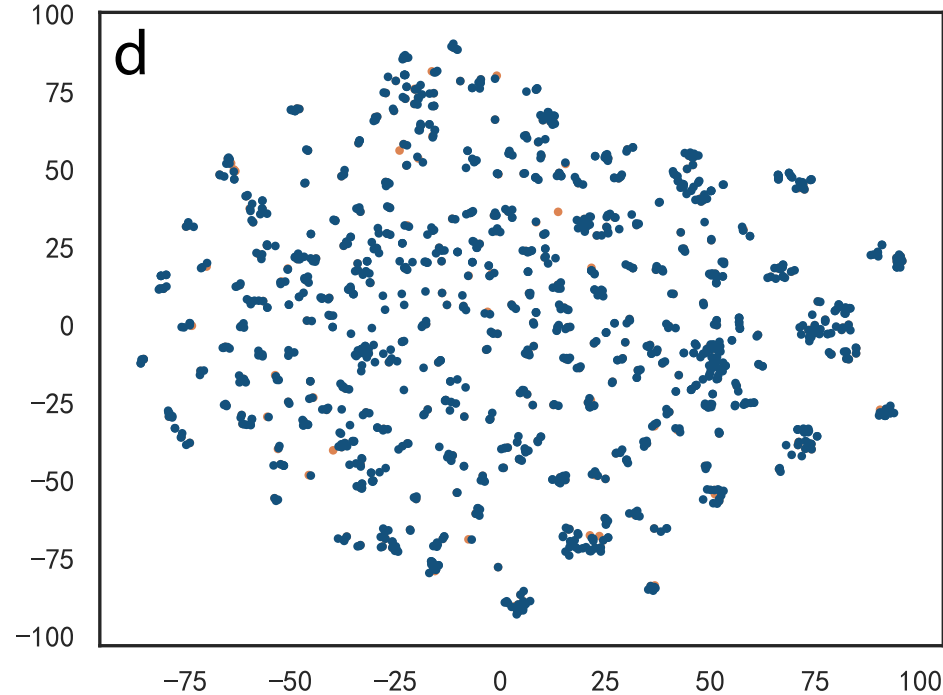

a: Active vs Inactive, b: Active vs SMILES, c: Active vs TSSA\_S, d:Active vs H6

Supplement: Supplementary file 4 — Source Data [file 41467_2024_49388_MOESM4_ESM.zip › Soure data/Source data and code for all other graphs/Metrics/Models/AID1706/AID_H6[dbl][rnd1000][bs128]/[TSNE]_[FP_Topological].pdf]

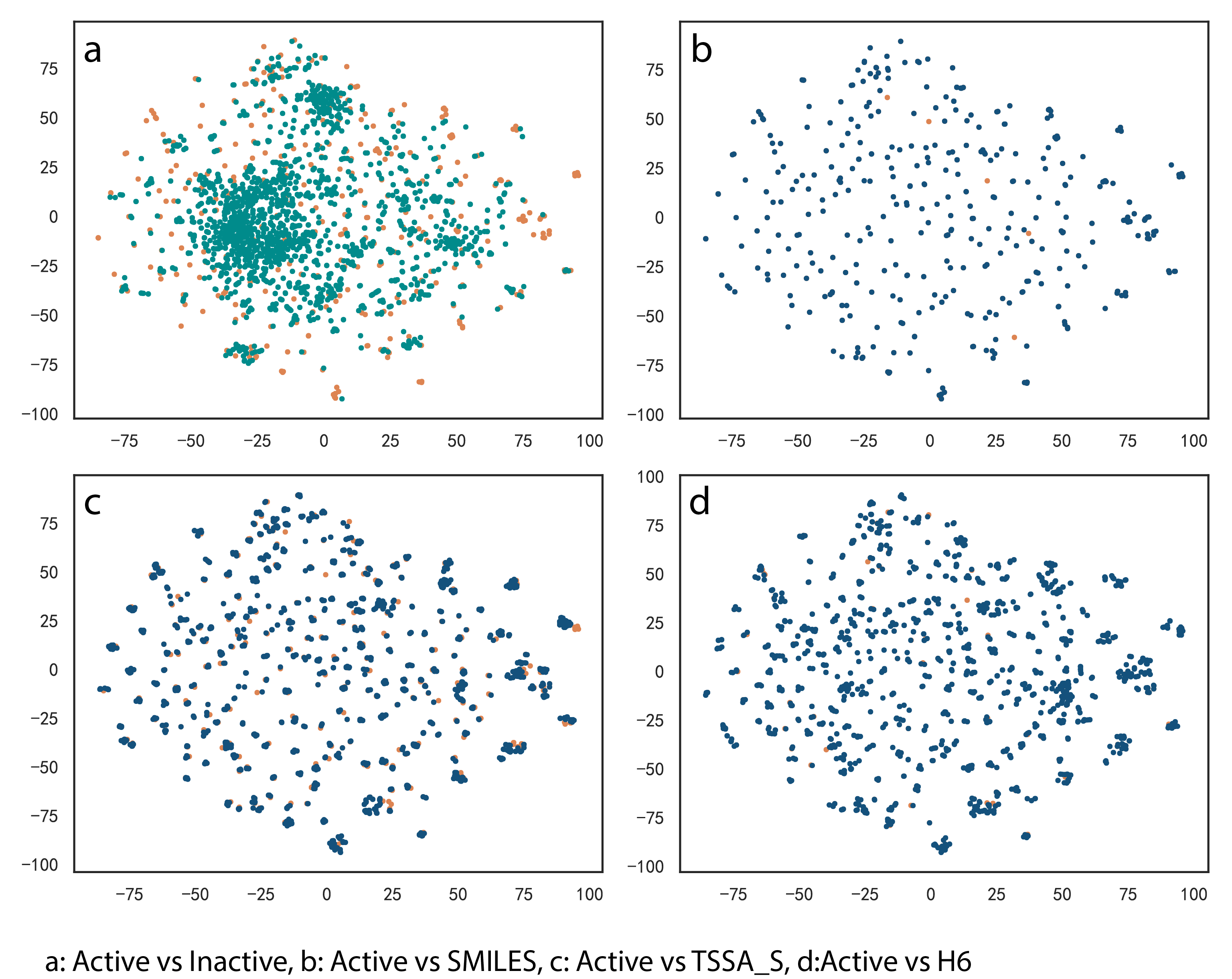

Supplement: Supplementary file 4 — Source Data [file 41467_2024_49388_MOESM4_ESM.zip › Soure data/Source data and code for all other graphs/Metrics/Models/AID1706/AID_H6[dbl][rnd1000][bs128]/[TSNE]_[FP_Topological].png]
