# Supplementary material for: t-SMILES: a fragment-based molecular representation framework for de novo ligand design
Source: Nat Commun. 2024 Jun 11;15:4993. doi: 10.1038/s41467-024-49388-6 (PMC11167009; doi:10.1038/s41467-024-49388-6)
Supplement: Supplementary file 4 — Source Data [file 41467_2024_49388_MOESM4_ESM.zip › Soure data/Source data and code for all other graphs/Metrics/Models/JNK3/jnk3_Scaffold[dbl][rnd1000][bs128]/['Act', 'In','S','DS', 'SELF', 'Sc', 'MMPA', 'B', 'J','Sc_TF']/gen.smi.re_smils.smi.pred[19999_17778].csv_new_cmol.smi_[TSNE]_[FP_Topological].pdf]

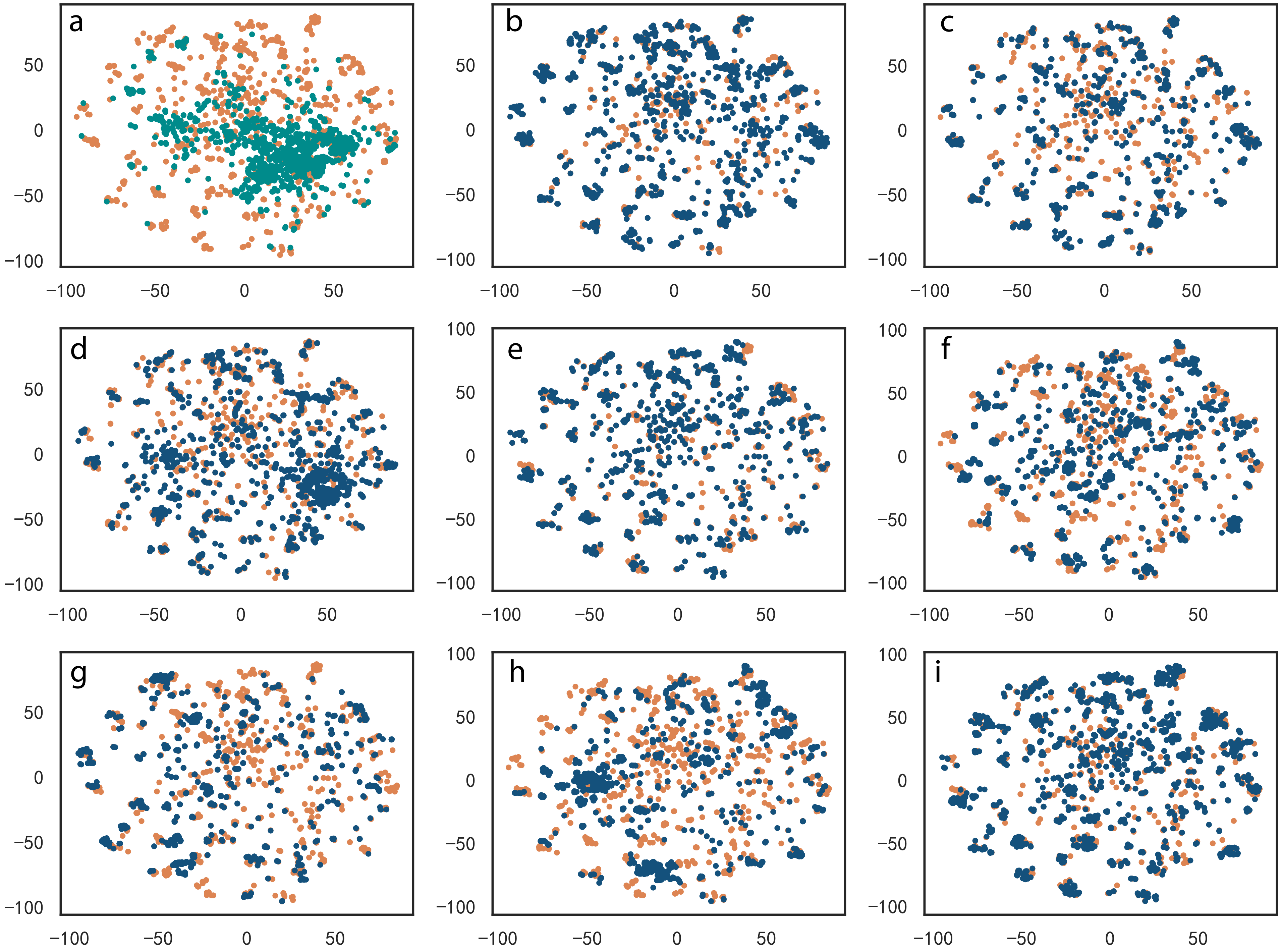

a: Inactive, b: SMILES, c:DeepSMILES, d:SELFIES, e:TSSA_S, f:TSSA_M, g:TSSA_B, h:TSSA_J
i: Pre-trained then fine-tuned model based on TSSA_S
