# Supplementary material for: t-SMILES: a fragment-based molecular representation framework for de novo ligand design
Source: Nat Commun. 2024 Jun 11;15:4993. doi: 10.1038/s41467-024-49388-6 (PMC11167009; doi:10.1038/s41467-024-49388-6)
Supplement: Supplementary file 4 — Source Data [file 41467_2024_49388_MOESM4_ESM.zip › Soure data/Source data and code for all other graphs/Metrics/Models/SAScore/ChemSingle/gen.smi.re_smils[1].smi.rnd10000.csv_SAScore.pdf]

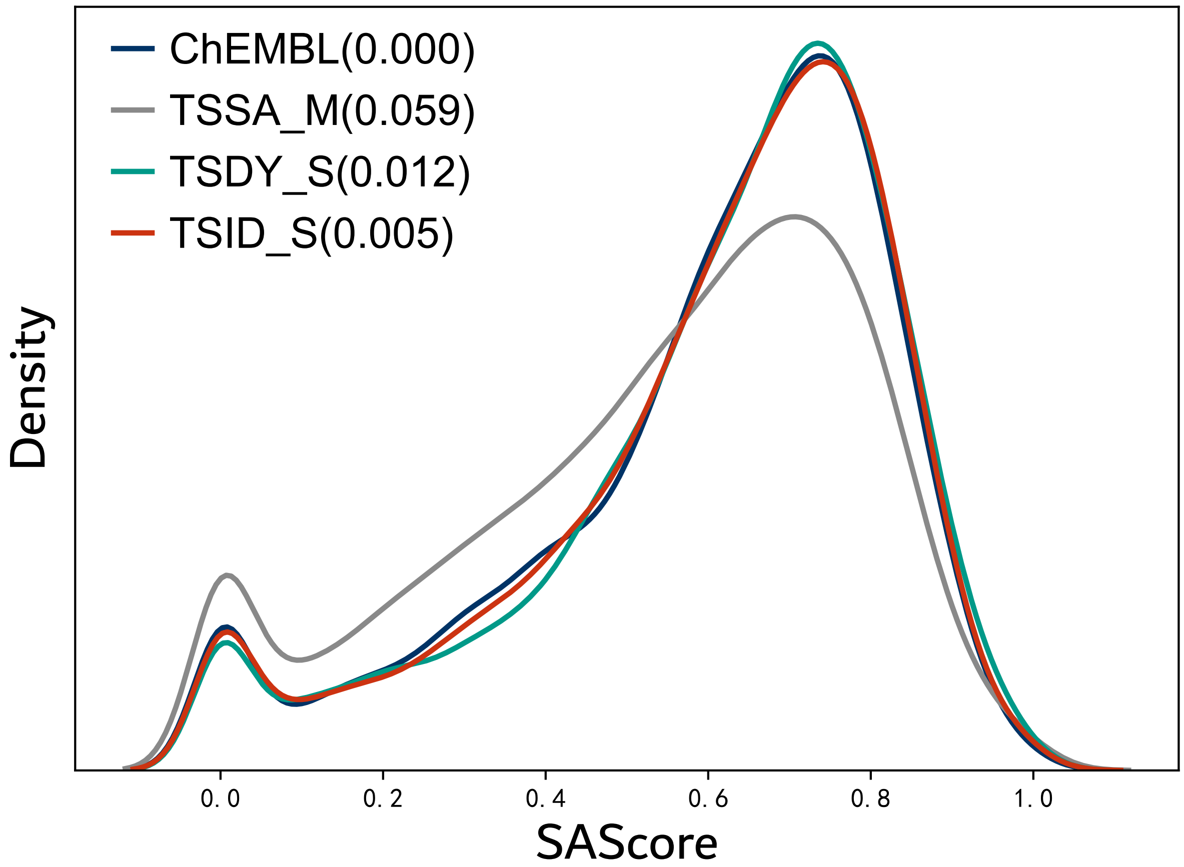

ChEMBL(0.000)
TSSA_M(0.059)
TSDY_S(0.012)
TSID_S(0.005)
Density
0.0
0.2
0.4
0.6
0.8
1.0
SAScore
