# Supplementary material for: t-SMILES: a fragment-based molecular representation framework for de novo ligand design
Source: Nat Commun. 2024 Jun 11;15:4993. doi: 10.1038/s41467-024-49388-6 (PMC11167009; doi:10.1038/s41467-024-49388-6)
Supplement: Supplementary file 4 — Source Data [file 41467_2024_49388_MOESM4_ESM.zip › Soure data/Source data and code for all other graphs/Metrics/Models/SAScore/Untitled-1.pdf]

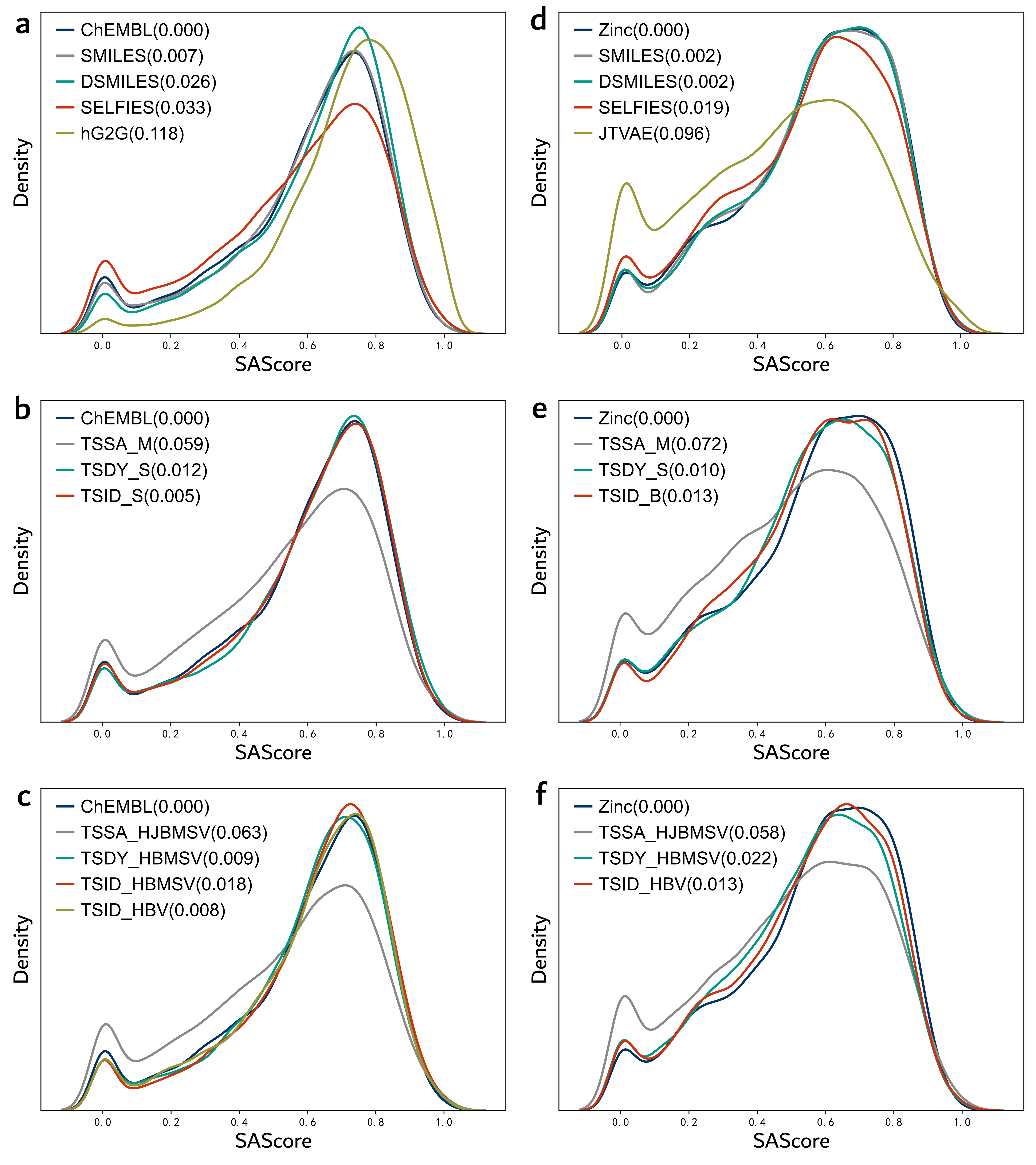

a
ChEMBL(0.000)
SMILES(0.007)
DSMILES(0.026)
SELFIES(0.033)
hG2G(0.118)
Density
SAScore
d
Zinc(0.000)
SMILES(0.002)
DSMILES(0.002)
SELFIES(0.019)
JTVAE(0.096)
Density
SAScore
b
ChEMBL(0.000)
TSSA_M(0.059)
TSDY_S(0.012)
TSID_S(0.005)
Density
SAScore
e
Zinc(0.000)
TSSA_M(0.072)
TSDY_S(0.010)
TSID_B(0.013)
Density
SAScore
c
ChEMBL(0.000)
TSSA_HJBMSV(0.063)
TSDY_HBMSV(0.009)
TSID_HBMSV(0.018)
TSID_HBV(0.008)
Density
SAScore
f
Zinc(0.000)
TSSA_HJBMSV(0.058)
TSDY_HBMSV(0.022)
TSID_HBV(0.013)
Density
SAScore
0.0
0.2
0.4
0.6
0.8
1.0
