# Supplementary material for: t-SMILES: a fragment-based molecular representation framework for de novo ligand design
Source: Nat Commun. 2024 Jun 11;15:4993. doi: 10.1038/s41467-024-49388-6 (PMC11167009; doi:10.1038/s41467-024-49388-6)
Supplement: Supplementary file 4 — Source Data [file 41467_2024_49388_MOESM4_ESM.zip › Soure data/Source data and code for all other graphs/Metrics/Models/Zinc/random/dsifo/random_2.smi.rnd10000.csv_N_Atoms.pdf]

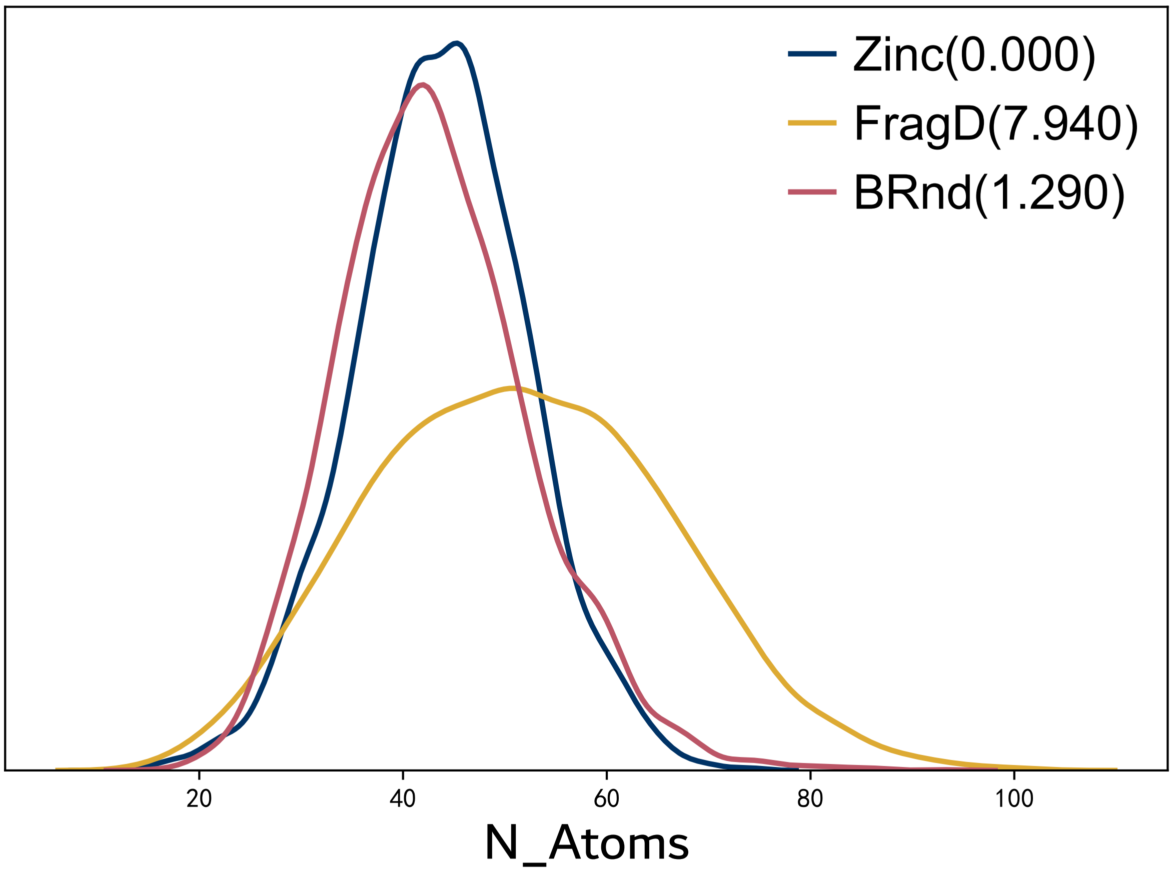
Zinc(0.000)
FragD(7.940)
BRnd(1.290)
20
40
60
80
100
N_Atoms
