# Supplementary material for: t-SMILES: a fragment-based molecular representation framework for de novo ligand design
Source: Nat Commun. 2024 Jun 11;15:4993. doi: 10.1038/s41467-024-49388-6 (PMC11167009; doi:10.1038/s41467-024-49388-6)
Supplement: Supplementary file 4 — Source Data [file 41467_2024_49388_MOESM4_ESM.zip › Soure data/Source data and code for all other graphs/Metrics/Models/Zinc/random/dsifo/random_2.smi.rnd10000.csv_TPSA.pdf]

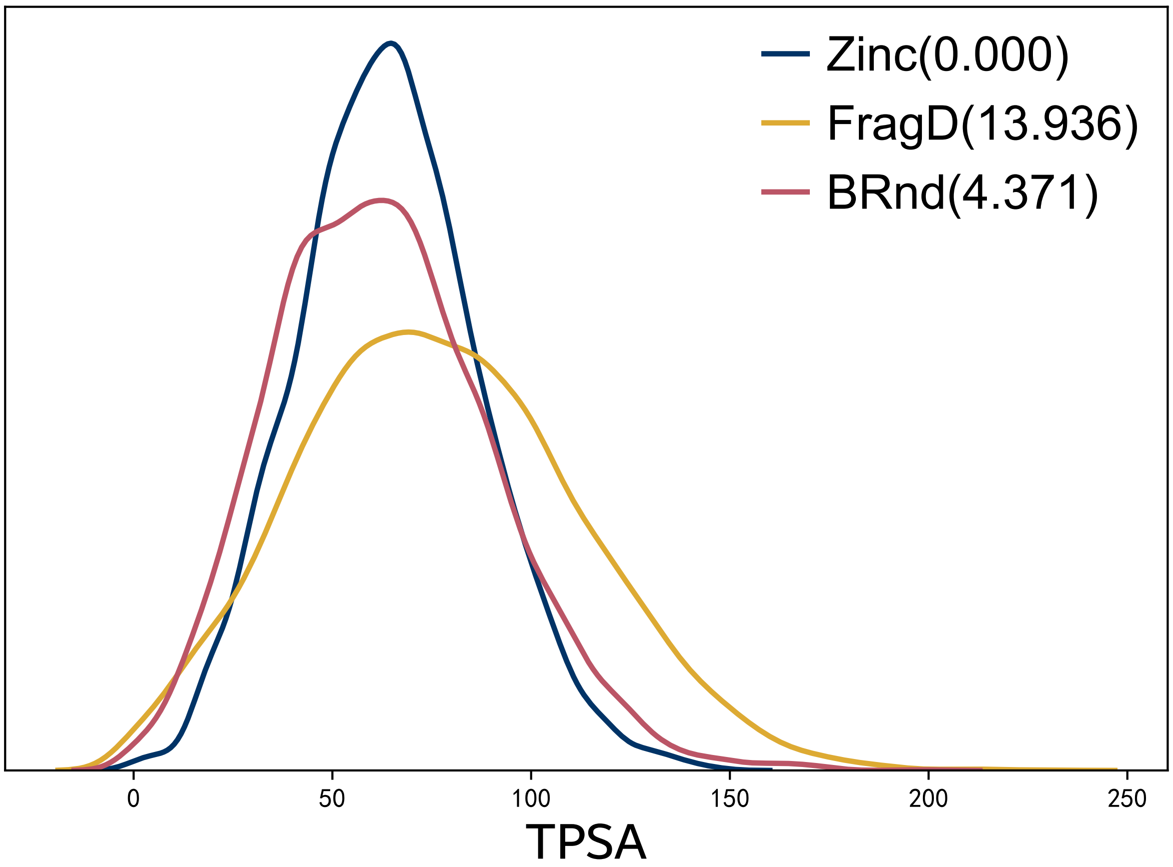

Zinc(0.000)
FragD(13.936)
BRnd(4.371)
0
50
100
150
200
250
TPSA
