# Supplementary material for: t-SMILES: a fragment-based molecular representation framework for de novo ligand design
Source: Nat Commun. 2024 Jun 11;15:4993. doi: 10.1038/s41467-024-49388-6 (PMC11167009; doi:10.1038/s41467-024-49388-6)
Supplement: Supplementary file 4 — Source Data [file 41467_2024_49388_MOESM4_ESM.zip › Soure data/Source data and code for all other graphs/Metrics/Models/Zinc/random/dsifo/Untitled-1.pdf]

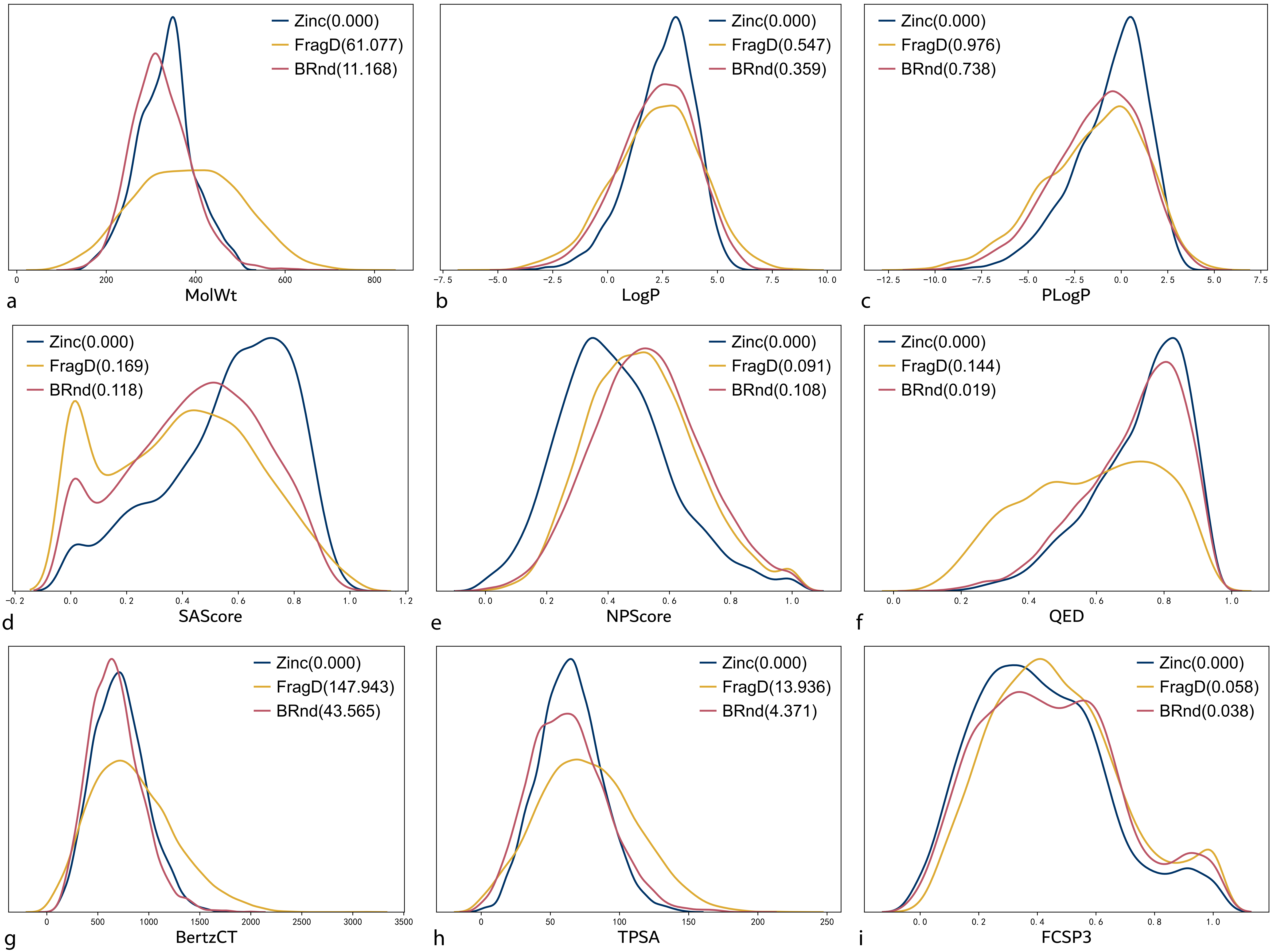
Zinc(0.000)
FragD(61.077)
BRnd(11.168)
a
MolWt
Zinc(0.000)
FragD(0.547)
BRnd(0.359)
b
LogP
Zinc(0.000)
FragD(0.976)
BRnd(0.738)
c
PLogP
Zinc(0.000)
FragD(0.169)
BRnd(0.118)
d
SAScore
Zinc(0.000)
FragD(0.091)
BRnd(0.108)
e
NPScore
Zinc(0.000)
FragD(0.144)
BRnd(0.019)
f
QED
Zinc(0.000)
FragD(147.943)
BRnd(43.565)
g
BertzCT
Zinc(0.000)
FragD(13.936)
BRnd(4.371)
h
TPSA
Zinc(0.000)
FragD(0.058)
BRnd(0.038)
i
FCSP3
