# Supplementary material for: t-SMILES: a fragment-based molecular representation framework for de novo ligand design
Source: Nat Commun. 2024 Jun 11;15:4993. doi: 10.1038/s41467-024-49388-6 (PMC11167009; doi:10.1038/s41467-024-49388-6)
Supplement: Supplementary file 4 — Source Data [file 41467_2024_49388_MOESM4_ESM.zip › Soure data/Soure data for Table, Main Text And Curvers/Fig.1 t-SMILES.pdf]

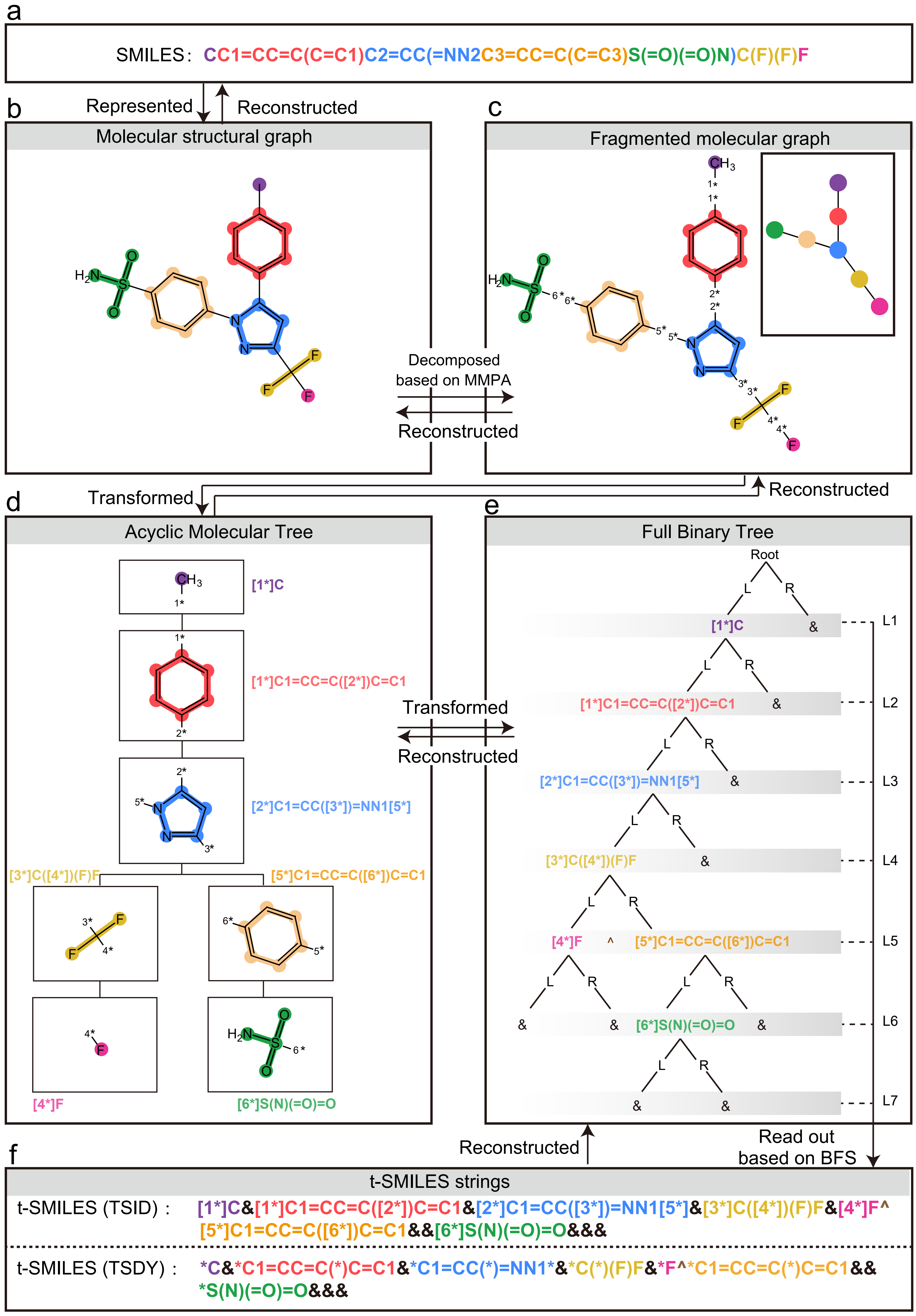
a
SMILES: CC1=CC=C(C=C1)C2=CC(=NN2C3=CC=C(C=C3)S(=O)(=O)N)C(F)(F)F
Represented
Reconstructed
b
Molecular structural graph
c
Fragmented molecular graph
Decomposed based on MMPA
Reconstructed
Transformed
Reconstructed
d
Acyclic Molecular Tree
[1*]C
[1*]C1=CC=C([2*])C=C1
[2*]C1=CC([3*])=NN1[5*]
[3*]C([4*])(F)F
[5*]C1=CC=C([6*])C=C1
[4*]F
[6*]S(N)(=O)=O
Transformed
Reconstructed
e
Full Binary Tree
Root
L1
L2
L3
L4
L5
L6
L7
Reconstructed
Read out based on BFS
f
t-SMILES strings
t-SMILES (TSID) : [1*]C&[1*]C1=CC=C([2*])C=C1&[2*]C1=CC([3*])=NN1[5*]&[3*]C([4*])(F)F&[4*]F^[5*]C1=CC=C([6*])C=C1&&[6*]S(N)(=O)=O&&&
t-SMILES (TSDY) : *C&*C1=CC=C(*)C=C1&*C1=CC(*)=NN1*&*C(*)(F)F&*F^*C1=CC=C(*)C=C1&&*S(N)(=O)=O&&&
