# Supplementary material for: t-SMILES: a fragment-based molecular representation framework for de novo ligand design
Source: Nat Commun. 2024 Jun 11;15:4993. doi: 10.1038/s41467-024-49388-6 (PMC11167009; doi:10.1038/s41467-024-49388-6)
Supplement: Supplementary file 4 — Source Data [file 41467_2024_49388_MOESM4_ESM.zip › Soure data/Soure data for Table, Main Text And Curvers/Fig.2 Tokens/Fig.2 Token.pdf]

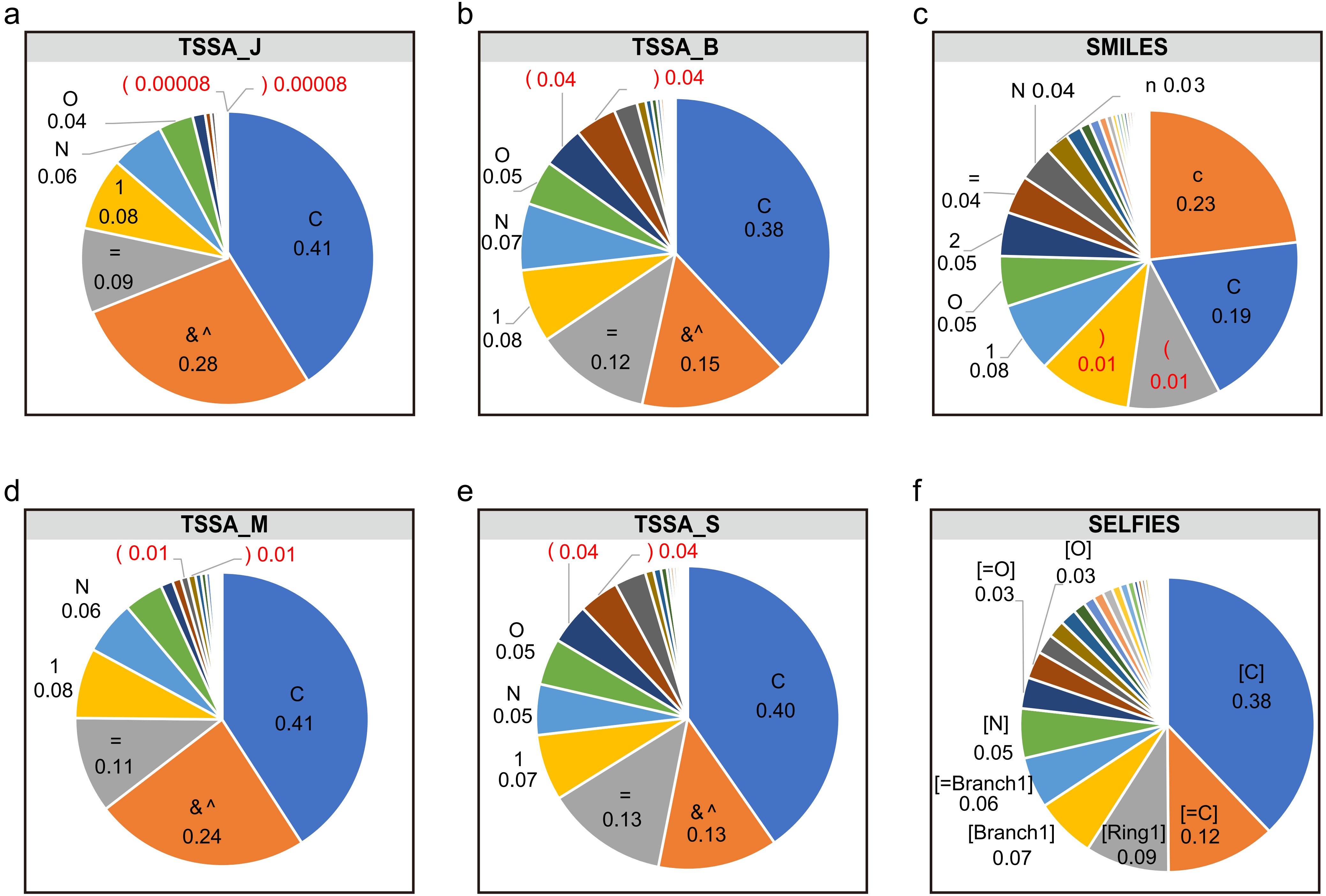

a
TSSA_J
(0.00008
) 0.00008
O
0.04
N
0.06
1
0.08
=
0.09
&^
0.28
C
0.41
b
TSSA_B
(0.04
) 0.04
O
0.05
N
0.07
1
0.08
=
0.12
&^
0.15
C
0.38
c
SMILES
N 0.04
n 0.03
=
0.04
2
0.05
O
0.05
1
0.08
)
0.01
(
0.01
c
0.23
C
0.19
d
TSSA_M
(0.01
) 0.01
N
0.06
1
0.08
=
0.11
&^
0.24
C
0.41
e
TSSA_S
(0.04
) 0.04
O
0.05
N
0.05
1
0.07
=
0.13
&^
0.13
C
0.40
f
SELFIES
[=O]
0.03
[O]
0.03
[N]
0.05
[=Branch1]
0.06
[Branch1]
0.07
[Ring1]
0.09
[=C]
0.12
[C]
0.38
