# Supplementary material for: t-SMILES: a fragment-based molecular representation framework for de novo ligand design
Source: Nat Commun. 2024 Jun 11;15:4993. doi: 10.1038/s41467-024-49388-6 (PMC11167009; doi:10.1038/s41467-024-49388-6)
Supplement: Supplementary file 4 — Source Data [file 41467_2024_49388_MOESM4_ESM.zip › Soure data/Soure data for Table, Main Text And Curvers/Fig.3 Novelty_FCD/Fig.3 Novelty_FCD.pdf]

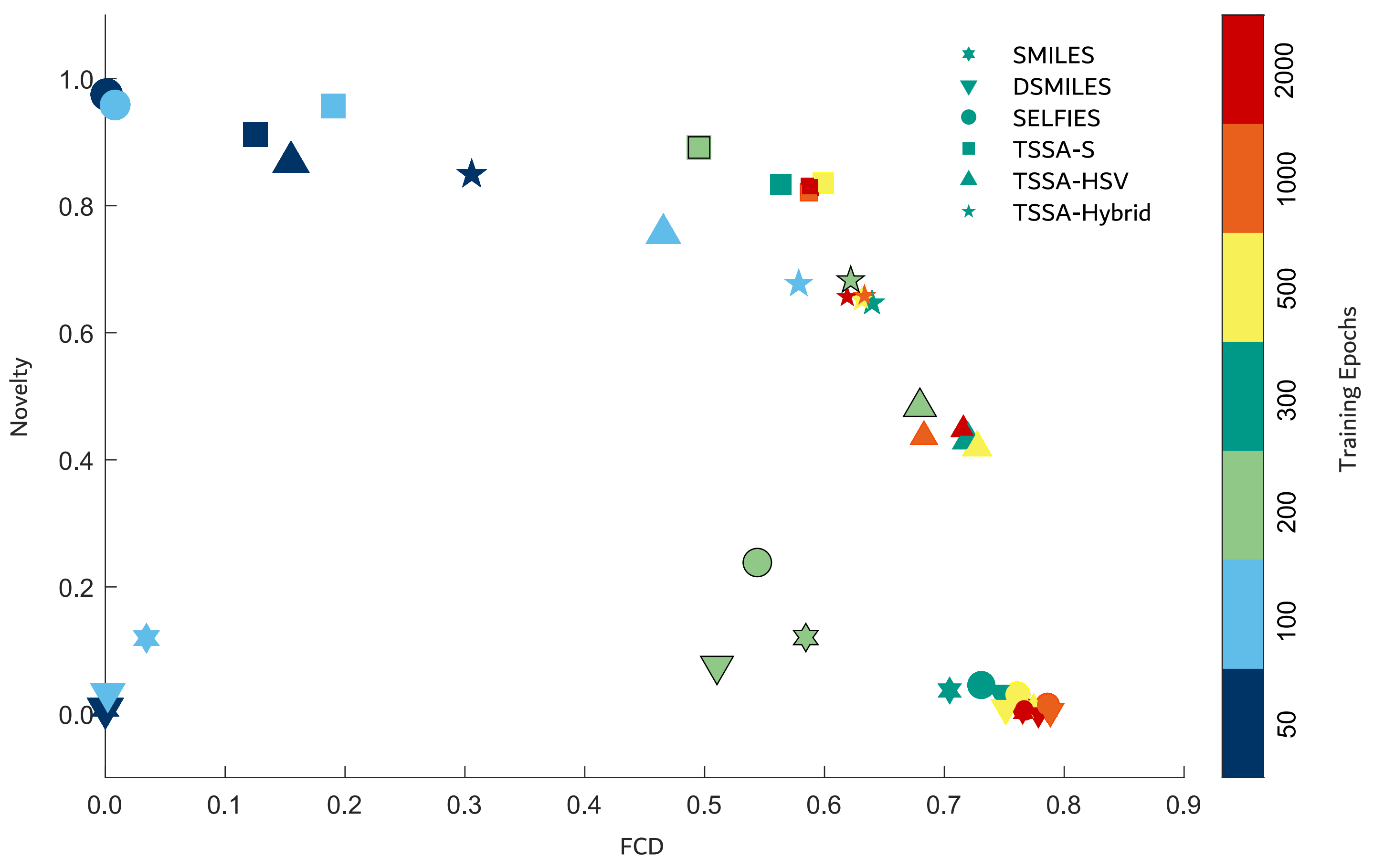
SMILES
DSMILES
SELFIES
TSSA-S
TSSA-HSV
TSSA-Hybrid
Novelty
FCD
Training Epochs
0.0
0.2
0.4
0.6
0.8
1.0
0.1
0.3
0.5
0.7
0.9
50
100
200
300
500
1000
2000
