# Supplementary material for: t-SMILES: a fragment-based molecular representation framework for de novo ligand design
Source: Nat Commun. 2024 Jun 11;15:4993. doi: 10.1038/s41467-024-49388-6 (PMC11167009; doi:10.1038/s41467-024-49388-6)
Supplement: Supplementary file 4 — Source Data [file 41467_2024_49388_MOESM4_ESM.zip › Soure data/Soure data for Table, Main Text And Curvers/Fig.5_SI.Fig.9_SI.Fig.10_SI.Fig.11_SI.Fig.12_SI.Fig.14 Goal Score/Fig.5 Goal_Score.pdf]

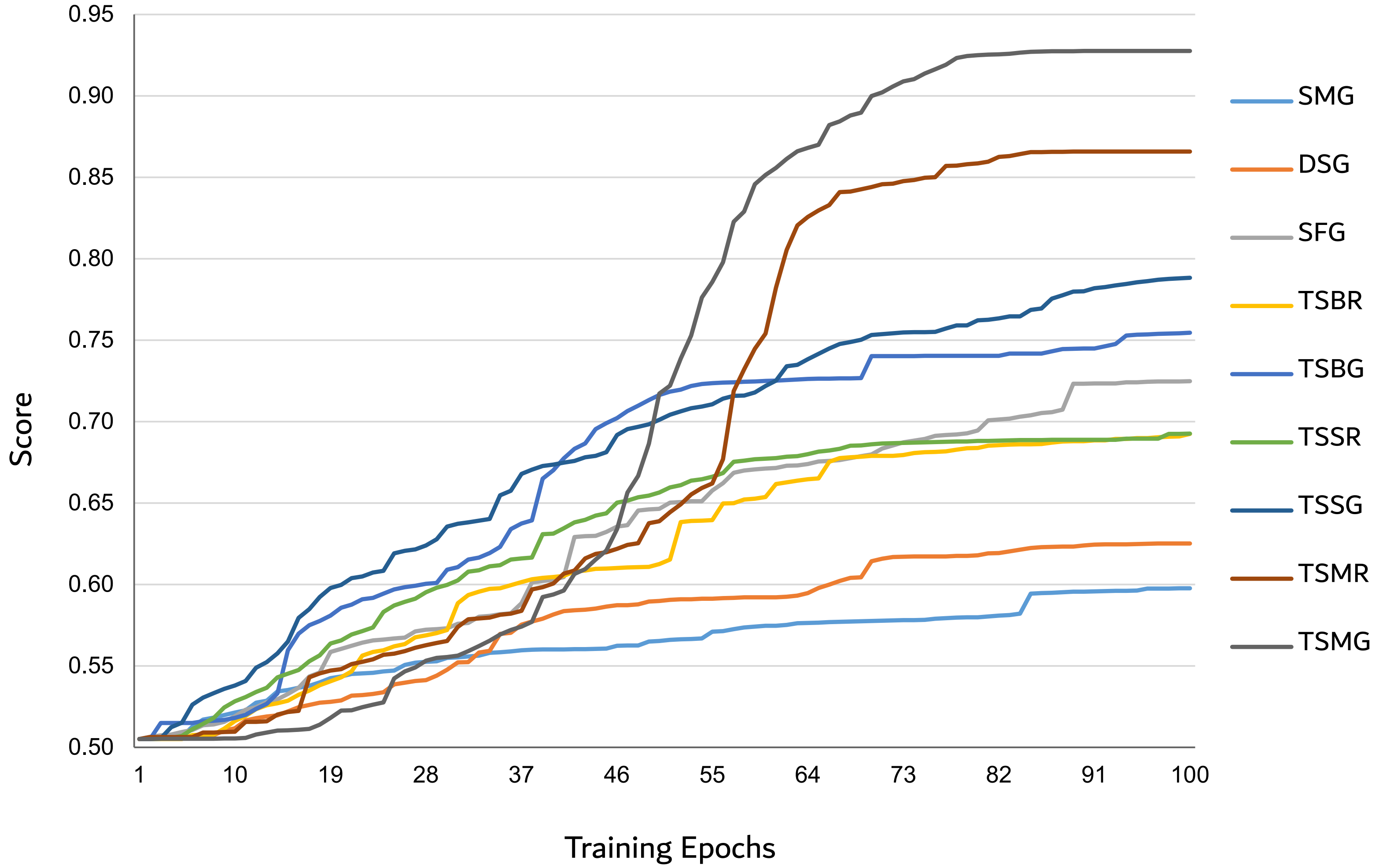

0.95
0.90
0.85
0.80
0.75
0.70
0.65
0.60
0.55
0.50
Score
1
10
19
28
37
46
55
64
73
82
91
100
Training Epochs
SMG
DSG
SFG
TSBR
TSBG
TSSR
TSSG
TSMR
TSMG
